# Exposure to multiple ion beams, broadly representative of galactic cosmic rays, causes perivascular cardiac fibrosis in mature male rats

**DOI:** 10.1371/journal.pone.0283877

**Published:** 2023-04-26

**Authors:** Marek Lenarczyk, Amy Kronenberg, Marylou Mäder, Richard Komorowski, John W. Hopewell, John E. Baker

**Affiliations:** 1 Radiation Biosciences Laboratory, Medical College of Wisconsin, Milwaukee, WI, United States of America; 2 Lawrence Berkeley National Laboratory, Berkeley, CA, United States of America; 3 Department of Radiation Oncology, Medical College of Wisconsin, Milwaukee, WI, United States of America; 4 Department of Pathology, Medical College of Wisconsin, Milwaukee, WI, United States of America; 5 Green Templeton College, University of Oxford, Oxford, United Kingdom; 6 Department of Pharmacology & Toxicology, Medical College of Wisconsin, Milwaukee, WI, United States of America; Georgetown University, UNITED STATES

## Abstract

Long-duration space exploratory missions to the Earth’s moon and the planet Mars are actively being planned. Such missions will require humans to live for prolonged periods beyond low earth orbit where astronauts will be continuously exposed to high energy galactic cosmic rays (GCRs). A major unknown is the potential impact of GCRs on the risks of developing degenerative cardiovascular disease, which is a concern to NASA. A ground-based rat model has been used to provide a detailed characterization of the risk of long-term cardiovascular disease from components of GCRs at radiation doses relevant to future human missions beyond low earth orbit. Six month old male WAG/RijCmcr rats were irradiated at a ground-based charged particle accelerator facility with high energy ion beams broadly representative of GCRs: protons, silicon and iron. Irradiation was given either as a single ion beam or as a combination of three ion beams. For the doses used, the single ion beam studies did not show any significant changes in the known cardiac risk factors and no evidence of cardiovascular disease could be demonstrated. In the three ion beam study, the total cholesterol levels in the circulation increased modestly over the 270 day follow up period, and inflammatory cytokines were also increased, transiently, 30 days after irradiation. Perivascular cardiac collagen content, systolic blood pressure and the number of macrophages found in the kidney and in the heart were each increased 270 days after irradiation with 1.5 Gy of the three ion beam grouping. These findings provide evidence for a cardiac vascular pathology and indicate a possible threshold dose for perivascular cardiac fibrosis and increased systemic systolic blood pressure for complex radiation fields during the 9 month follow up period. The development of perivascular cardiac fibrosis and increased systemic systolic blood pressure occurred at a physical dose of the three ion beam grouping (1.5 Gy) that was much lower than that required to show similar outcomes in earlier studies with the same rat strain exposed to photons. Further studies with longer follow up periods may help determine whether humans exposed to lower, mission-relevant doses of GCRs will develop radiation-induced heart disease.

## Introduction

Human space exploration missions to destinations that may include an asteroid, the Earth’s moon or Mars are actively being planned. Such missions will require humans to live for prolonged periods outside the protection provided by the Earth’s atmosphere and its geomagnetic field. During such exploratory missions beyond low earth orbit, astronauts will be continuously exposed to GCRs, comprised of ions of all naturally occurring elements across a wide energy spectrum. Amongst these are high energy light ions, including protons and helium (^4^He) ions, as well as high atomic number (HZE) ions such as silicon (^28^Si) or iron (^56^Fe). Astronauts may also be exposed to brief, but more intense, periods of predominantly lower energy protons during intermittent solar particle events. The composition of GCRs is roughly 89% protons and 10% ^4^He ions. The remainder is composed of elements heavier than ^4^He [1, 2]. Primary protons of high velocity (100 MeV to 10 GeV) are more sparsely ionizing relative to heavier ions, however, they are of concern for human health due to their abundance in the GCR [3]. Heavy ions (Z≥3), with energies of several hundreds of MeV/n, are very densely ionizing in tissue and thus make a major contribution to the ‘effective’ dose-equivalent in humans in interplanetary space [4]. ^56^Fe ions are among the most prevalent of the HZE ions in GCR [5], although intermediate HZE ions such as ^28^Si ions are also of concern. Little is known about the potential cardiac toxicity from exposure to GCRs. However, degenerative, radiation-induced, cardiovascular disease can follow exposure to conventional photon irradiation (e.g., following breast radiotherapy) and takes many years to develop [6]. Thus, the increased cardiac risk to astronauts would be expected to remain well after they return to Earth. Although it should be recognized that radiotherapy doses are more localized, the same publication showed a similar long-term response was found in the Japanese atomic bomb survivors who received more uniform total body irradiation predominantly from gamma-rays and a small physical dose of neutrons [6].

The relative impact of high energy ion beams, broadly representative of GCRs on the occurrence of cardiovascular disease, is not completely understood. It is essential to understand the individual risk from the different types of charged particles, both sparsely and densely ionizing types, as GCR is comprised of a wide variety of ion species, with large variations in particle energies that deposit energy with different physical as well as associated biological characteristics. To conduct these studies, male WAG/RijCmcr rats were used as they are an established model of radiation injury to the cardiovascular system following photon irradiation [7]. It is reasonable to assume that changes in radiation quality associated with GCRs and/or the physical dose of whole body irradiation would modify the occurrence of cardiovascular disease in the same rat strain. Pathologic cardiac remodeling, as a result of whole body irradiation, is characterized by perivascular collagen accumulation. This increased cardiac collagen deposition can lead to ventricular stiffness resulting in contractile dysfunction [8]. The objective of the study was to determine the response of rats of an age that would be representative of early to mid-career astronauts (e.g. 6 months old at the time of exposure) to (i) individual beams of high energy ions and (ii) three rapidly delivered, sequentially switched, beams of high energy ions. Their effects were determined using clinically relevant biomarkers of cardiovascular disease during a long follow up period, along with early changes in levels of circulating cytokines. Markers of renal injury were also included in the study due to the known link between altered kidney function and cardiovascular risk in young rats exposed to photons [9, 10]. Markers of immune system engagement were included in the evaluation, as irradiation of the kidneys with high doses of photons led to both the attachment of circulating immune cells to the endothelium and an increased infiltration of immune cells in glomeruli [11]. These findings are not unique to the kidney; local irradiation of the CNS [12], also indicated delayed attachment of immune cells to the endothelium and their infiltration into CNS tissue. In this study it was shown that three sequentially delivered, rapidly switched, beams of high energy ions (spanning a total dose range from 0.25 Gy to 1.5 Gy), resulted in the development of perivascular cardiac fibrosis at the highest anchor dose of 1.5 Gy and an increase in systemic systolic blood pressure also at the same anchor dose (1.5 Gy) in male WAG/RijCmcr rats followed for 9 months post-irradiation.

## Materials and methods

### Experimental animals

Six month old male WAG/RijCmcr rats (*n* = 12/group), bred at the Medical College of Wisconsin (MCW), were transferred to Brookhaven National Laboratory (BNL) Upton, New York for irradiation. This strain of rat was selected for this study because previous studies have shown that whole body with low linear energy transfer (LET) radiation, results in the development of cardiovascular disease in a time- and dose- dependent manner [13]. Six month old rats are thought to correspond to middle aged humans [13] representative of the astronaut corps. The rats were allowed to acclimatize at BNL for 2 weeks prior to irradiation. Animals were housed in pairs and maintained on rat chow (Teklad 8904, Indianapolis, IN) and water *ad libitum*. The rats were maintained on a 12 hour light, 12 hour dark cycle at a temperature of 20 ± 1°C, and a relative humidity of 50–80%. Animal studies were conducted in compliance with the US National Research Council’s Guide for the Care and Use of Laboratory Animals, the US Public Health Service’s Policy on Humane Care and Use of Laboratory Animals, and Guide for the Care and Use of Laboratory Animals, in accordance with NIH guidelines. The Animal Care and Use Committee at MCW (protocol # AUA4086) and BNL (protocol # 482) approved all experiments involving live animals, and all efforts were made to minimize suffering. The study personnel and veterinary staff monitored the animals daily in accordance with the AAALAC requirements; no animals died or showed signs of suffering prior to 270 days of follow-up. The start of the study was defined as the time the rats were irradiated or sham treated. At the end of the follow-up period rats were anesthetized with sodium pentobarbital and the heart and kidneys excised for histological investigations. This procedure results in euthanasia.

### Irradiation conditions and dosimetry

High energy charged particle irradiation beams are produced at the NASA Space Radiation Laboratory (NSRL) at BNL. Irradiations with protons alone were performed on October 30, 2015. Irradiations with ^28^Si ions alone were performed on May 6, 2016. Irradiations with ^56^Fe ions alone were performed on June 19, 2015. All irradiations with the rapid switching of all three ion beams were performed on May 31, 2017.

A 20 x 20 cm, uniform irradiation field was used for these studies and the rats were positioned in the initial plateau region of the depth dose distribution in the field from the different ion beams provide by the NSRL facility. This produced a homogenous dose distribution of high energy charged particles within the rats [14]. The primary method of calibrating the dose delivered at NSRL was by the use of an EG&G calibration ion chamber (Far West Technology, Goleta, CA.). Dosimetry was performed by the Physics staff at NSRL [15]. Sham-irradiated groups were included for each study.

Un-anesthetized rats (*n* = 12/group) were immobilized in 6 cm wide Plexiglas restraint jigs for the period of irradiation, with the flank of each animal being perpendicular to the incoming particle beam. A single rat was positioned in each jig, which was placed in the initial ‘plateau region’ of the Bragg-peak depth dose distribution of each ion beam (S1 Fig in S2 File), with two jigs (vertically stacked) in the irradiation field at the same time. This geometry ensured a uniform depth dose distribution across the width of the animal for each beam [16]. Rats of the same age were sham irradiated in the same Plexiglas jigs (*n* = 12/group) to serve as controls. After irradiation or sham-irradiation the rats were maintained in micro isolator cages for the duration of the study. Animals were returned to MCW approximately one week after irradiation or sham-irradiation.

### Irradiation with protons, ^28^Si ions or ^56^Fe ions (single ion beam studies)

Six month old male rats received whole body irradiation with either single doses of protons (1000 MeV, LET = 0.22 keV/μm: doses, 0.25, 0.5, 1.0 or 1.5 Gy), ^28^Si ions (500 MeV/n, LET = 57 keV/μm: doses 0.25, 0.5, 0.75 or 1.5 Gy), or ^56^Fe ions (600 MeV/n, LET = 184 keV/μm: doses 0.1, 0.25, 0.5 or 1.0 Gy). The lower dose range for each of the studies spans the dose range likely to be relevant for a sample Mars mission and included a high anchor dose 1.0 or 1.5 Gy, to benchmark the low dose studies, with NASA’s approval. All doses were delivered within 10 minutes.

### Irradiation with sequential, rapidly switched beams of protons, ^28^Si ions and ^56^Fe ions (three ion beam grouping)

Six month old male rats were irradiated sequentially with three individual charged particle beams delivered, with rapid beam switching, in the following order: 1000 MeV protons (80% of the total dose to each rat, LET = 0.24 keV/μm), 500 MeV/n ^28^Si ions (10% of the total dose, LET = 54 keV/μm) and 600 MeV/n ^56^Fe ions (10% of the total dose, LET = 190 keV/μm). The four total dose groups were: 0.25 Gy, 0.5 Gy, 0.75 Gy, and 1.5 Gy. The switching time between beams was 1–2 minutes. The dose-rates for the two heavy ions were adjusted to deliver a minimum number of ion pulses (spills) to to ensure uniform exposures. For protons, the dose-rate was in the range between 45–54 cGy/min for all dose groups. All doses were delivered within 10 minutes, inclusive of the time required for switching between the different beams.

### Indicators of risk for cardiac disease

Blood (600–1000 μl) was withdrawn by venipuncture from the jugular vein at 30 day intervals, beginning at 30 days up to 270 days after whole body irradiation. Blood was also taken from sham-irradiated control rats at the same time intervals. Serum was then analyzed for total cholesterol, HDL cholesterol and triglycerides (Wisconsin Diagnostic Laboratories, Milwaukee, WI).

### Kidney injury and metabolic biomarkers

Blood was also withdrawn by venipuncture from the jugular vein, at 30 day intervals beginning at 30 days up to 270 days after whole body irradiation and from sham-irradiated control rats. Serum was analyzed to determine blood urea nitrogen (BUN), creatinine, total protein and albumin, and for electrolyte and fluid balance, liver function and glucose (Wisconsin Diagnostic Laboratories, Milwaukee, WI).

The systemic blood pressure (systolic and diastolic) was measured using a non-invasive photoelectric tail-cuff system (BP-2000, Visitech Systems, Apex, NC) [13] at 270 days after exposure. Un-anesthetized rats were placed in plastic restrainers and the cuff, with a pneumatic pulse sensor, was attached to the tail. Rats were allowed to adjust to this procedure for 7 days before any blood pressure measurements were actually taken. Blood pressure values were taken, without heating, and was recorded as the average from at least three consecutive readings obtained from each rat. The blood pressure and heart rate of rats were determined between 13:00–17:00 hours for all experiments. The mean blood pressure was calculated from the systolic and diastolic values.

### Cytokines

Blood was withdrawn by venipuncture from the jugular vein, at 30 and 60 days after whole body irradiation with the three ion beam grouping and from sham-irradiated controls. Serum samples were then analyzed to determine the concentration of 27 cytokines using a rat 27 plex discovery assay (Eve Technologies, Calgary, AB). Changes in cytokine levels in irradiated animals were expressed as the percentage change for each individual cytokine relative to its age-matched, sham-irradiated controls.

### Histology

To evaluate potential tissue damage at 270 days after irradiation, the entire heart and both kidneys (*n* = 6/group) were removed from anesthetized irradiated and sham-irradiated rats and fixed by immersion in 10% formalin (v/v), according to standard procedures described elsewhere [13]. Prior to embedding in paraffin wax for histology, the heart was oriented between the base and the apex, and the kidneys oriented along the mid-dorsal plane. The fixed tissue samples were embedded with kidney samples in the coronal orientation and heart samples in the transverse plane. Sections, 4 μm thick, were cut from each block and stained with Masson’s-trichrome blue according to standard methods described elsewhere [13]. Transverse sections of heart tissue were consistently obtained from the middle of each ventricle from each animal. For the kidney, longitudinal slices were obtained for cortex and medulla. Ten sections of entire slices from each heart and kidney were used for morphometric analysis, also as described elsewhere [13]. A total of 14–45 coronary vessels were analyzed per heart. The collagen content of the coronary vessels was defined as the total area of the media plus adventitia that was stained with trichrome, expressed as a percentage of the luminal area. Perivascular cardiac fibrosis was defined as an increase in collagen content in irradiated animals above the value for sham-irradiated animals [9]. Renal collagen content was measured as the area of an entire kidney section, stained with trichrome, expressed as a percentage of the total area of the kidney section.

### Immunohistochemistry

Paraffin-embedded sections were stained immunohistochemically using a DAKO Autostainer Plus automated staining platform (Agilent/DAKO, Santa Clara, CA). The antibodies investigated included: T cells with DAKO Rabbit Polyclonal CD3^+^ (A0452, 1:100), B cells with Cell Signaling Rabbit Monoclonal CD20^+^, (48750, 1:600) natural killer cells with Cell Marque mouse monoclonal CD56^+^ (156R-94, 1:200), and macrophages with EMB Millipore mouse monoclonal (CD68^+^ (MAB1435, 1:100). Pretreatment was performed with citrate buffer for CD3^+^, CD20^+^ CD56^+^ and CD68^+^, using EDTA retrieval. The standard Labeled Streptavidin Biotin approach was applied for detection with all antibodies. Following pretreatment and blocking steps, primary antibodies were incubated for 1 hour at room temperature. Biotinylated secondary antibodies were incubated for 30 minutes at room temperature (anti-mouse Jackson Immuno 715-066-151 and anti-rabbit Jackson Immuno 715-066-152) followed by a 15-minute incubation with streptavidin-HRP (DAKO P039701-2). Antibody visualization was achieved with DAB+ application (DAKO DAB+K346811-2). All slides were counterstained with Modified Mayer’s Hematoxylin (DAKO S3309330-2) and blued with 0.1% ammonium water and mounted with a synthetic mounting media. Omission of the use of primary antibody served as a negative control. Antibodies were tested and validated for immunohistochemistry (IHC) by the Histology Core Facility at Children’s Hospital of Wisconsin (PEN, Director) [17]. The IHC images were digitally recorded using a high-resolution, whole slide scanner (NanoZoomer HT 2.0, Hamamatsu, Japan) at 40x magnification, and the data were reviewed using NDPview (version 2.7.43, Hamamatsu, Japan) for virtual image exploration. The scanned images were imported using Image Analysis Software, (Visiopharm, Denmark) and analyzed at 20x magnification to quantify the DAB expressions. The software was trained to capture DAB-positive areas in the kidney and heart tissue area, using a preset threshold and the linear Bayesian classification [18]. The processed images were pseudo color coded for DAB-positive area and tissue areas for each kidney and heart section are presented and the data are expressed as a percentage of the total tissue area. The IHC images were recorded and analyzed at the Children’s Research Institute Imaging Core at the Medical College of Wisconsin.

### Echocardiography

Left ventricular systolic function was only assessed 270 days after irradiation in the highest physical dose group (1.5 Gy), that was sequentially irradiated with protons, ^28^Si ions and ^56^Fe ions and in the sham-irradiated group. Six rats were used per group, as previous studies with photons had indicated this was a sufficient number of animals to detect changes in radial and circumferential strain using two-dimensional strain echocardiography following photon irradiation with the potentially a higher biologically effective dose of 10 Gy [9]. The results for whole body irradiated rats were compared with data from sham-irradiated rats. Prior to ultrasound measurements, rats were anesthetized with isoflurane (3% for induction, 1–2% for maintenance). Each rat was placed in dorsal or lateral recumbence on a heated blanket to maintain body temperature. The hair on the chest, legs, and arms was then removed using a depilatory agent (Nair) so that the EKG leads and the transducer contacted the skin directly. After hair removal, ultrasound transmission gel was applied to the chest for the echocardiogram. The operator was a professional sonographer, experienced in rat echocardiography, who was double blinded with respect to the treatment allocation. An echocardiograph Vivid 7 (General Electric, Waukesha, Wisconsin, USA) was used with a M12L (11-MHz) linear-array transducer. Closed-chest imaging was performed in the short-axis view at the mid-LV level (level of papillary muscles). The image depth was 2.5 cm and the acquisition rate was 236 frames/second with electrocardiographic gating [9].

### Echocardiography image analysis

Images were processed using EchoPAC Q analysis software (General Electric). The method has been described previously [9]. Briefly, the endocardial border was manually traced at ‘end-systole’ by an experienced operator, blind as to treatment assignment. The software then automatically selected 6 equidistant tissue-tracking regions of interest in the myocardium. The outer border was adjusted to approximate to the border of the epicardium. The software provided a profile of the radial (myocardial deformation towards the center) and circumferential (myocardial deformation along the curvature) strain (expressed as percentage) with time. Measurements of the end systolic radial and circumferential strain were obtained for each of the 6 segments, and the global strain calculated from the average of these values. Three consecutive heart beats were measured, and the average was used for analysis.

### Experimental design and statistical analysis

The number of subjects in each experimental group was derived from a power analysis based on previous experience with the same type of measurements in similar studies [13]. Animals were randomized to each experimental group. The identity of the animals in each experimental group was known to the investigator responsible for initiating and continuing with an intervention, e.g. irradiation. The identity of any animal under an investigation was not known to the investigator performing the experimental measurements or the analysis. These investigators were not the same persons. The study tested the hypothesis that exposure to representative ions found in the GCRs determines cardiac outcomes. The start of the study was defined as the time that rats were irradiated or sham-irradiated. All data were included in the analysis. All outcome values were expressed as the mean ± standard deviation (SD). All statistical analysis between subjects was performed using Sigma Plot® 11.0 software. Exploratory data analysis used a Shapiro–Wilk test [19] followed by an unpaired student’s *t* test to enable 2-group comparisons. Data failing the Shapiro–Wilk test of normality [19] was analyzed by the Mann-Whitney rank sum (U) test [20]. The Bonferroni multiple-comparison correction was used when several statistical tests were being performed simultaneously. The customary threshold for statistical significance (*P* < 0.05) was used in the analysis. Individual ‘p values’ at specific time points in the longitudinal studies are reported in the figures.

## Results

### Whole body irradiation with single ion beams, representative of components of GCRs, did not result in changes in the conventional indicators of cardiovascular disease, the occurrence of cardiac disease, or indicators of kidney damage at doses relevant to space flight

There were no early deaths in irradiated or sham-irradiated rats. All of the subjects were followed throughout the full 270 day follow-up period.

### Indicators of cardiac disease and the incidence of cardiac disease in rats, following exposure to single beams of protons or heavy ions

The levels of total cholesterol and triglycerides, classical risk factors for cardiac disease, increased progressively in the serum of sham-irradiated rats over the 270 day period of the study, compared with values at 30 days after sham irradiation. These risk factors for cardiac disease also increased as a function of time after irradiation of the different groups, but the values were not significantly different from the values of the age-matched, sham-irradiated animals at each time point over the 270 day follow up period, for rats irradiated with either 0.25, 0.5, 1.0 or 1.5 Gy of protons (S2 Fig in S2 File). Total cholesterol and triglycerides were transiently elevated at 30 days after irradiation with 0.50 and 0.75 Gy of ^28^Si ions, compared with age-matched sham-irradiated controls. However, no such early transient changes were seen in the higher doses. At 90 days after exposure to ^28^Si ions, triglycerides were also transiently elevated in all but the 1.5 Gy dose group. There were no additional substantive changes in any of these risk factors for rats exposed to ^28^Si ions from 60 to 270 days after irradiation, regardless of the dose (S3 Fig in S2 File). Risk factors for cardiac disease (total cholesterol and triglycerides) did not show a clear pattern of response over the 270 day follow up period for rats irradiated with 0.1, 0.25, 0.5 or 1.0 Gy of ^56^Fe ions (S4 Fig in S2 File) compared with age-matched, sham-irradiated controls.

Histological sections of the heart were examined 270 days after the start of the study by a cardiac pathologist blinded to the identity of the specimens (RAK). Histologic analysis showed that the coronary vessels and cardiomyocytes from rats exposed to protons, ^28^Si or ^56^Fe ions retained the normal appearance of hearts from age-matched, sham-irradiated rats (S5 Fig in S2 File). There was no statistically significant increase in the perivascular cardiac collagen content, an index of cardiac fibrosis, in the hearts of rats irradiated even the highest individual dose of protons (1.5 Gy), ^28^Si (1.5 Gy) or ^56^Fe ions (1.0 Gy), compared with age-matched sham-irradiated controls (S6 Fig in S2 File).

### Indicators of kidney injury in rats following exposure to either protons or individual heavy ions

Biomarkers of kidney injury, including BUN, total protein levels, and systemic blood pressure, remained largely unchanged in sham-irradiated rats over the 270 day study period. There was no clear pattern of changes in BUN over the 270 day follow up period for rats irradiated with 0.25, 0.5, 1.0 or 1.5 Gy of protons (S2 Fig in S2 File). BUN was transiently elevated at 30 days after irradiation with 0.25, 0.50, 0.75 and 1.5 Gy of ^28^Si ions, compared with age-matched sham-irradiated controls, but with no clear pattern of change. No changes in BUN levels were found in rats irradiated with ^28^Si ions from 60 to 270 days after irradiation (S3 Fig in S2 File). BUN was also not elevated over the 270 day follow up period for rats irradiated with any of the doses of ^56^Fe ions (S4 Fig in S2 File). Similarly, serum total protein and albumin levels remained essentially unchanged in rats irradiated with protons, ^28^Si ions or ^56^Fe ions over the whole study period when compared with age-matched, sham-irradiated rats. In addition, systemic blood pressures (systolic and diastolic) were not elevated 270 days after irradiation with single ion beams of protons, ^28^Si ions, or ^56^Fe ions (S7 Fig in S2 File). Finally, a histologic analysis of sections of the renal cortex and medulla, collected from rats 270 days after exposure to individual beams of protons, ^28^Si ions or ^56^Fe ions, showed a normal appearance when compared with kidneys from age-matched, sham-irradiated rats (S8 Fig in S2 File).

### Whole body irradiation with the three rapidly switched, sequential ion beams resulted in changes in conventional indicators for cardiac disease, evidence of cardiac disease and modest evidence of kidney injury over the dose range studied

**Fig 1:**
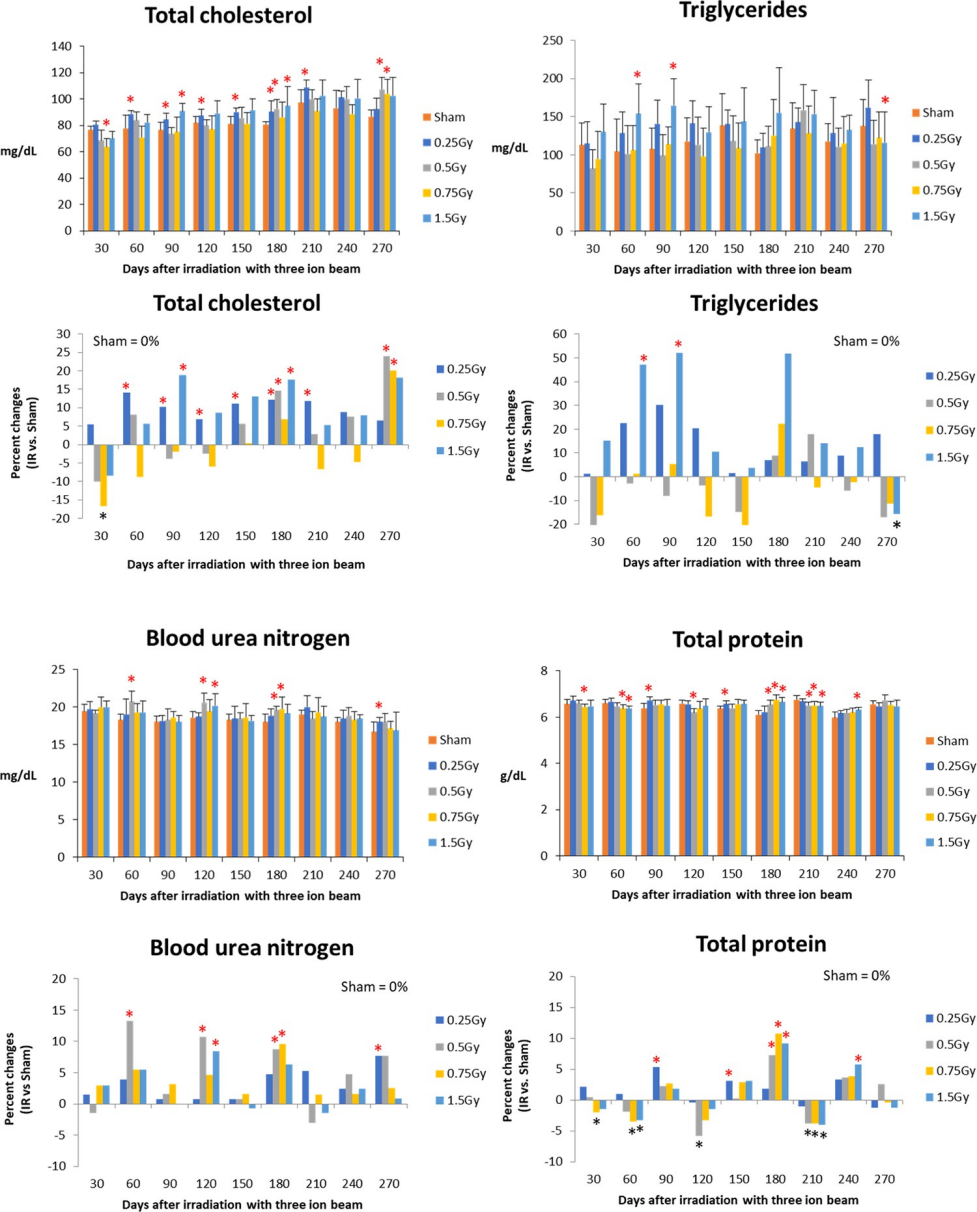
Risk factors for cardiac disease and kidney injury after irradiation of rats with sequentially delivered ion beams of protons, ^28^Si and ^56^Fe. Illustrated are the absolute values and relative changes (relative to age matched controls) in total cholesterol (upper left), triglycerides (upper right), blood urea nitrogen (lower left) and total protein (lower right) in blood after irradiation with total doses at 0.25 Gy, 0.50 Gy, 0.75 Gy or 1.5 Gy [1000 MeV protons (80% of the total dose), 500 MeV/n ^28^Si (10% of total dose) and 600 MeV/n ^56^Fe (10% of the total dose)] or sham-irradiation. Rats were six months of age at the time of irradiation or sham-irradiation. Data represent the mean ± SD, *n* = 7/group. * = p < 0.05 vs. age-matched sham-irradiated control. For relative changes, * denotes levels of risk factors for cardiac disease and kidney injury significantly different from controls.

In rats exposed to rapidly switched, sequentially delivered protons, ^28^Si ions and ^56^Fe ions there were no premature deaths in any dose group, including sham-irradiated controls. Values for total cholesterol were modestly and intermittently increased over the 270 day follow up period after irradiation of rats with 0.25. 0.50, 0.75 or 1.50 Gy of the three ion beam grouping, relative to age matched controls (Fig 1, upper left) but with no clear pattern, while triglyceride levels were essentially unchanged over the whole 270 day follow up period (Fig 1 upper right). The percentage change in total cholesterol and triglycerides, compared with absolute values, are also shown in Fig 1 (upper left and right panels).

**Fig 2:**
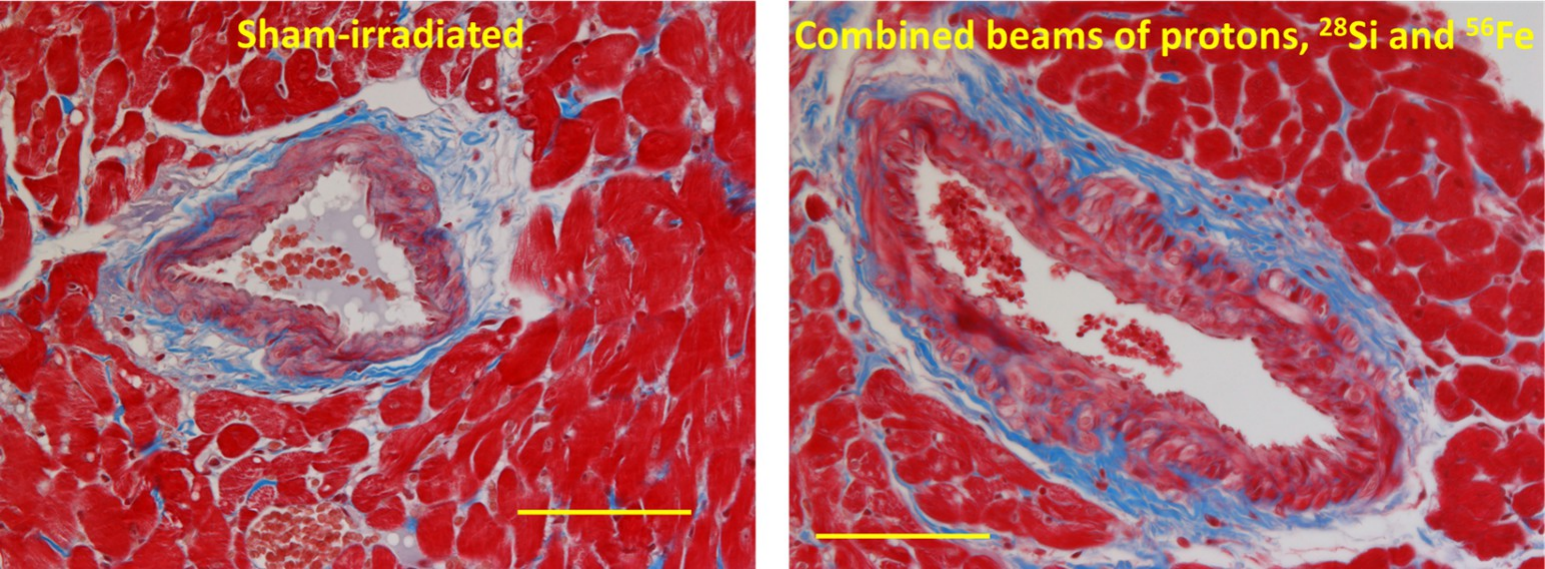
Structural changes to the heart 270 days after irradiation or sham irradiation. Rats were irradiated with 1.5 Gy of sequentially delivered beams of protons (1000 MeV, 80% of the total dose), ^28^Si ions (500 MeV/n, 10% of the total dose) and ^56^Fe ions (600 MeV/n, 10% of the total dose) or sham irradiated. Rats were six months of age at the time of irradiation or sham irradiated. Heart sections were stained with Trichrome. The horizontal bar represents 100 microns. Images are representative of data from six animals per group.

**Fig 3:**
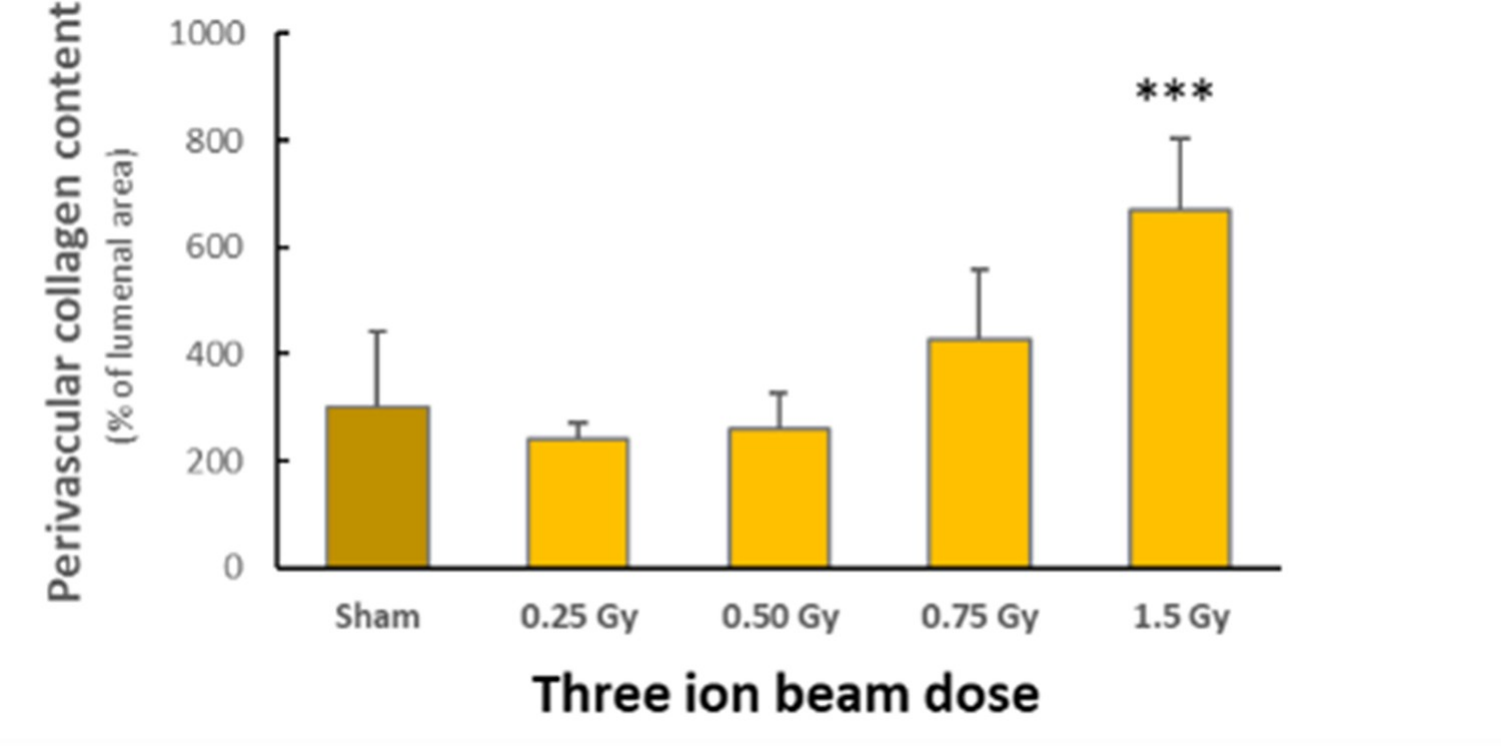
Perivascular cardiac collagen content in hearts 270 days after irradiation or sham irradiation. Rats were irradiated with 0.25, 0.5, 0.75 or 1.5 Gy of the three ion beam grouping of protons (1000 MeV, 80% of the total dose), ^28^Si ions (500 MeV/n, 10% of the total dose) and ^56^Fe ions (600 MeV/n, 10% of the total dose) or sham irradiated. Rats were six months of age at the time of irradiation or sham-irradiation. *** = p < 0.001 vs. sham irradiation. Data are mean ± SD, *n* = 6 /group.

Trichrome staining of sections from hearts collected 270 days after irradiation or sham-irradiation revealed perivascular fibrosis and increased collagen deposition within the wall of the penetrating coronary vessels after whole body exposure to a total dose of 1.5 Gy from the three ion beam grouping (Fig 2). The hearts from age-matched, sham-irradiated rats had symmetrical penetrating vessels with less collagen. Cardiomyocytes from irradiated rats retained a normal in appearance (Fig 2). Exposure of rats to 1.5 Gy, using the three ion beam grouping, resulted in a specific observable effect, manifested as a two-fold increase in the perivascular cardiac deposition of collagen 270 days after irradiation, compared with sham-irradiated, age-matched controls (Fig 3). The incidence of perivascular cardiac fibrosis in rats exposed to a total dose of 1.5 Gy was 100% and reached statistical significance. In contrast, rats irradiated with 0.25, 0.50 or 0.75 Gy of the three ion beam grouping, showed no significant increase in perivascular collagen content compared with the sham-irradiated, age-matched control group after 270 days (Fig 3). This would suggest a threshold dose for the incidence of perivascular cardiac fibrosis between 0.75 and 1.5 Gy for this duration of follow-up post-exposure.

To determine if whole body irradiation with the three ion beam grouping results in any mechanical injury to the heart, both global radial and circumferential strains were measured *in vivo* using 2D echocardiography. At 270 days after whole body irradiation with 1.5 Gy, there were no significant changes in the global radial strain or circumferential strain compared with the age-matched, sham-irradiated control group (S9 Fig in S2 File).

**Fig 4:**
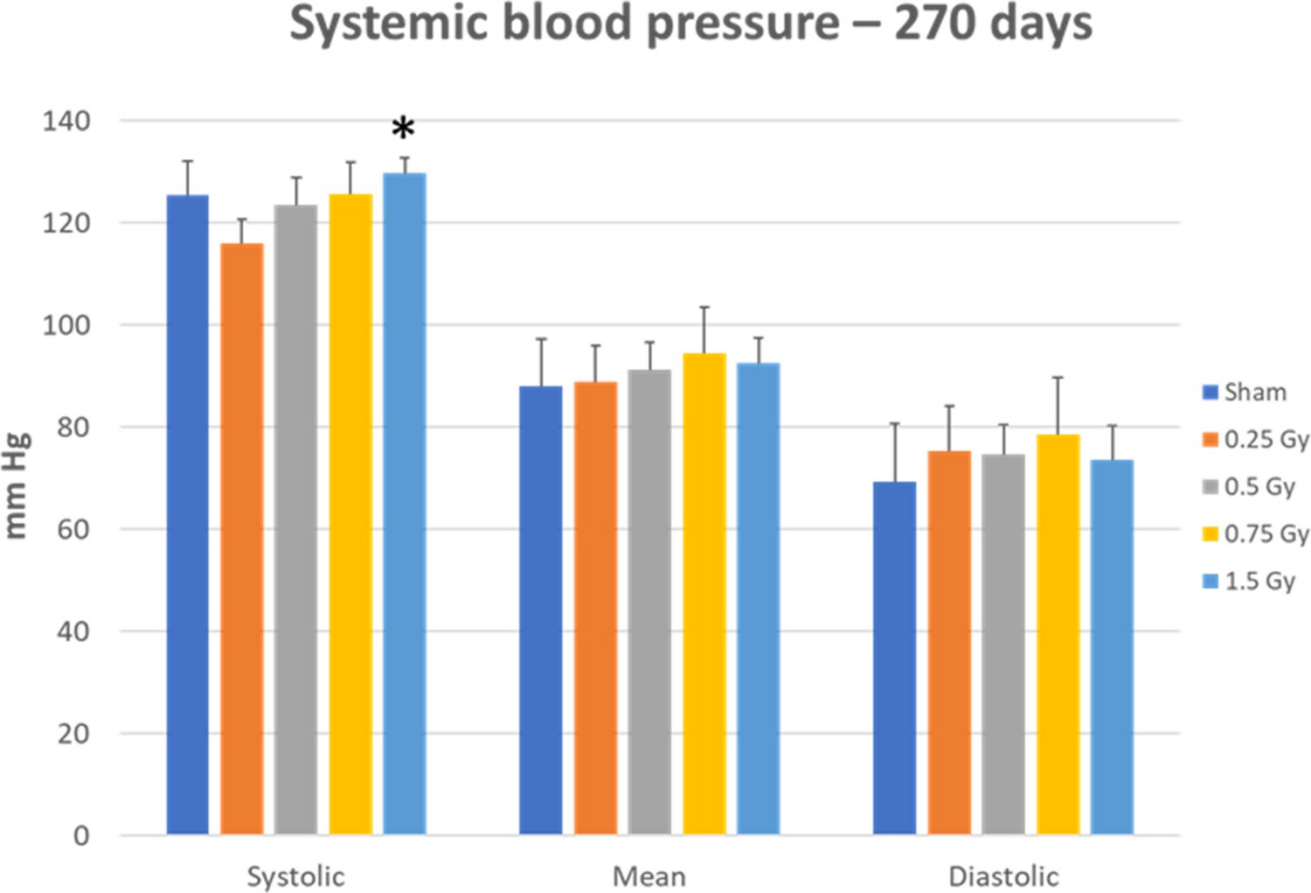
Systemic blood pressure 270 days after irradiation or sham irradiation. Rats were irradiated with either 0.25, 0.5, 0.75 or 1.5 Gy from the three ion beam grouping: protons (1000 MeV, 80% of the total dose), ^28^Si ions (500 MeV/n, 10% of the total dose) and ^56^Fe ions (600 MeV/n, 10% of the total dose) or sham irradiated. Rats were six months of age at the time of irradiation or sham-irradiation. Shown are the systolic, mean and diastolic pressures. Data represents the mean ± SD, *n* = 6/group. * = p < 0.05 vs. sham-irradiated.

As with the indicators for cardiac changes given above, there were variable results for the indication of kidney damage in the cohort of animals exposed to the three sequential ion beam grouping. BUN levels were intermittently increased (Fig 1, lower left), and total protein levels were intermittently increased and decreased (Fig 1, lower right) following the irradiation of rats with 0.25, 0.50, 0.75 or 1.5 Gy of the three ion beam grouping. Somewhat surprisingly, but perhaps very importantly, there were no significant changes seen after the highest dose of 1.5 Gy over the whole observation period. Trichrome staining of histological sections revealed no evidence of renal injury (glomerulosclerosis and fibrosis) 270 days after whole body exposure to the total dose of 1.5 Gy from the three ion beam grouping (S10 Fig in S2 File). There was also no histologic evidence of injury in the kidney in age-matched, sham-irradiated control rats. However, the systemic systolic blood pressure was slightly, but significantly, elevated by 4.1% at 270 days after 1.5 Gy compared with age-matched, sham-irradiated controls (p < 0.05). No changes in systemic systolic blood pressure were seen in rats exposed to the lower doses over this follow-up period (Fig 4).

### Whole body irradiation with three rapidly switched ion beams results in early changes in circulating cytokines

**Fig 5:**
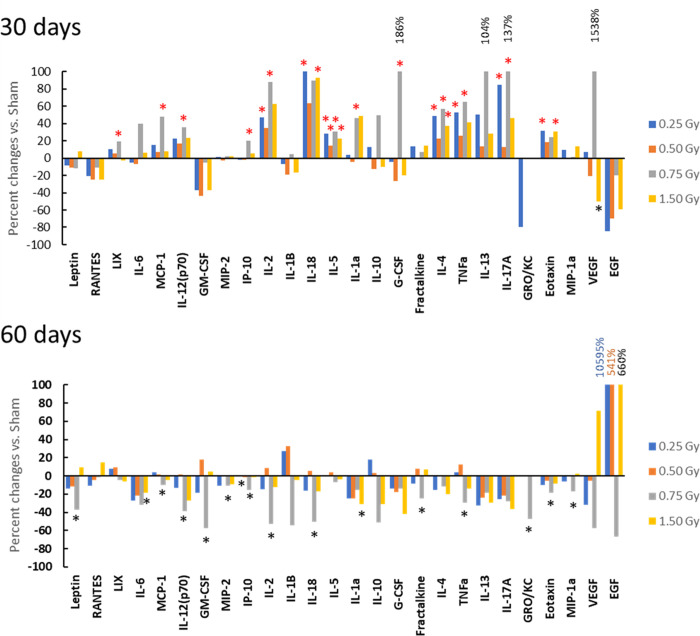
Relative changes in cytokines present in the circulation 30 and 60 days after irradiation. Rats were irradiated with 0.25, 0.50, 0.75 or 1.50 Gy from the sequentially delivered three-ion beam grouping of protons (1000 MeV, 80% of the total dose), ^28^Si ions (500 MeV/n, 10% of the total dose) and ^56^Fe ions (600 MeV/n, 10% of the total dose) or sham irradiated. Rats were six months of age at the time of irradiation or sham-irradiation. Data shown are mean + SD, *n* = 5-12/group. * denotes a significantly increase and * denotes a significant decrease in cytokine levels relative to sham-irradiated aged-matched controls.

Serum samples were collected from rats at 30 days and 60 days after irradiation (0.25, 0.50, 0.75 or 1.5 Gy) or sham-irradiation with the three ion beam grouping. These samples were analyzed for the levels of circulating cytokines. The levels of 27 cytokines were measured using a multiplex cytokine array to identify signaling molecules associated with the underlying mechanism of radiation-induced disease. Of the 27 cytokines accessed, 26 could be reliably quantified (Fig 5).

At 30 days after irradiation, the levels of half the cytokines measured (13/26), were increased in abundance relative to levels in aged-matched controls (Fig 5, upper panel). The levels of six cytokines were increased after a dose of 0.75 Gy; LIX, MCP-1, IL-12 (p70), IP-10, IL-1a, G-CSF. The results for the other dose levels were highly variable. The levels for five other cytokines, IL-2, IL-18, TNFa, IL-17A and Eotaxin were also significantly increased after two radiation dose levels. However, once again, there was no consistency across the other dose groups. Of note, IL-4 levels were increased for 3 of the 4 total radiation dose levels while IL-5 levels were increased for all 4 radiation dose levels. Levels of Leptin, RANTES, IL-6, GM-CSF, MIP-2, IL-1B, IL-10, Fractalkine, IL-13, GRO/KC, MIP-1a and EGF remained unchanged following radiation, regardless of the dose. There was also a significant decrease (of approximately 50%) in one cytokine (VEGF) out of 26 detected (4%) at 30 days but only for one dose of radiation (1.5 Gy). However, for EGF and GRO/KS, the reduction in levels was closer to 80%, but this difference was not found to be significant compared with aged-matched controls.

At 60 days after irradiation, there were no increases in the levels of individual cytokines, relative to age matched controls (Fig 5, lower panel). The relative changes in cytokine expression at 60 days after irradiation are shown (Fig 5, (lower panel). For 15 of 26 cytokines evaluated (58%) a significant decrease was detected compared with the results for sham-irradiated controls. For levels of Leptin, IL-6, MCP-1, IL-12(p70), GM-CSF, MIP-2, IL-2, IL-18, IL-1a, Fractalkine, TNFa, GRO/KC, Eotaxin and MIP-1a the decrease was significant, but only for one dose of radiation. IP-10 levels were significantly decreased for two doses of radiation. The levels of RANTES, LIX, IL-1B, IL-5, IL-10, G-CSF, IL-4, IL-13, IL-17A, VEGF and EGF remained unchanged following radiation.

### Whole body irradiation with three rapidly switched, sequential ion beams results in an engagement of the immune system

**Fig 6:**
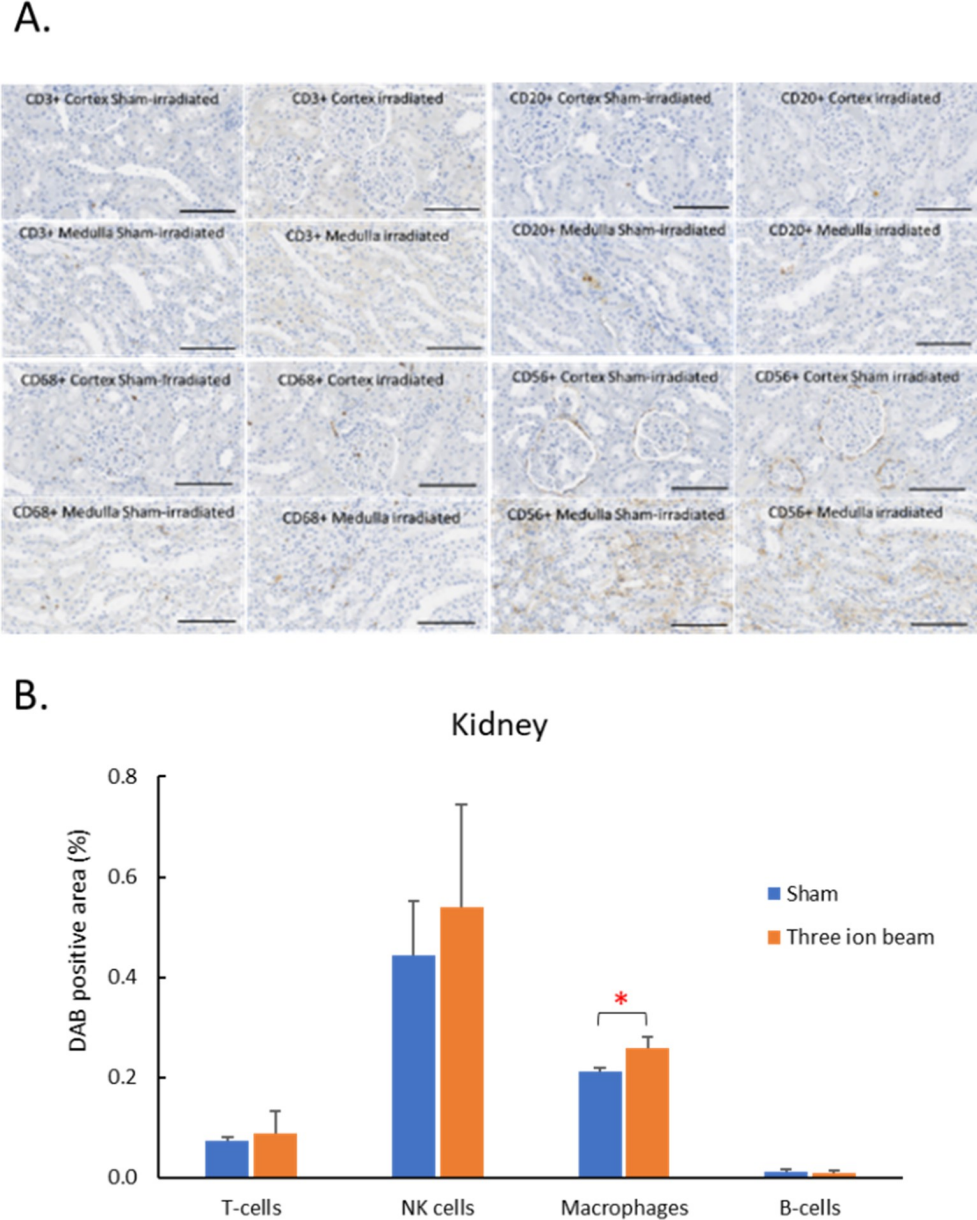
Engagement of components of the immune system in the kidney at 270 days after irradiation or sham irradiation. Rats were irradiated with 1.5 Gy from the three ion beam grouping of protons (1000 MeV, 80% of the total dose), ^28^Si ions (500 MeV/n, 10% of the total dose) and ^56^Fe ions (600 MeV/n, 10% of the total dose) or sham irradiated. Rats were six months of age at the time of irradiation or sham-irradiation. A: T cells (CD3^+^), natural killer cells (CD56^+^), macrophages (CD68^+^) and B cells (CD20^+^) appear as brown colored cells against the blue and white background. The horizontal scale bar represents a distance of 100 microns. Images are representative data from groups of 3–6 animals. B: Quantification of immune cells throughout the kidney. Data represent mean ± SD, *n* = 3–6 animals per group. * = P < 0.05 vs sham-irradiated control.

**Fig 7:**
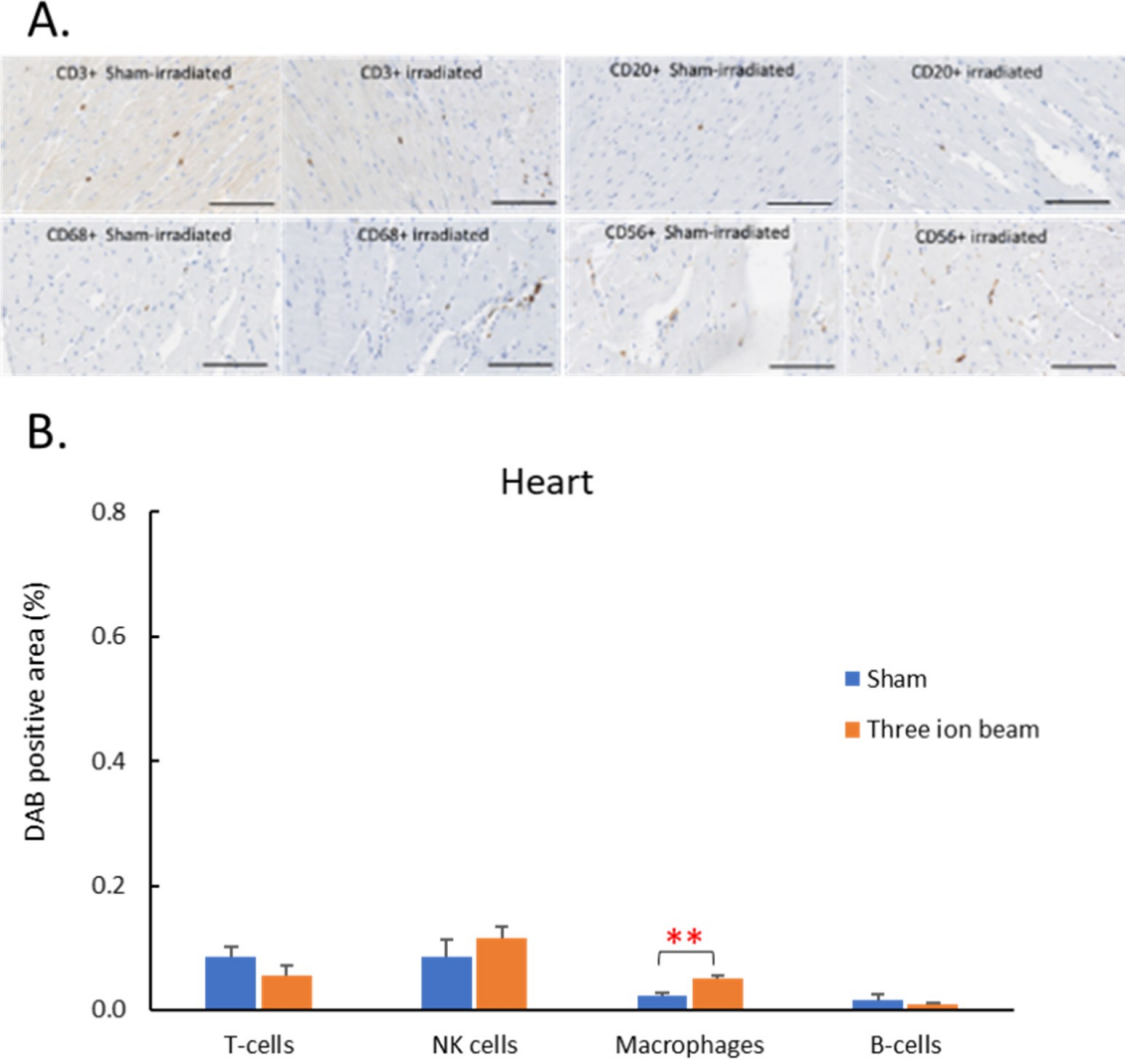
Engagement of components of the immune system in the heart at 270 days after irradiation or sham irradiation. Rats were irradiated with 1.5 Gy from the three ion beam grouping of protons (1000 MeV, 80% of the total dose), ^28^Si ions (500 MeV/n, 10% of the total dose) and ^56^Fe ions (600 MeV/n, 10% of the total dose) or sham irradiated. Rats were six months of age at the time of irradiation or sham-irradiation. A: T cells (CD3^+^), natural killer cells (CD56^+^), macrophages (CD68^+^) and B cells (CD20^+^) appear brown against the blue and white background due to the staining protocols. The horizontal scale bar represents 100 microns. Images are representative from groups of 3 animals. B: Quantification of the number of each type of immune cells in the heart. Data represent the mean ± SD, *n* = 3 animals per group. ** = P < 0.01 vs sham-irradiated control.

To demonstrate the potential role for the infiltration of immune cells on the response of the kidney and the heart to whole body exposure to the three ion beam grouping, IHC was performed on sections of kidney and heart tissue from rats irradiated with a total dose of 1.5 Gy, compared with sham-irradiated controls. In sham-irradiated rats, the biomarkers for macrophages (CD68^+^), T cells (CD3^+^), B cells (CD20^+^), and natural killer cells (CD56^+^) were present in the kidney (Fig 6A and 6B) and heart (Fig 7A and 7B). Whole body irradiation with 1.5 Gy of the three beam grouping resulted in an increased area positive for CD68^+^ in the kidney (p < 0.05) and in the heart (p < 0.01) at 270 days after irradiation, a time when the systemic systolic blood pressure was slightly but significantly increased and the extent of perivascular cardiac fibrosis was also significantly increased. Radiation exposure did not result in an increased area positive for CD3^+^, CD20^+^, or CD56^+^ in the kidney (Fig 6A and 6B) or in the heart (Fig 7A and 7B) at this delayed time point.

## Discussion

The results of the present study identified the development of perivascular fibrosis in the heart and increased systemic systolic blood pressure, a biomarker of kidney injury, as significant outcomes of whole body exposure of middle-aged male rats to a three ion beam grouping broadly representative of GCRs. The results also indicate evidence of a possible threshold dose for perivascular cardiac fibrosis and increased systemic systolic blood pressure at 270 days after this pattern of radiation exposure to be between 0.75 and 1.5 Gy. In contrast, no perivascular cardiac fibrosis or changes in systemic systolic blood pressure were found when rats were exposed to individual charged particle beams. The duration of this rat study was considered to correspond to a period of roughly 20 years of elapsed age in astronauts [21].

Astronauts are expected to be exposed a cumulative dose of 0.3–0.45 Gy from GCRs during a single return mission to Mars [14]. The highest doses from single ion beams of protons, ^26^Si and ^56^Fe and the sequentially delivered three ion beam groupings of protons (80%), ^26^Si (10%) and ^56^Fe ions (10%) of 1.5 Gy, in the present study, are all higher than the maximum dose that an astronaut would be expected to be exposed to during such an exploratory mission. Each of the highest doses used in the present study was reviewed and specifically approved by NASA to serve as an ‘anchor’ dose for the present studies. Statistical significance changes were only achieved using the anchor dose with the 270 day post-exposure observation period of these studies. A longer follow-up period may reveal deleterious cardiovascular outcomes at lower doses. It will be important to address this point, as astronauts are expected to live more than 20 years after completing their exploration class missions.

For perivascular cardiac fibrosis, the changes observed were comparable to those found after the irradiation of male rats of the same strain and of similar age and follow-up period with 10 Gy of sparsely ionizing photons, as demonstrated in earlier studies [7, 13]. In the present study the increase in perivascular collagen occurred after a much lower total dose of 1.5 Gy from the three sequential ion beam grouping, as compared with the 10 Gy of photons. This data can be used to estimate a relative biological effectiveness (RBE) value of ~6.7 for a 2 fold increase in perivascular cardiac fibrosis in mature male rats. For an increase in systemic systolic blood pressure, a statistically significant increase was found at nine months after exposure to 1.5 Gy for the three ion beam grouping. However, the observed increase in systemic systolic blood pressure in the three ion beam study was small and a direct comparison with existing photon data was not possible. Nonetheless, the systemic systolic blood pressure results from the three ion beam study established this endpoint as an indicator of cardiac risk for GCR relevant exposures. Taken together, the results for perivascular cardiac fibrosis and for systemic systolic blood pressure changes reported for the anchor dose of 1.5 Gy indicate a cardiac risk for astronauts who may be exposed to the unique and complex radiation fields associated with long-duration space flight [10, 12]. This result contrasts with the observations obtained for single ion beam studies (protons, ^28^Si ions or ^56^Fe ions) with doses of 0.25–1.5 Gy, where traditional indicators of risk for cardiac disease and renal injury remained essentially unchanged over the 270 day follow up period. Earlier studies by others [22] support this finding; whole body irradiation of Long Evans rats, with a single ion beam of protons (250 MeV, 0.5 Gy) or ^16^O (600 MeV/n, 0.5 Gy) did not change cardiac function or result in cardiac fibrosis.

Rats in the present study, exposed to the three ion beam grouping of protons, ^28^Si ions and ^56^Fe ions, showed modest changes in some but not all of the classical risk factors for cardiac disease and kidney injury over the 270 day follow up period. Moreover, early changes in the levels of circulating cytokines were also found, with an increase after 30 days and a decrease after 60 days. There was no clearly defined dose-dependency in the overall pattern of circulating cytokine levels at 30 or 60 days after irradiation with the three ion beam grouping. Future studies are needed to define the potential involvement of cytokine changes throughout the whole 270 day follow up period to determine whether they can be developed as biomarkers that may predict of the development of cardiovascular disease, as opposed to indicators of radiation exposure.

Irradiation with photons is associated with the infiltration of select immune cell types into tissues [11, 23, 24]. The present results for the three-beam grouping showed that exposure to ions broadly representative of components of GCRs modifies the immune response in the heart and kidneys in rats evident at 270 days after irradiation. The observation is specific to one cell type: an increased abundance of macrophages in the heart and kidney. Increased macrophage infiltration in this study is associated with an alteration in kidney function (manifest as elevated systemic systolic blood pressure) and an increased perivascular collagen content in the heart. However, increased macrophage infiltration alone may not be sufficient to cause fibrosis, a common feature of radiation injury, because although perivascular fibrosis was present in the heart, there was none in the kidney. It is believed that this is the first demonstration of the involvement of macrophages in the kidney caused by GCR-like radiation at the doses used. The engagement of other components of the immune system may be necessary, in addition to macrophages, to cause kidney pathology following irradiation with components of GCRs. In support of this suggestion, irradiation of the kidneys with 10 Gy of X-rays in WAG/RijCmcr rats resulted in infiltration of T cells, natural killer cells, and macrophages is associated with nephropathy [25]. The 270 day follow up period used here may also have been of insufficient duration to detect other changes in immune cell recruitment to the kidney needed to elicit the development of structural changes in the kidney and a greater increase in blood pressure.

Other studies have shown that components of GCRs activate the immune system in the heart. Exposure of Long Evans rats (male, 6 months of age) to a single beam of ^16^O ions (600 MeV/n, 0.5 Gy) increased the protein levels of known T lymphocyte markers (CD2^+^, CD4^+^ and CD8^+^) and macrophages (CD68^+^) in the heart as measured by Western blotting, when compared with age-matched, sham-irradiated controls [22]. In that study, immunochemistry did not reveal an increase in the number of CD68^+^ cells [22]. Exposure of male C57BL/6J mice to individual beams of protons (150 MeV, 1.0 Gy) or ^16^O ions (600 MeV/n, 1.0 Gy) or two sequentially delivered ion beams (protons followed by ^16^O ions) increased the protein levels of the T cell marker CD2^+^ in the heart compared with age-matched, sham-irradiated controls after 270 days [26]. Moreover, in that study, CD68^+^ protein levels in the heart were increased following irradiation with a single ion beam of protons (150 MeV, 1.0 Gy) or ^16^O ions (600 MeV/n, 1.0 Gy). When two ion beams (protons and ^16^O ions) were delivered sequentially to the mice, CD68^+^ protein levels were not increased [26], a result that appears to diverge from the results for rats exposed to the three ion-beam grouping in the present study. In another study [27], exposure of C57Bl/6NT mice to a single beam of protons (1000 MeV, 0.5 Gy) increased the number of CD68^+^ cells in the heart after 7 and/or 28 days and with a single ion beam of ^56^Fe (1000 MeV/n, 0.15 Gy) after 14 days. There was no increase in the number of CD68^+^ cells in the heart after single ion beam exposure from protons or ^56^Fe after 84 days [27], however there was a relatively short follow up period in this study. Taken together, these findings show that the infiltration of CD68^+^ is a characteristic response of the heart to acute low dose exposure from highly energetic charged particle beams that represent individual components of GCRs.

Previous studies delivered two ion beams in sequence to examine their likely effect on the induction of cardiac fibrosis in mice [26, 28]. While whole body irradiation of mice with single ion beams of protons (150 MeV, 0.5 Gy) or ^16^O (600 MeV, 0.15 Gy) caused cardiac fibrosis after a 270 day follow up period [26], the sequential delivery of protons (150 MeV, 0.5 Gy) and ^16^O (600 MeV, 0.15 Gy) to the same total dose did not produce cardiac fibrosis after the 270 day follow up period [26]. This is in contradistinction to the present results with the three ion beam grouping in mature male rats. However, in another study [28], the sequential delivery of two ion beams (protons 1000 MeV, 0.17 Gy followed by ^56^Fe 1000 MeV, 0.15 Gy) was used to investigate cardiac fibrosis in mice. No evidence of cardiac fibrosis was found with protons followed by Fe ions, while delivering ^56^Fe ions before protons did produce cardiac fibrosis after 90 days. Moreover, the total radiation doses of 0.65 Gy in [26] and 0.32 Gy in [28] were less than the highest dose of 1.5 Gy in the current rat study with protons, ^28^Si ions and ^56^ Fe ions, suggesting that dose and sequence of ion beam delivery may be important determinants of outcomes that may also be species- and/or strain-specific. However, in any deep space mission, such sequencing would be random to the individual concerned. Additional, carefully designed dose-effect studies would be useful mechanistically, also allowing a better estimate of the effects of lower doses [29].

The presence of injury to the kidneys is a known risk factor for cardiovascular disease. The kidney is a relatively radiosensitive organ, requiring a lengthy time course for the development of radiation damage [30, 31]. Photon exposure is associated with a dose-related risk of nephropathy, proteinuria and hypertension [32]. In the present study, systemic systolic blood pressure, an index of kidney injury, was increased by a small but statistically significant extent 270 days after irradiation with 1.5 Gy from the three ion beam grouping. Surprisingly, kidney injury was manifest without any significant increase in BUN, and was not accompanied by any gross kidney pathology, although there was an infiltration of macrophages into both the cortex and medulla of the kidney. Leukocyte attachment to the glomerular capillary endothelium is a primary event in renal radiation injury as shown previously after photon irradiation [11]. Future studies are needed to determine whether this phenomenon contributes to kidney and heart effects resulting from space-relevant radiation exposure.

Renal dysfunction has been proposed as part of the mechanism causing increased cardiovascular disease in cancer survivors treated with conventional photon treatment following both local [6] and whole body irradiation [33] and in survivors of atomic bomb exposures [6, 34]. Published studies with higher doses of X-rays (e.g. 10 Gy) indicate that kidney injury is associated with the appearance of vascular pathology in the heart [9, 10]. Additional studies are needed to determine whether radiation injury to the kidneys caused by sequential exposure to ion beams broadly representative of GCRs is likely to be a factor in causing cardiac disease at space mission relevant doses.

The end-points used in the current study are directly relatable to clinically relevant markers of human cardiovascular diseases and to the significant pathology related to late disease. The traditional biomarkers used in the present study follow the current standard-of-care clinical practices and included Framingham risk factors such as blood cholesterol [35, 36]. Total cholesterol is a clinically accepted risk factor for cardiac disease in humans. Here, an age-related trend was shown in total cholesterol in sham-irradiated rats (76 mg/dl at 30 days to 87 mg/dl at 270 days). This age-related increase in total cholesterol is also present in humans of astronaut relevant age [37]. Species differences in cholesterol metabolism do exist between rats and humans. Serum total cholesterol in rats is comprised of ∼90% HDL cholesterol, while serum total cholesterol in humans is comprised of ∼40% HDL cholesterol. This difference is due in part to rat serum containing very little LDL cholesterol compared to human serum [38].

High dose studies with X-rays also demonstrated that total cholesterol was a relevant indicator of risk for cardiac disease in male, middle aged rats of the same strain used in the present study [7]. The current findings indicate modest increases in both the absolute and relative values for total cholesterol. Total cholesterol levels were increased after irradiation with the three ion beam grouping but not in a consistent manner.

The infiltration of Inflammatory cells is an important component of the response of tissues to radiation [11, 12, 39–41]. Biomarkers of inflammation have been reported to be elevated in astronouts during space flight, however space flight imposes many systemic stressors on humans in addition to radiation exposure, including microgravity and circadian rhythm disruption [42–44]. Inflammatory cytokines have been associated with the progression of cardiovascular disease in large cohorts of humans on Earth [45, 46], independent of the more conventional risk factors [47, 48]. In the present study, early changes (30 days and 60 days after irradiation) in cytokines were monitored in rats exposed to 0.25, 0.50, 0.75 and 1.5 Gy of the three ion beam grouping, in which an evaluation for potential cardiac disease was also undertaken. The cytokines measured were charcteristic of the inflammatory process; some are pro-inflammatory and some such as IL-10 are anti-inflammatory. The panel of potential biomarkers examined included the cytokines measured in the 340 day NASA Twins study [44], and in astronauts living aboard the International Space Station [42]. The present study showed that some biomarkers of inflammation are substantially altered at early times after irradiation with representative components of GCRs. These changes reflect prior exposure to radiation, but it was not possible in this initial study to examine changes across the full 270 day follow up period. The relationship of later changes in inflammatory modulators to outcomes from GCR exposure remains unknown.

The present study shows that serum levels of the pro-inflammatory cytokine IL-18 is elevated 30 days after whole body exposure to the three ion beam grouping. Previous studies have shown IL-18 to be elevated 20 days after the local irradiation of the kidneys with a dose of 10 Gy of photons in the WAG/RijCmcr rat, and IL-18 is also associated with out-of-field effects in rats receiving local kidney irradiation manifest as disease in the sham-irradiated heart [10]. IL-18 is reported to be elevated after total body exposure to photons in mice (5–12 Gy), mini-pigs (1.6–1.78 Gy) and non-human primates (7 Gy) [49]. Moreover, the cytokine IL-17A was also elevated 30 days after irradiation with no change at 60 days after irradiation. IL-17A stimulates cardiac fibroblast proliferation [50], a component of the pathway resulting in cardiac fibrosis, and is of interest in the consideration of GCR-induced cardiovascular disease. Circulating levels of IL-4 increased after 3 of the 4 doses of radiation at 30 days. Photon irradiation of rats causes production of IL-4 in lung 28 days after radiation [51]. Circulating levels of IL-5, a pro-inflammatory cytokine, were increased for all four radiation dose levels after 30 days. IL-5 is upregulated in irradiated tumor models in mice [52]. These early cytokine releases most likely reflect a direct effect of exposure to radiation. Other laboratories have shown patterns of responses of a pro-inflammatory nature can recur in a cyclical fashion over succeeding days, weeks and months after irradiation [53], forming a persistent cascade of cytokine expression [54]. The present preliminary studies suggest that sequential exposure to the three ion beam grouping of representative components of GCRs result in an early “up” and “down” response in cytokine levels. At the present time, there is no single biomarker that is prognostic of cardiovascular pathology [55].

In the present study, no changes were seen in cardiac mechanical function using global radial or circumferential strain as measured by 2D echocardiography in rats exposed to the three ion beam grouping, even in the presence of perivascular cardiac fibrosis after 1.5 Gy. This inability to detect changes in mechanical cardiac function may be due to the limited sensitivity of existing echocardiography methods. Cardiac magnetic resonance tagging [56] allows for the quantitative assessment of myocardial contractility on a regional basis. As regional changes in myocardial contractility frequently occur before cardiac damage [57, 58], this approach may overcome limitations of echocardiography. A recent study that used cardiac magnetic resonance techniques showed that cardiac mechanical function was impaired in 6 month old male C57BL/6 mice 12 months after acute exposure to a five ion/six beam grouping (simGCRsim) representative of the GCR [59]. In the same study, transthoracic echocardiography was much less sensitive than the cardiac magnetic resonance studies.

In the present study, ‘astronaut aged’ rats showed increased perivascular cardiac collagen content and systemic systolic blood pressure 270 days following exposure to the highest anchor dose of the three ion beam grouping. Future studies may need to be extended beyond 270 days to detect changes in cardiac and renal endpoints for lower, mission-relevant doses. Such studies will also require larger cohorts to adjust for natural losses usually associated with an aging cohort.

The astronaut corps consist of males and females. In the present study only effects on male rats were assessed. ICRP policy established in 2007 states that gender specific data are not recommended for the general purposes of radiological protection for occupationally exposed individuals [60]. NASA recently asked the National Academy of Sciences to review its approach to radiation protection for deep space missions [61]. In 2022, NASA updated its spaceflight permissible exposure limits (sPEL) and this is universal for all ages and sexes. The updated career limit is 600 mSv based on a 3% mean increased risk of exposure induced death from cancer [62]. There are separate limits for short-term or career non-cancer effects with a 30 day limit of 250 mGy-Eq, a 1 year limit of 500 mGy-Eq and a career limit of 1000 mGy-Eq for the circulatory system [62]. No adjustment is made for crewmember sex or age in the non-cancer risk limits. Future additional studies should be carried out in females, and compared with male rats, to further clarify the present guidelines.

This initial study, on the effects of multiple ion species on the cardiovascular outcomes in the rat, utilized beams in the initial plateau portion of the Bragg peak depth dose distribution. This was to optimize the dose uniformity through the irradiated rats, possible in small animals. It is well established that this region of the Bragg peak depth dose distribution is associated with the lowest RBE values. This has been clearly illustrated by cell survival studies with protons [63]. NASA has considered body size differences between rodents and humans in the subsequent design of two GCR simulators. One is the five ion/six beam simGCRsim configuration used in the recent mouse study of cardiac outcomes [59] and one with 33 beams that includes some stopping ions as would be found in people located in a spacecraft or on a planetary or extra-planetary habitat [14]. For purposes of designing the GCR simulators, it was estimated that the proton fluences will, on average, make ~126 traversals/cell nucleus/year, with He ions averaging ~7 traversals/cell nucleus/year [14]. For the HZE ions, the expected fluence is 0.5/cell nucleus/year. For future work, it will be important to consider the effects of lower fluences delivered over a more protracted time frame to approximate more closely the radiation environments astronauts will experience on long duration exploration class missions to Mars or other locations in the solar system.

## Supporting information

S1 File (PDF)

S2 File (PDF)
