# Supplementary material for: Exposure to multiple ion beams, broadly representative of galactic cosmic rays, causes perivascular cardiac fibrosis in mature male rats
Source: PLoS One. 2023 Apr 26;18(4):e0283877. doi: 10.1371/journal.pone.0283877 (PMC10132632; doi:10.1371/journal.pone.0283877)
Supplement: S1 File — (PDF) [file pone.0283877.s001.pdf]

Minimal underlying data set for Fig. 1

Risk factors for cardiac disease (total cholesterol - Chol, triglycerides - TRIGS) and kidney injury (blood urea nitrogen - BUN, total protein - TP) in the serum of male WAG/RijCmcr rats after whole body exposure to sequentially delivered mixed particles. (protons+28Si+56Fe).

Rat #	Blood collection (Days post exposure)	Total 3-ions Dose (Gy)	BUN mg/dL	TP (g/dl)	Chol (mg/dl)	TRIGS (mg/dl)
9264	30	1.5	20	6.5	66	104
9265	30	1.5	21	6.4	69	73
9266	30	1.5	21	6.4	74	183
9267	30	1.5	19	6.2	61	122
9268	30	1.5	19	6.3	73	153
9269	30	1.5	20	6.5	70	155
9270	30	1.5	20	7.0	77	121
9280	30	0.75	20	6.3	56	70
9281	30	0.75	21	6.6	73	158
9282	30	0.75	20	6.5	62	70
9283	30	0.75	20	6.4	68	108
9284	30	0.75	19	6.4	57	77
9285	30	0.75	22	6.6	60	57
9286	30	0.75	18	6.3	69	121
9296	30	0.5	19	6.4	61	53
9297	30	0.5	19	6.6	63	84
9298	30	0.5	19	6.6	65	66
9299	30	0.5	19	6.7	69	81
9300	30	0.5	20	6.8	85	128
9301	30	0.5	19	6.6	68	67
9302	30	0.5	19	6.5	70	97
9312	30	0.25	18	7.0	79	117
9313	30	0.25	20	6.8	82	149
9314	30	0.25	20	6.7	80	98
9315	30	0.25	20	6.7	81	158
9316	30	0.25	21	6.8	79	80
9317	30	0.25	19	6.6	86	100
9318	30	0.25	20	6.4	77	99
9328	30	0	17	6.6	71	140
9329	30	0	18	6.6	80	174
9330	30	0	22	6.7	74	85
9331	30	0	18	6.5	84	118
9332	30	0	19	6.5	84	114
9333	30	0	22	6.6	66	67

9334	30	0	20	6.5	76	92
9264	60	1.5	18	6.5	84	137
9265	60	1.5	18	6.4	82	137
9266	60	1.5	20	6.5	90	226
9267	60	1.5	19	6.3	72	138
9268	60	1.5	22	6.4	78	137
9269	60	1.5	20	6.3	87	188
9270	60	1.5	18	6.3	82	114
9280	60	0.75	19	6.2	54	60
9281	60	0.75	19	6.2	71	129
9282	60	0.75	20	6.5	73	144
9283	60	0.75	21	6.3	81	138
9284	60	0.75	18	6.6	69	102
9285	60	0.75	20	6.3	72	75
9286	60	0.75	18	6.5	76	94
9296	60	0.5	22	6.4	80	47
9297	60	0.5	22	6.3	77	104
9298	60	0.5	21	6.5	81	90
9299	60	0.5	18	6.5	89	159
9300	60	0.5	20	6.7	93	135
9301	60	0.5	21	6.3	79	83
9302	60	0.5	21	6.6	89	93
9312	60	0.25	20	6.8	90	142
9313	60	0.25	23	6.3	85	143
9314	60	0.25	19	6.8	91	162
9315	60	0.25	18	6.8	86	158
9316	60	0.25	20	6.6	86	130
9317	60	0.25				
9318	60	0.25	18	6.6	91	104
9319	60	0.25	16	6.7	91	85
9322	60	0.25	18	6.7	89	102
9328	60	0	18	6.8	90	97
9329	60	0	18	6.6	66	55
9330	60	0	18	6.7	88	98
9331	60	0	18	6.8	85	153
9332	60	0	20	6.4	71	47
9333	60	0	18	6.4	77	146
9334	60	0	18	6.5	67	136
9264	90	1.5	18	6.6	94	124
9265	90	1.5	17	6.0	87	151
9266	90	1.5	19	6.6	95	184

9268	90	1.5	17	6.4	84	226
9269	90	1.5	19	6.8	98	147
9270	90	1.5	18	6.6	87	154
9280	90	0.75	19	6.3	55	78
9281	90	0.75	18	6.6	74	113
9282	90	0.75	19	6.6	81	102
9283	90	0.75	18	6.8	92	132
9284	90	0.75	18	6.7	76	116
9285	90	0.75	20	6.6	76	105
9286	90	0.75	18	6.3	71	151
9296	90	0.5	19	6.4	71	111
9297	90	0.5	20	6.9	78	94
9298	90	0.5	18	6.6	79	104
9299	90	0.5	18	6.4	77	141
9300	90	0.5	20	6.7	75	108
9301	90	0.5	16	6.3	66	55
9302	90	0.5	17	6.4	68	83
9312	90	0.25	18	6.7	76	88
9313	90	0.25	17	6.5	87	113
9314	90	0.25	18	7.0	85	133
9315	90	0.25	19	6.8	84	177
9316	90	0.25	19	6.7	81	158
9317	90	0.25	18	6.7	91	160
9318	90	0.25	18	6.7	86	155
9328	90	0	18	6.1	75	111
9329	90	0	17	6.4	80	107
9330	90	0	19	6.3	71	141
9331	90	0	18	6.6	82	91
9332	90	0	18	6.5	73	118
9333	90	0	19	6.2	69	58
9334	90	0	17	6.6	85	130
9264	120	1.5	19	6.5	92	119
9265	120	1.5	19	6.1	89	155
9266	120	1.5	22	6.2	91	143
9267	120	1.5	22	6.4	76	78
9268	120	1.5	18	6.5	79	159
9269	120	1.5	21	7.0	105	160
9270	120	1.5	20	6.7	91	94
9280	120	0.75	18	6.4	61	61
9281	120	0.75	17	6.8	79	172
9282	120	0.75	20	6.5	86	77

9283	120	0.75	20	6.3	93	114
9284	120	0.75	19	6.4	73	82
9285	120	0.75	21	6.4	73	76
9286	120	0.75	21	5.8	74	103
9296	120	0.5	23	6.4	75	64
9297	120	0.5	21	6.1	85	101
9298	120	0.5	20	6.0	82	123
9299	120	0.5	19	6.3	80	141
9300	120	0.5	20	6.1	84	174
9301	120	0.5	20	6.4	73	102
9302	120	0.5	21	6.1	80	88
9312	120	0.25	19	6.3	82	109
9313	120	0.25	19	6.6	88	138
9314	120	0.25	18	6.4	81	134
9315	120	0.25	18	6.6	94	105
9316	120	0.25	19	6.6	89	170
9317	120	0.25	19	6.6	88	186
9318	120	0.25	19	6.8	90	147
9328	120	0	19	6.5	80	83
9329	120	0	19	6.8	83	125
9330	120	0	17	6.7	78	158
9331	120	0	18	6.6	88	124
9332	120	0	19	6.5	85	120
9333	120	0	19	6.6	74	69
9334	120	0	19	6.4	85	143
9264	150	1.5	18	6.5	94	111
9265	150	1.5	18	6.6	92	105
9266	150	1.5	18	6.6	91	177
9267	150	1.5	19	6.6	79	82
9268	150	1.5	18	6.3	85	164
9269	150	1.5	19	6.8	106	205
9270	150	1.5	17	6.6	94	160
9280	150	0.75	17	6.5	65	71
9281	150	0.75	18	6.9	86	128
9282	150	0.75	20	6.5	86	138
9283	150	0.75	22	6.6	91	117
9284	150	0.75	17	6.3	77	86
9285	150	0.75	19	6.7	84	69
9286	150	0.75	17	6.4	80	151
9296	150	0.5	19	6.5	83	101
9297	150	0.5	19	6.5	80	132

9298	150	0.5	19	6.1	84	116
9299	150	0.5	18	6.3	88	135
9300	150	0.5	18	6.6	102	176
9301	150	0.5	17	6.3	78	75
9302	150	0.5	19	6.4	84	90
9312	150	0.25	19	6.5	94	142
9313	150	0.25	17	6.7	91	141
9314	150	0.25	18	6.7	90	136
9315	150	0.25	16	6.4	89	131
9316	150	0.25	19	6.4	93	178
9317	150	0.25	21	6.6	89	136
9318	150	0.25	19	6.7	84	119
9328	150	0	18	6.3	76	78
9329	150	0	17	6.5	81	165
9330	150	0	18	6.4	82	119
9331	150	0	18	6.5	92	148
9332	150	0	19	6.3	82	188
9333	150	0	19	6.3	74	97
9334	150	0	19	6.3	80	174
9264	180	1.5	18	6.6	85	149
9265	180	1.5	18	6.6	86	109
9266	180	1.5	20	6.4	100	211
9267	180	1.5	20	6.8	84	73
9268	180	1.5	21	6.6	94	150
9269	180	1.5	19	7.0	126	248
9270	180	1.5	18	6.6	88	147
9280	180	0.75	18	6.7	71	79
9281	180	0.75	18	6.9	86	209
9282	180	0.75	21	7.0	93	153
9283	180	0.75	21	6.8	107	93
9284	180	0.75	21	6.7	82	125
9285	180	0.75	21	6.8	81	79
9286	180	0.75	18	6.4	83	138
9296	180	0.5	20	6.5	86	104
9297	180	0.5	19	6.7	97	124
9298	180	0.5	20	6.2	95	106
9299	180	0.5	20	6.5	97	127
9300	180	0.5	20	6.5	102	154
9301	180	0.5	19	6.8	82	87
9302	180	0.5	19	6.6	88	78
9312	180	0.25	18	6.5	90	87

9313	180	0.25	18	6.5	100	136
9314	180	0.25	19	6.1	91	103
9315	180	0.25	18	6.3	93	126
9316	180	0.25	20	5.8	74	98
9317	180	0.25	20	6.0	92	95
9318	180	0.25	19	6.3	93	122
9328	180	0	19	6.2	81	93
9329	180	0	18	6.3	79	115
9330	180	0	18	6.3	83	114
9331	180	0	16	5.9	77	71
9332	180	0	18	6.1	84	117
9333	180	0	19	6.0	79	95
9334	180	0	18	5.9	81	111
9264	210	1.5	17	6.2	99	147
9265	210	1.5	17	6.4	98	177
9266	210	1.5	20	6.5	106	152
9267	210	1.5	19	6.4	89	110
9268	210	1.5	18	6.7	102	145
9269	210	1.5	20	6.6	127	208
9270	210	1.5	20	6.6	96	134
9280	210	0.75	17	6.3	86	197
9281	210	0.75	18	6.7	83	89
9282	210	0.75	22	6.6	99	120
9283	210	0.75	22	6.5	107	128
9284	210	0.75	18	6.3	80	126
9285	210	0.75	19	6.7	93	97
9286	210	0.75	19	6.4	87	142
9296	210	0.5	20	6.7	95	173
9297	210	0.5	19	6.7	105	200
9298	210	0.5	17	6.4	105	131
9299	210	0.5	19	6.5	105	171
9300	210	0.5	18	6.5	107	189
9301	210	0.5	18	6.4	89	134
9302	210	0.5	18	6.3	94	111
9312	210	0.25	19	6.7	110	152
9313	210	0.25	19	6.8	109	161
9314	210	0.25	19	6.7	117	
9315	210	0.25	23	6.7	113	131
9316	210	0.25	20	6.5	100	131
9317	210	0.25	21	6.6	106	163
9318	210	0.25	19	6.8	107	119

9328	210	0	19	6.8	84	167
9329	210	0	18	7.0	93	166
9330	210	0	19	6.6	112	147
9331	210	0	19	7.0	106	117
9332	210	0	19	6.6	96	120
9333	210	0	19	6.7	90	73
9334	210	0	20	6.6	100	150
9264	240	1.5	18	6.2	97	143
9265	240	1.5	18	6.3	88	120
9266	240	1.5	18	6.2	103	160
9267	240	1.5	19	6.4	91	110
9268	240	1.5	19	6.3	95	133
9269	240	1.5	19	6.5	131	151
9270	240	1.5	18	6.3	98	110
9280	240	0.75	17	6.3	85	74
9281	240	0.75	17	6.4	88	142
9282	240	0.75	19	6.3	91	158
9283	240	0.75	20	6.2	100	139
9284	240	0.75	18	5.8	78	88
9285	240	0.75	18	6.3	92	74
9286	240	0.75	19	6.1	86	131
9296	240	0.5	18	6.1	98	103
9297	240	0.5	21	6.4	100	82
9298	240	0.5	19	6.2	102	81
9299	240	0.5	19	6.3	118	121
9300	240	0.5	18	6.2	103	129
9301	240	0.5	19	6.2	89	146
9302	240	0.5	18	5.9	90	115
9312	240	0.25	17	6.3	105	95
9313	240	0.25	19	6.2	96	100
9314	240	0.25	19	6.1	103	100
9315	240	0.25	17	6.2	106	78
9316	240	0.25	20	6.0	94	175
9317	240	0.25	19	6.2	101	202
9318	240	0.25	18	6.2	104	147
9328	240	0	18	5.5	77	92
9329	240	0	17	5.9	84	114
9330	240	0	18	6.0	118	113
9331	240	0	18	6.3	100	111
9332	240	0	18	5.9	92	153
9333	240	0	19	6.1	86	95
9334	240	0	18	6.1	94	146

9264	270	1.5	15	6.4	102	94
9265	270	1.5	15	6.5	100	123
9266	270	1.5	16	6.4	97	100
9267	270	1.5	16	6.8	100	109
9268	270	1.5	22	6.0	88	76
9269	270	1.5	18	6.7	132	201
9270	270	1.5	16	6.5	97	109
9280	270	0.75	16	6.4	91	84
9281	270	0.75	17	6.7	106	102
9282	270	0.75	18	6.6	113	146
9283	270	0.75	17	6.5	121	188
9284	270	0.75	16	6.5	92	109
9285	270	0.75	18	6.7	106	115
9286	270	0.75	18	6.3	99	111
9296	270	0.5	16	6.3	96	114
9297	270	0.5	17	7.0	111	174
9298	270	0.5	18	6.7	108	106
9299	270	0.5	19	6.7	118	132
9300	270	0.5	19	6.9	118	96
9301	270	0.5	19	6.8	100	89
9302	270	0.5	18	6.7	100	87
9312	270	0.25	18	6.4	87	114
9313	270	0.25	18	6.5	90	156
9314	270	0.25	18	6.6	86	161
9315	270	0.25	18	6.3	93	144
9316	270	0.25	18	6.3	85	219
9317	270	0.25	19	6.5	96	200
9318	270	0.25	17	6.7	109	141
9328	270	0	16	6.5	82	126
9329	270	0	16	6.6	84	102
9330	270	0	17	6.7	92	179
9331	270	0	19	6.8	91	186
9332	270	0	17	6.4	84	152
9333	270	0	17	6.5	81	106
9334	270	0	15	6.4	92	112

Minimal underlying data set for Fig. 3

Perivascular cardiac collagen content in hearts 270 days after sham and whole body irradiation of WAG/RijCmcr rats with sequentially delivered 3-ion (protons+28Si+56Fe) beams.

Group	Slide ID#	perivascular collagen content (% of lumenal earea)
Sham	9220	207
Sham	9158	155
Sham	9335	343
Sham	9336	384
Sham	9337	328
Sham	9338	388
0.25 Gy	9312	246
0.25 Gy	9313	277
0.25 Gy	9314	230
0.25 Gy	9315	254
0.25 Gy	9316	180
0.25 Gy	9317	251
0.50 Gy	9296	150
0.50 Gy	9297	216
0.50 Gy	9298	329
0.50 Gy	9299	305
0.50 Gy	9300	266
0.50 Gy	9301	291
0.75 Gy	9280	277
0.75 Gy	9281	491
0.75 Gy	9282	498
0.75 Gy	9283	570
0.75 Gy	9284	472
0.75 Gy	9285	256
1.5 Gy	9270	790
1.5 Gy	9271	765
1.5 Gy	9272	480
1.5 Gy	9264	790
1.5 Gy	9265	536
1.5 Gy	9266	655

Minimal underlying data set for Fig. 4

Systemic blood pressure 270 days after whole body exposure of WAG/RijCmcr rats to sequentially delivered 3-ion mixed particles (protons+28Si+56Fe).

Date	Group	Specimen	Systolic	Mean	Diastolic
2/17/2018	1.5Gy	9264	135.9	98.0	78.6
2/21/2018	1.5Gy	9264	130.1	90.0	69.0
2/26/2018	1.5Gy	9264	119.7	83.2	65.0
2/17/2018	1.5Gy	9265	134.3	111.0	99.3
2/23/2018	1.5Gy	9265	117.7	78.3	58.7
2/26/2018	1.5Gy	9265	115.8	81.8	64.8
2/20/2018	1.5Gy	9266	133.4	99.5	82.6
2/21/2018	1.5Gy	9266	126.0	90.3	72.4
2/24/2018	1.5Gy	9266	125.7	77.3	53.1
2/26/2018	1.5Gy	9266	121.8	73.5	49.4
2/20/2018	1.5Gy	9267	134.0	95.8	76.8
2/21/2018	1.5Gy	9267	126.1	76.2	51.8
2/24/2018	1.5Gy	9267	124.3	82.9	60.5
2/26/2018	1.5Gy	9267	127.0	87.3	67.4
2/17/2018	1.5Gy	9268	132.3	100.4	84.5
2/20/2018	1.5Gy	9268	138.9	94.5	71.3
2/23/2018	1.5Gy	9268	133.9	105.7	91.6
2/28/2018	1.5Gy	9268	133.3	109.5	94.5
2/28/2018	1.5Gy	9268	119.2	83.7	66.0
2/17/2018	1.5Gy	9269	128.0	104.7	93.1
2/20/2018	1.5Gy	9269	128.3	78.5	53.6
2/23/2018	1.5Gy	9269	134.4	100.9	84.1
2/24/2018	1.5Gy	9269	123.1	93.8	78.1
2/27/2018	1.5Gy	9269	134.9	84.1	59.3
2/16/2018	0.75Gy	9280	113.4	84.5	67.6
2/17/2018	0.75Gy	9280	111.3	70.3	49.9
2/24/2018	0.75Gy	9280	127.3	83.6	61.7
2/27/2018	0.75Gy	9280	121.5	96.3	83.7
2/27/2018	0.75Gy	9280	114.1	75.2	54.4
2/23/2018	0.75Gy	9281	135.7	94.7	73.0
2/26/2018	0.75Gy	9281	114.8	74.3	54.0
2/28/2018	0.75Gy	9281	100.6	69.9	54.0
2/28/2018	0.75Gy	9281	113.3	82.1	66.4
2/16/2018	0.75Gy	9282	142.5	102.6	82.6

2/20/2018	0.75Gy	9282	125.5	102.2	90.5
2/21/2018	0.75Gy	9282	121.6	86.2	68.9
2/24/2018	0.75Gy	9282	134.7	107.1	93.3
2/16/2018	0.75Gy	9283	135.4	102.0	85.3
2/21/2018	0.75Gy	9283	110.0	92.2	75.0
2/24/2018	0.75Gy	9283	128.9	88.5	68.3
2/27/2018	0.75Gy	9283	107.9	64.1	42.2
2/28/2018	0.75Gy	9283	118.1	77.4	57.0
2/23/2018	0.75Gy	9284	129.2	104.0	91.4
2/24/2018	0.75Gy	9284	138.7	106.6	89.2
2/26/2018	0.75Gy	9284	119.0	92.8	79.6
2/20/2018	0.75Gy	9285	122.1	94.3	80.4
2/23/2018	0.75Gy	9285	125.3	88.8	70.6
2/24/2018	0.75Gy	9285	129.1	100.0	84.8
2/21/2018	0.5Gy	9296	123.0	96.6	83.4
2/24/2018	0.5Gy	9296	127.6	94.9	78.6
2/27/2018	0.5Gy	9296	115.2	79.5	61.6
2/28/2018	0.5Gy	9296	111.9	67.6	43.6
2/16/2018	0.5Gy	9297	125.8	93.8	77.8
2/17/2018	0.5Gy	9297	146.1	102.8	81.1
2/21/2018	0.5Gy	9297	128.7	94.7	77.7
2/24/2018	0.5Gy	9297	139.7	103.1	84.8
2/27/2018	0.5Gy	9297	126.8	89.5	70.8
2/16/2018	0.5Gy	9298	118.8	94.0	81.6
2/20/2018	0.5Gy	9298	134.1	106.6	92.9
2/23/2018	0.5Gy	9298	119.4	67.2	46.0
2/26/2018	0.5Gy	9298	122.4	99.1	87.4
2/28/2018	0.5Gy	9298	120.2	87.3	70.5
2/20/2018	0.5Gy	9299	122.6	96.3	83.1
2/23/2018	0.5Gy	9299	124.9	88.1	69.8
2/24/2018	0.5Gy	9299	131.6	92.4	72.9
2/28/2018	0.5Gy	9299	103.0	71.0	54.1
2/17/2018	0.5Gy	9300	128.3	100.6	86.8
2/20/2018	0.5Gy	9300	130.5	95.9	78.6
2/17/2018	0.5Gy	9301	126.3	91.6	74.3
2/20/2018	0.5Gy	9301	132.3	97.2	79.7
2/23/2018	0.5Gy	9301	115.8	89.1	75.8
2/27/2018	0.5Gy	9301	119.2	81.7	62.0

2/17/2018	0.25Gy	9312	123.2	86.1	67.5
2/21/2018	0.25Gy	9312	126.9	105.1	94.3
2/28/2018	0.25Gy	9312	110.1	75.0	57.1
2/17/2018	0.25Gy	9313	130.9	101.4	86.7
2/21/2018	0.25Gy	9313	119.7	85.4	68.3
2/23/2018	0.25Gy	9313	119.3	84.1	66.6
2/26/2018	0.25Gy	9313	114.7	84.9	70.0
2/26/2018	0.25Gy	9313	114.5	75.4	55.3
2/16/2018	0.25Gy	9314	125.1	110.2	102.8
2/20/2018	0.25Gy	9314	116.3	85.3	69.8
2/21/2018	0.25Gy	9314	122.9	94.7	80.6
2/27/2018	0.25Gy	9314	129.5	102.7	89.3
2/16/2018	0.25Gy	9315	121.0	82.0	62.5
2/20/2018	0.25Gy	9315	117.9	84.6	68.0
2/21/2018	0.25Gy	9315	121.4	82.1	62.3
2/24/2018	0.25Gy	9315	123.9	103.3	93.1
2/27/2018	0.25Gy	9315	107.9	65.7	45.0
2/24/2018	0.25Gy	9316	137.7	116.7	106.1
2/28/2018	0.25Gy	9316	122.2	87.5	70.2
2/20/2018	0.25Gy	9317	119.8	90.0	75.1
2/23/2018	0.25Gy	9317	121.4	89.4	74.1
2/24/2018	0.25Gy	9317	119.8	93.5	80.3
2/28/2018	0.25Gy	9317	102.3	82.1	71.0
2/23/2018	Sham	9329	108.5	87.9	77.7
2/28/2018	Sham	9329	136.0	98.6	79.9
2/16/2018	Sham	9330	118.2	87.7	72.2
2/17/2018	Sham	9330	119.4	89.0	72.6
2/21/2018	Sham	9330	118.5	87.6	72.1
2/21/2018	Sham	9330	113.7	81.7	66.2
2/27/2018	Sham	9330	116.1	84.7	67.6
2/17/2018	Sham	9331	130.9	97.2	80.4
2/21/2018	Sham	9331	121.1	83.1	64.6
2/21/2018	Sham	9331	119.1	80.7	61.5
2/23/2018	Sham	9331	125.0	94.0	78.3
2/27/2018	Sham	9331	123.4	82.7	61.7
2/20/2018	Sham	9332	135.0	104.9	89.9
2/21/2018	Sham	9332	138.5	103.8	86.5

2/24/2018	Sham	9332	119.1	76.6	55.0
2/26/2018	Sham	9332	115.6	82.1	67.8
2/21/2018	Sham	9333	121.8	84.2	61.8
2/21/2018	Sham	9333	97.8	57.7	37.7
2/26/2018	Sham	9333	110.3	71.2	51.7
2/17/2018	Sham	9334	135.7	100.4	82.8
2/23/2018	Sham	9334	118.5	85.3	68.7
2/24/2018	Sham	9334	129.5	89.4	69.4
2/28/2018	Sham	9334	117.5	76.7	56.3

Minimal underlying data set for Fig. 5

Levels of cytokines (pg/ml) present in the circulation 30 and 60 day after whole body exposure male WAG/RijCmcr rat to sequentially delivered 3-ion (protons+28Si+56Fe) beams.

Group/Dose (Gy)	Sample ID	Leptin	RANTES	LIX	IL-6	MCP-1	IL-12(p70)	GM-CSF	MIP-2	IP-10	IL-2	IL-1B	IL-18	IL-5	IL-1a	IL-10	G-CSF	Fractalkine	IL-4	TNFa	IL-13	IL-17A	GRO/KC	Eotaxin	MIP-1a	VEGF	EGF
Sham 30d (2)	S1	11309	4616	2742	2255	1279	744	685	278	272	173	183	180	147	113	148	133	69	55	38	45	34	26	19	20	6	OOR
Sham 30d (2)	S2	12477	7714	2452	2565	1173	473	185	287	243	127	87	93	125	187	74	43	60	51	30	29	20	OOR	18	19	6	2
Sham 30d (2)	S3	17434	4108	2954	1312	1359	662	100	275	329	104	79	93	123	94	99	31	33	49	33	25	19	OOR <	19	13	OOR <	OOR
Sham 30d (2)	S4	8644	4112	2529	1267	1180	812	106	240	287	104	286	111	101	70	159	51	54	44	27	16	32	OOR <	21	15	OOR <	OOR
Sham 30d (2)	S5	7198	3531	2599	2255	1300	765	502	278	205	240	94	169	147	156	56	66	63	53	35	37	32	OOR	19	21	8	OOR
0.25Gy 30d	S6	11204	3818	2939	1628	1245	812	114	257	300	212	150	430	170	106	122	62	64	61	32	25	44	OOR	23	25	19	0
0.25Gy 30d	S7	13113	3913	3100	1853	1158	571	762	329	306	199	113	217	146	115	81	60	78	68	30	30	26	OOR <	21	19	9	0
0.25Gy 30d	S8	10044	3751	3245	3351	1694	819	205	293	284	302	144	407	114	194	117	144	70	76	55	68	59	OOR	25	21	8	1
0.25Gy 30d	S9	11372	3677	2667	949	1510	588	108	219	254	245	125	297	158	63	112	32	60	81	51	56	40	OOR	23	19	OOR	0
0.25Gy 30d	S10	10695	2994	2904	1312	1552	765	221	191	239	154	121	75	174	139	109	75	26	80	66	52	69	OOR <	26	12	OOR <	OOR
0.25Gy 30d	S11	14740	3754	2980	1447	1430	967	114	253	260	112	239	151	166	120	151	61	34	82	50	33	52	OOR	24	14	OOR <	0
0.25Gy 30d	S12	11100	4291	3111	1853	1563	1103	106	326	253	245	144	294	230	187	151	87	74	77	59	56	47	OOR <	27	20	OOR	1
0.25Gy 30d	S13	5397	2517	2691	1040	1051	876	120	247	211	215	88	211	138	94	83	15	60	50	30	22	39	OOR <	21	18	1	0
0.25Gy 30d	S14	5158	3887	2046	2032	1353	758	108	263	327	220	105	195	151	139	96	72	64	64	44	55	39	OOR	23	21	1	0
0.25Gy 30d	S15	13410	4311	3103	1357	1461	638	93	296	247	191	113	307	161	115	140	46	64	70	61	40	57	OOR	29	21	OOR	0
0.25Gy 30d	S16	9890	5599	3194	2210	1504	1036	243	354	239	287	112	449	170	156	152	66	100	104	60	63	60	5	32	23	9	1
0.25Gy 30d	S17	8616	3051	3180	3004	1869	1220	174	281	226	257	179	288	201	120	138	25	67	85	58	49	78	OOR	29	18	1	0
0.5Gy 30d	S18	6720	3761	2757	1357	1313	670	133	260	222	171	87	151	117	98	69	25	49	44	26	16	16	OOR <	24	17	OOR	0
0.5Gy 30d	S19	8459	3882	2892	1583	1699	944	103	230	280	201	188	229	151	181	103	62	46	72	60	47	29	OOR <	25	15	OOR	0
0.5Gy 30d	S20	6516	5055	2916	2032	1604	876	133	287	298	220	94	348	161	165	80	51	68	52	49	47	40	OOR	26	20	8	0
0.5Gy 30d	S21	7523	2508	2843	2299	1644	889	174	247	287	293	144	245	168	124	87	53	68	81	58	35	64	OOR	28	20	5	1
0.5Gy 30d	S22	13793	3260	2420	541	934	758	148	203	282	159	91	73	123	65	74	18	63	50	23	25	18	OOR	12	19	5	4
0.5Gy 30d	S23	9118	2959	2578	2299	1151	812	585	318	240	248	89	261	149	111	76	38	68	66	28	34	30	OOR <	23	21	4	0
0.5Gy 30d	S24	8236	3864	2811	2344	1417	646	243	253	241	169	95	154	133	106	79	50	49	43	34	21	24	OOR	23	18	OOR	0
0.5Gy 30d	S25	10479	2921	3400	2829	1632	956	191	318	262	240	102	327	167	194	179	154	51	79	52	29	45	OOR	30	16	OOR <	OOR
0.5Gy 30d	S26	22643	3662	2784	1040	985	662	108	260	272	112	194	138	136	56	171	11	21	55	28	16	12	OOR	17	15	OOR <	OOR
0.5Gy 30d	S27	6170	3874	2578	2077	1411	838	123	269	228	207	112	252	146	96	49	29	69	66	51	63	33	OOR <	21	22	4	0
0.5Gy 30d	S28	12247	3699	2762	1402	1151	700	74	243	299	171	104	98	143	78	91	31	47	45	34	38	34	OOR	22	15	OOR <	0
0.5Gy 30d	S29	9814	4129	2785	1763	1259	956	123	272	242	234	117	255	166	153	73	49	62	85	47	44	26	OOR	25	16	OOR	0
150cGy 30d	S30	11532	4514	3004	1402	1492	901	123	302	313	391	123	341	167	307	138	53	75	77	60	57	47	OOR	27	22	4	0
150cGy 30d	S31	10051	2928	2495	2388	1473	832	120	281	284	251	113	169	180	273	85	36	67	57	49	33	28	OOR <	23	20	OOR	0
150cGy 30d	S32	16085	3799	2986	1357	1180	870	140	266	295	166	117	169	135	191	48	31	51	61	28	35	26	OOR	22	19	OOR	0
150cGy 30d	S33	8237	4155	2412	1357	1173	597	126	269	291	147	76	140	147	82	70	18	55	54	27	18	29	OOR <	21	18	1	OOR
150cGy 30d	S34	15256	4540	3032	2477	1649	1075	655	253	301	284	147	258	171	142	104	61	55	85	66	61	63	OOR <	27	19	OOR <	0
150cGy 30d	S35	8463	2594	2519	1942	1498	920	198	260	258	311	190	348	188	198	125	82	73	88	41	38	72	OOR <	28	22	OOR	0

150cGy 30d	S36	16149	3478	3138	4161	1516	1002	280	318	321	251	134	321	158	234	125	54	81	81	61	49	43	OOR	30	23	4	0
150cGy 30d	S37	8009	4204	2692	2388	1259	1199	174	339	284	223	108	258	153	191	92	39	59	55	47	19	19	OOR <	21	19	OOR	OOR
150cGy 30d	S38	13869	4607	2430	2388	1552	991	152	284	293	323	160	324	177	279	125	80	80	96	62	79	62	OOR	33	23	OOR	1
150cGy 30d	S39	13985	2866	2538	1628	1166	765	191	250	281	209	115	248	156	98	105	60	66	68	49	24	43	OOR <	23	20	OOR	0
150cGy 30d	S40	9020	3171	2246	904	1035	504	93	211	207	129	63	157	126	78	33	45	42	48	23	16	26	OOR <	20	16	OOR <	OOR
150cGy 30d	S41	17128	2461	1365	2299	1231	562	140	302	261	237	116	245	133	139	106	64	65	61	38	39	24	OOR	26	20	OOR	6
Sham 30d (1)	Sham 1	11442	4520	2869	404	1368	724	12	150	278	127	125	160	135	45	58	25	60	45	33	12	18	#DIV/0!	18	20	5	0
Sham 30d (1)	Sham 2	12508	5861	2620	768	924	440	40	101	264	133	77	146	113	37	54	7	65	35	31		20	#DIV/0!	12	21	6	2
Sham 30d (1)	Sham 3	14461	3732	2911	41	1019	672	51	126	319	46	54	34	101	45	36	11	26	20	19		9	#DIV/0!	13	11		
Sham 30d (1)	Sham 4	7714	4003	2455	949	1058	685	33	138	290	108	276	53	98	47	98	16	31	31	25	16	17	#DIV/0!	17	14		
Sham 30d (1)	Sham 5	8890	3723	2580	132	1009	351	24	55	254	171	71	143	130	44	35	15	65	30	36		23	#DIV/0!	16	21	1	0
75 cGy 30d	0.75Gy - 1	9091	3761	2931	949	1683	777	57	152	309	144	99	78	122	56	70	34	29	54	43	19	45	#DIV/0!	21	12		0
75 cGy 30d	0.75Gy - 2	8180	3492	3081	631	1698	766	28	79	369	181	118	109	137	75	70	45	38	47	51	37	38	#DIV/0!	22	14		0
75 cGy 30d	0.75Gy - 3	8604	3962	3320	904	1615	783	30	85	361	229	113	205	161	52	48	22	55	45	50	18	27	#DIV/0!	19	17		0
75 cGy 30d	0.75Gy - 4	11718	4382	3690	495	1492	798	28	138	336	389	215	475	183	86	172	74	94	61	59	56	75	#DIV/0!	23	25	66	2
75 cGy 30d	0.75Gy - 5	10855	3777	3051	223	1473	776	8	127	315	157	84	153	150	51	60	35	48	46	35	13	21	#DIV/0!	11	18		

PERCENT

Sham 30d (2) vs.	0.25Gy 30d	-9	-21	10	-5	15	22	-37	1	-2	47	-7	115	28	4	13	-4	14	49	53	50	85	-79	31	9	7	-84
Sham 30d (2) vs.	0.5Gy 30d	-11	-25	5	-7	7	17	-44	-3	-2	35	-19	64	14	-4	-12	-27	-1	22	25	14	12	#DIV/0!	19	1	-21	-70
Sham 30d (1) vs.	0.75Gy 30d	-12	-11	20	40	48	36	-5	2	20	88	4	90	30	47	49	186	7	57	65	104	137	#DIV/0!	24	1	1538	-20
Sham 30d (2) vs.	150cGy 30d	8	-25	-3	7	7	23	-37	2	6	63	-16	92	23	49	-10	-20	15	38	41	28	46	#DIV/0!	30	13	-50	-60

Group/Dose (Gy)	Sample ID	Leptin	RANTES	LIX	IL-6	MCP-1	IL-12(p70)	GM-CSF	MIP-2	IP-10	IL-2	IL-1B	IL-18	IL-5	IL-1a	IL-10	G-CSF	Fractalkine	IL-4	TNFa	IL-13	IL-17A	GRO/KC	Eotaxin	MIP-1a	VEGF	EGF
Sham 60d (2)	S42	13238	3778	2447	2477	1188	638	133	299	242	157	78	195	129	106	51	12	51	55	30	33	22	OOR <	21	17	1	OOR
Sham 60d (2)	S43	10136	4053	2226	1898	1320	751	108	247	216	157	108	229	165	174	106	53	55	63	48	18	26	OOR <	23	19	OOR	0
Sham 60d (2)	S44	26894	3897	2579	2121	1035	812	70	233	290	84	72	80	122	96	76	13	25	39	15	35	22	OOR	23	13	OOR <	OOR
Sham 60d (2)	S45	6521	3071	2093	2210	1353	819	174	331	232	287	123	278	144	198	87	62	62	76	51	65	63	OOR	25	17	OOR	0
Sham 60d (2)	S46	8237	3515	2650	2388	1398	926	198	339	236	251	97	217	139	171	139	58	60	76	43	61	54	47	25	22	1	0
0.25Gy 60d	S48	15003	3661	3107	1583	1180	851	114	243	256	196	92	180	120	127	74	62	56	66	41	49	22	OOR <	21	20	OOR	OOR
0.25Gy 60d	S49	10228	3164	3038	2255	1333	708	169	339	277	171	120	261	127	156	109	72	57	59	47	49	38	OOR	24	20	1	0
0.25Gy 60d	S50	12282	3175	2290	1221	985	416	85	226	220	104	59	146	105	102	44	3	26	36	25	15	9	OOR <	18	13	OOR <	OOR
0.25Gy 60d	S51	13903	3356	2980	949	1366	605	67	219	243	84	89	75	154	88	93	36	17	41	36	10	39	OOR <	19	8	OOR <	OOR <
0.25Gy 60d	S52	12048	3114	2728	1176	1043	654	85	237	274	46	363	63	138	58	336	5	17	38	27	13	10	OOR <	18	10	OOR <	OOR <
0.25Gy 60d	S53	9778	2840	2327	2166	1492	654	169	250	212	209	109	124	150	122	80	28	43	62	45	35	34	OOR <	23	16	OOR <	OOR
0.25Gy 60d	S54	8439	2872	2364	1673	1238	785	93	275	192	173	70	220	158	134	60	OOR	60	48	34	27	22	OOR <	23	19	OOR	0
0.25Gy 60d	S55	6301	2878	1526	858	1010	534	120	253	273	125	99	119	121	156	94	27	45	50	44	35	29	OOR <	19	17	OOR	OOR
0.25Gy 60d	S56	14380	3995	2637	2477	1655	765	111	260	252	263	118	261	150	100	100	52	79	52	45	32	34	OOR <	23	22	OOR	1
0.25Gy 60d	S57	9905	3907	2606	2032	1604	758	108	257	233	237	131	288	167	124	107	36	69	64	50	38	37	OOR	24	22	0	173
0.25Gy 60d	S58	10777	3106	2781	1402	1504	832	106	296	267	147	89	98	142	72	93	22	40	54	34	12	30	OOR <	23	17	OOR <	OOR

0.5Gy 60d	S59	6230	4611	2900	2121	1359	991	130	284	247	251	134	226	137	129	100	34	68	68	54	35	33	OOR <	27	21	1	0
0.5Gy 60d	S60	9954	3838	2613	1357	1279	729	88	222	223	118	158	113	142	56	78	13	33	48	43	27	24	OOR <	20	14	OOR <	OOR
0.5Gy 60d	S61	8394	3811	2559	1853	1121	758	140	278	224	191	86	314	141	82	70	21	65	53	39	25	19	OOR <	20	21	OOR	0
0.5Gy 60d	S62	10651	2875	2812	1402	1694	758	108	253	247	351	140	397	157	90	91	51	68	60	67	28	60	OOR <	28	23	OOR	1
0.5Gy 60d	S63	9180	2551	2288	2565	1252	737	106	222	228	240	77	242	148	88	69	11	69	49	37	37	24	OOR <	20	19	OOR	OOR
0.5Gy 60d	S64	8487	2753	2316	1176	880	838	156	299	216	134	90	198	138	84	95	19	57	46	12	30	26	OOR <	18	18	OOR	3
0.5Gy 60d	S65	6880	3540	2502	1176	1300	662	133	233	219	104	78	113	128	90	56	20	55	56	39	14	15	OOR <	15	15	OOR <	OOR
0.5Gy 60d	S66	20026	3168	2583	1312	1180	534	95	321	253	145	171	98	137	90	100	23	22	48	37	28	8	OOR <	22	10	OOR <	OOR
0.5Gy 60d	S67	22342	3379	2812	3437	1486	1199	585	400	263	311	156	180	173	226	168	86	47	94	58	48	32	OOR	26	17	OOR <	0
0.5Gy 60d	S68	6369	4102	2601	858	1051	799	111	310	231	159	158	172	122	165	109	38	57	50	32	27	22	OOR <	23	19	1	OOR
0.5Gy 60d	S69	17374	3298	2584	2388	1621	729	126	349	255	257	145	261	158	129	89	43	60	96	50	42	58	OOR	22	20	OOR	1
0.5Gy 60d	S70	12142	3853	2925	1221	1158	870	156	284	267	176	126	223	163	115	116	32	53	66	36	48	30	OOR <	25	15	OOR <	13
150 cGy 60d	S71	10407	3462	1847	2299	1245	391	114	247	233	106	62	83	104	94	40	10	38	34	18	8	14	OOR <	19	14	OOR	OOR
150 cGy 60d	S72	14054	3320	2176	2121	1143	562	198	260	242	143	87	175	135	90	58	29	56	55	35	47	30	OOR <	21	21	OOR	0
150 cGy 60d	S73	18631	5667	2165	2255	1333	913	144	240	258	207	101	146	122	108	66	6	57	41	31	40	25	OOR <	25	18	OOR	OOR
150 cGy 60d	S74	11188	4736	1987	2121	951	287	144	329	218	112	76	58	123	104	50	32	35	55	24	25	15	OOR	21	15	OOR <	OOR
150 cGy 60d	S75	13623	4083	2566	1402	1209	377	111	250	229	129	82	140	130	82	62	16	52	52	38	26	20	OOR <	23	17	OOR <	OOR
150 cGy 60d	S76	20937	4842	2728	1673	977	440	221	250	298	86	96	175	104	56	65	3	40	33	21	8	13	OOR <	11	20	2	14
150 cGy 60d	S77	10021	3051	2197	1808	1018	693	130	272	229	199	86	217	160	113	53	31	56	45	32	43	17	OOR <	27	19	1	0
150 cGy 60d	S78	10625	4663	2426	1673	1082	597	111	287	229	186	84	175	144	168	45	25	49	45	23	16	19	OOR	23	17	OOR <	OOR
150 cGy 60d	S79	15078	5496	2369	1673	1353	462	108	272	246	171	91	98	129	90	42	6	52	35	31	32	16	OOR <	22	18	OOR	OOR
150 cGy 60d	S80	16143	3072	2335	1040	1173	440	90	203	243	147	82	186	144	120	64	21	69	46	35	29	35	OOR <	22	17	3	OOR
150 cGy 60d	S81	11887	4351	2789	1718	1610	901	180	253	197	186	111	233	158	106	120	62	75	76	47	47	23	OOR <	21	22	OOR	0
150 cGy 60d	S82	18736	3871	1329	1853	1300	857	169	284	250	287	140	314	161	98	100	37	68	76	51	41	57	OOR <	23	20	OOR	1
Sham 60d (1)	S1	28171	11518	4581	1266	1615	656	488	359	543	112	156	152	142	117	167	150	89	42	34	39	31	697	23	26	71	
Sham 60d (1)	S2	36240	9177	3972	1582	1518	470	522	396	511	143	142	154	123	125	172	166	88	52	35	43	68	784	27	23	11	0
Sham 60d (1)	S3	19997	9463	4851	2476	1313	603	360	329	409	127	185	124	124	199	133	166	63	79	22	36	36	710	21	23	10	0
Sham 60d (1)	S4	41246	9030	4638	2565	1531	315	294	415	523	181	221	196	125	174	194	69	88	57	38	43	26	794	27	26	66	0
Sham 60d (1)	S5	42909	12015	5086	1265	1441	579	1100	418	641	552	1344	685	132	111	1128	104	166	45	32	14	34	2092	22	41	154	5
Sham 60d (1)	S6	22482	12100	4853	3003	1467	654	408	378	502	154	116	153	129	150	120	184	82	65	36	41	46	871	24	25	90	0
Sham 60d (1)	S7	43772	9245	4035	2075	1461	388	161	351	452	181	142	135	127	161	143	145	82	59	25	31	24	632	22	23	37	0
75 cGy 60d	S8	13503	9529	4459	1853	1359	420	609	356	428	92	119	113	124	146	125	153	92	51	37	41	32	676	22	22	32	
75 cGy 60d	S9	31798	10793	4983	768	1230	175	124	346	457	83	197	125	119	98	205	121	83	52	14	31	31	631	13	28	75	0
75 cGy 60d	S10	19904	9730	3138	1357	1282	305	362	328	395	109	97	63	110	94	78	126	64	39	21	32	22	561	20	24	7	0
75 cGy 60d	S11	24979	8296	4606	904	1243	285	103	284	410	80	72	73	102	64	51	53	49	44	10	14	20	472	17	17	6	
75 cGy 60d	S12	14539	10258	4629	1221	1205	162	161	323	418	104	203	144	124	194	170	104	72	50	24	33	28	385	19	22	9	
75 cGy 60d	S13	14076	12754	4874	858	1142	291	135	373	436	78	256	78	111	106	240	104	61	42	12	10	23	501	16	26	49	1
75 cGy 60d	S14	16670	10800	4103	1673	1532	290	162	301	445	108	106	108	127	122	88	133	81	58	26	37	31	323	24	24	36	
75 cGy 60d	S15	25245	9139	4034	1762	1306	510	102	311	403	69	113	88	118	98	100	109	53	62	20	37	18		18	20	11	
75 cGy 60d	S16	21106	10706	4094	1312	1503	316	186	343	445	110	181	108	122	187	185	158	76	59	22	20	27	746	23	21	11	0
75 cGy 60d	S17	16074	10169	4362	1533	1448	405	120	345	448	100	80	96	106	125	88	131	57	33	31	33	28	486	18	19	9	
75 cGy 60d	S18	25679	10239	4774	2165	1272	410	198	405	480	132	251	262	139	115	240	118	82	63	28	24	30	41	23	21	7	0

75 cGy 60d	S19	29969	10285	4191	1176	1434	259	170	331	431	121	142	108	133	153	145	136	76	55	26	32	39	636	18	21	70	0

PERCENT

Sham 60d (2) vs.	0.25Gy 60d	-14	-10	8	-27	4	-13	-18	-10	1	-15	28	-17	0	-24	18	-14	-9	-16	4	-33	-26	#DIV/0!	-10	-6	-32	10595
Sham 60d (2) vs.	0.5Gy 60d	-12	-5	9	-22	2	1	18	-1	-2	9	33	6	4	-25	3	-18	8	-1	13	-24	-22	#DIV/0!	-5	0	-5	541
Sham 60d (1) vs.	0.75Gy 60d	-37	-1	-5	-32	-10	-39	-57	-11	-15	-52	-54	-50	-7	-16	-51	-14	-25	-11	-29	-18	-28	-47	-19	-17	-57	-67
Sham 60d (2) vs.	150 cGy 60d	10	15	-7	-19	-5	-27	5	-9	-2	-13	-4	-17	-4	-31	-31	-42	7	-20	-14	-29	-36	#DIV/0!	-9	2	71	660

NOTES:

OOR	Out of Range (cubic spline curve)
OOR >	Out of Range Above the 4 or 5 Parameter Logistic Standard Curve
OOR <	Out of Range Below the 4 or 5 Parameter Logistic Standard Curve

Minimal underlying data set for Fig. 6 and Fig. 7

Components of the immune system (CD3, CD20, CD56, CD68) present in the heart and kidney at 270 days after whole body irradiation of WAG/RijCmcr rats with 1.5 Gy of of sequentially delivered 3-ion (protons+28Si+56Fe) beams.

Slide ID	DAB area	Total tissue area	CD marker	Rat ID	Exp.	Group or Dose (Gy)	Tissue
9333H with CD3+ cells-1	25235	36010138	CD3	9333	BNL 3-ions	Sham	Heart
9333H with CD3+ cells-2	31007	37389674	CD3	9334	BNL 3-ions	Sham	Heart
9333H with CD3+ cells-3	46667	46051824	CD3	9335	BNL 3-ions	Sham	Heart
9270H with CD3+ cells-1	36367	59714359	CD3	9270	BNL 3-ions	1.5Gy	Heart
9270H with CD3+ cells-2	17597	32427950	CD3	9271	BNL 3-ions	1.5Gy	Heart
9270H with CD3+ cells-3	32696	46027288	CD3	9272	BNL 3-ions	1.5Gy	Heart
9273H with CD3+ cells-1	19089	48618443	CD3	9273	BNL 3-ions	1.5Gy	Heart
9273H with CD3+ cells-2	28617	39442366	CD3	9274	BNL 3-ions	1.5Gy	Heart
9273H with CD3+ cells-3	15250	43204230	CD3	9275	BNL 3-ions	1.5Gy	Heart
9272-9333-1b	78471	122874864	CD3	9333	BNL 3-ions	Sham	Kidney
9334-9335-1a	88948	113657400	CD3	9334	BNL 3-ions	Sham	Kidney
9334-9335-1b	108567	144536320	CD3	9335	BNL 3-ions	Sham	Kidney
9270-9271-1a	104311	147258960	CD3	9270	BNL 3-ions	1.5Gy	Kidney
9270-9271-1b	187341	135666592	CD3	9271	BNL 3-ions	1.5Gy	Kidney
9272-9333-1a	69030	129693264	CD3	9272	BNL 3-ions	1.5Gy	Kidney
9333 34 35hrt with CD56+ cells-a	19293	32039302	CD56	9333	BNL 3-ions	Sham	Heart
9333 34 35hrt with CD56+ cells-b	27054	25544174	CD56	9334	BNL 3-ions	Sham	Heart
9333 34 35hrt with CD56+ cells-c	49212	37336420	CD56	9335	BNL 3-ions	Sham	Heart
9336 37 38hrt with CD56+ cells-a	26713	39935648	CD56	9336	BNL 3-ions	Sham	Heart
9336 37 38hrt with CD56+ cells-b	36284	48539964	CD56	9337	BNL 3-ions	Sham	Heart
9336 37 38hrt with CD56+ cells-c	41864	53789024	CD56	9338	BNL 3-ions	Sham	Heart
9270 71 72hrt with CD56+ cells-a	58515	46697948	CD56	9270	BNL 3-ions	1.5Gy	Heart
9270 71 72hrt with CD56+ cells-b	41770	29782264	CD56	9271	BNL 3-ions	1.5Gy	Heart
9270 71 72hrt with CD56+ cells-c	38141	40285260	CD56	9272	BNL 3-ions	1.5Gy	Heart
9273 74 75hrt with CD56+ cells-a	46708	43241352	CD56	9273	BNL 3-ions	1.5Gy	Heart
9273 74 75hrt with CD56+ cells-b	45167	35460524	CD56	9274	BNL 3-ions	1.5Gy	Heart
9273 74 75hrt with CD56+ cells-c	36382	38988072	CD56	9275	BNL 3-ions	1.5Gy	Heart
9272-9333-2b	639254	122064928	CD56	9333	BNL 3-ions	Sham	Kidney
9334-9335-2a	358133	111235808	CD56	9334	BNL 3-ions	Sham	Kidney
9334-9335-2b	690243	141351504	CD56	9335	BNL 3-ions	Sham	Kidney
9270-9271-2a	905164	159126560	CD56	9270	BNL 3-ions	1.5Gy	Kidney
9270-9271-2b	981354	134451408	CD56	9271	BNL 3-ions	1.5Gy	Kidney
9272-9333-2a	398560	123169056	CD56	9272	BNL 3-ions	1.5Gy	Kidney
9336-9337-9338-3a	5635	33194716	CD68	9336	BNL 3-ions	Sham	Heart

9336-9337-9338-3b	15599	56556804	CD68	9337	BNL 3-ions	Sham	Heart
9336-9337-9338-3c	12340	59028664	CD68	9338	BNL 3-ions	Sham	Heart
9273-9274-9275-3a	22309	49364512	CD68	8273	BNL 3-ions	1.5Gy	Heart
9273-9274-9275-3b	21063	40177340	CD68	9274	BNL 3-ions	1.5Gy	Heart
9273-9274-9275-3c	24154	44021384	CD68	9275	BNL 3-ions	1.5Gy	Heart
9272-9333-3b	247881	121025456	CD68	9333	BNL 3-ions	Sham	Kidney
9334-9335-3a	237883	107807592	CD68	9334	BNL 3-ions	Sham	Kidney
9334-9335-3b	296256	140794848	CD68	9335	BNL 3-ions	Sham	Kidney
9270-9271-3a	419438	152527072	CD68	9270	BNL 3-ions	1.5Gy	Kidney
9270-9271-3b	354772	132457200	CD68	9271	BNL 3-ions	1.5Gy	Kidney
9272-9333-3a	285840	122011112	CD68	9272	BNL 3-ions	1.5Gy	Kidney
9270 Hrt CD20	14716	60096724	CD20	9270	BNL 3-ions	Sham	Heart
9271 Hrt CD20	4995	32412108	CD20	9271	BNL 3-ions	Sham	Heart
9272 Hrt CD20	3774	45625080	CD20	9272	BNL 3-ions	Sham	Heart
9333 Hrt CD20	4149	38304684	CD20	9333	BNL 3-ions	1.5 Gy	Heart
9334 Hrt CD20	4417	43825368	CD20	9334	BNL 3-ions	1.5 Gy	Heart
9335 Hrt CD20	4136	47298620	CD20	9335	BNL 3-ions	1.5 Gy	Heart
9270 Kd CD20	19851	155797760	CD20	9270	BNL 3-ions	Sham	Kidney
9271 Kd CD20	21667	135534000	CD20	9271	BNL 3-ions	Sham	Kidney
9272 Kd CD20	8495	125318416	CD20	9272	BNL 3-ions	Sham	Kidney
9333 Kd CD20	6683	121052248	CD20	9333	BNL 3-ions	1.5 Gy	Kidney
9334 Kd CD20	16678	109237440	CD20	9334	BNL 3-ions	1.5 Gy	Kidney
9335 Kd CD20	11736	139414224	CD20	9335	BNL 3-ions	1.5 Gy	Kidney

Minimal underlying data set for Fig. S2

Table. Risk factors for cardiac (total cholesterol -Chol, triglycerides - TRIGS) and kidney (blood urea nitrogen - BUN, total protein - TP) injury in the serum of male WAG/RijCmcr rats after whole body exposure with proton ions.

Rat #	Blood collection (Days post proton exposure)	Total protons Dose (Gy)	BUN mg/dL	TP (g/dl)	Chol (mg/dl)	TRIGS (mg/dl)
9156	30	0.00	21	6.2	94	207
9157	30	0.00	19	6.6	77	114
9158	30	0.00	20	6.2	70	134
9159	30	0.00	19	6.8	89	137
9160	30	0.00	17	6.5	78	91
9161	30	0.00	19	6.4	62	68
9144	30	0.25	20	6.5	83	99
9145	30	0.25	19	6.4	74	92
9146	30	0.25	18	6.4	75	105
9147	30	0.25	20	6.4	83	136
9148	30	0.25	18	6.3	70	75
9149	30	0.25	20	6.8	88	84
9132	30	0.50	19	6.4	60	65
9133	30	0.50	19	6.5	86	116
9134	30	0.50	18	6.4	83	104
9135	30	0.50	19	6.6	80	170
9136	30	0.50	18	6.4	78	154
9137	30	0.50	18	6.5	75	102
9120	30	1.00				
9121	30	1.00	18	6.8	83	157
9122	30	1.00	18	6.2	81	134
9123	30	1.00	16	6.3	79	147
9124	30	1.00	17	6.5	86	106
9125	30	1.00	18	6.4	80	178
9108	30	1.50	19	6.1	77	120
9109	30	1.50	18	6.0	67	98
9110	30	1.50	22	6.3	74	96
9111	30	1.50	18	6.6	81	160
9112	30	1.50	19	6.1	69	83
9113	30	1.50	17	6.4	87	120
9156	60	0.00	19	6.7	119	259
9157	60	0.00	20	6.8	86	108
9158	60	0.00	21	6.9	95	157
9159	60	0.00	21	6.8	108	161
9160	60	0.00	18	6.4	75	88
9161	60	0.00	17	6.6	85	96
9144	60	0.25	19	6.4	92	124
9145	60	0.25	18	6.6	84	108
9146	60	0.25	17	6.4	87	96
9147	60	0.25	19	6.7	103	148

9148	60	0.25	20	6.1	81	70
9149	60	0.25	20	6.7	106	143
9132	60	0.50	19	6.2	71	40
9133	60	0.50	17	6.6	90	122
9134	60	0.50	19	6.6	100	102
9135	60	0.50	19	6.3	88	133
9136	60	0.50	18	6.5	93	146
9137	60	0.50	19	6.6	72	74
9120	60	1.00	18	6.2	85	130
9121	60	1.00	18	6.8	98	156
9122	60	1.00	17	6.3	93	161
9123	60	1.00	17	6.4	106	141
9124	60	1.00	18	6.8	102	161
9125	60	1.00	19	7.0	96	205
9108	60	1.50				
9109	60	1.50	17	6.7	86	105
9110	60	1.50	19	6.5	107	159
9111	60	1.50	19	6.4	79	116
9112	60	1.50	21	6.1	92	107
9113	60	1.50	19	6.6	98	152
9115	60	1.50	19	6.1	70	112
9156	90	0.00	18	6.6	103	291
9157	90	0.00	17	7.0	90	259
9158	90	0.00	18	7.0	82	136
9159	90	0.00	20	6.8	105	138
9160	90	0.00	20	6.6	75	118
9161	90	0.00	18	7.2	86	129
9144	90	0.25	20	6.9	92	157
9145	90	0.25	19	6.6	85	131
9146	90	0.25	20	7.1	75	123
9147	90	0.25	22	7.2	95	130
9148	90	0.25	19	6.7	75	108
9149	90	0.25	18	6.8	95	130
9132	90	0.50				
9133	90	0.50	18	6.3	84	144
9134	90	0.50	19	7.0	97	107
9135	90	0.50	17	7.0	94	138
9136	90	0.50	16	6.6	97	114
9137	90	0.50	16	6.7	77	89
9138	90	0.50	18	7.0	81	125
9120	90	1.00	19	6.7	83	132
9121	90	1.00	20	6.9	93	152
9122	90	1.00	18	6.5	82	172
9123	90	1.00	17	6.9	91	187
9124	90	1.00	20	6.7	94	159

9125	90	1.00	21	6.8	87	243
9108	90	1.50	20	6.7	94	143
9109	90	1.50	23	6.6	89	110
9110	90	1.50	25	6.0	80	84
9111	90	1.50	21	6.9	97	125
9112	90	1.50	22	6.5	97	121
9113	90	1.50	19	6.7	106	133
9156	120	0.00	18	6.8	113	183
9157	120	0.00	18	6.6	90	174
9158	120	0.00	16	6.3	88	93
9159	120	0.00	18	6.6	112	148
9160	120	0.00	15	6.4	83	108
9161	120	0.00	16	6.4	90	107
9144	120	0.25	19	6.3	99	140
9145	120	0.25	17	6.3	89	104
9146	120	0.25	16	6.2	86	118
9147	120	0.25	19	6.6	112	189
9148	120	0.25	19	6.1	80	72
9149	120	0.25	19	6.6	128	153
9132	120	0.50	19	6.1	82	94
9133	120	0.50	17	6.4	99	177
9134	120	0.50	19	6.4	113	135
9135	120	0.50	16	6.7	98	181
9136	120	0.50	17	6.3	110	96
9137	120	0.50	15	6.0	87	120
9120	120	1.00	16	6.2	79	146
9121	120	1.00	18	6.8	105	139
9122	120	1.00	17	6.3	96	204
9123	120	1.00	16	6.4	100	163
9124	120	1.00	18	6.5	114	115
9108	120	1.50	20	6.7	107	194
9109	120	1.50	18	6.1	83	98
9110	120	1.50	20	6.3	93	187
9111	120	1.50	19	6.5	110	181
9112	120	1.50	17	6.4	103	130
9113	120	1.50	17	6.2	99	116
9156	150	0.00	20	6.7	120	258
9157	150	0.00	20	6.6	100	189
9158	150	0.00	17	6.4	94	185
9159	150	0.00				
9160	150	0.00	17	6.3	88	117
9161	150	0.00	16	6.5	95	183
9162	150	0.00	18	6.8	97	153
9144	150	0.25	19	6.5	112	159

9145	150	0.25	17	6.8	97	154
9146	150	0.25	17	6.4	101	125
9147	150	0.25	17	6.7	110	192
9148	150	0.25	16	6.4	83	121
9149	150	0.25	19	6.8	121	164
9132	150	0.50	18	6.0	46	80
9133	150	0.50	18	6.8	110	199
9134	150	0.50	18	6.5	114	167
9135	150	0.50	19	6.6	100	173
9136	150	0.50	16	6.5	114	193
9137	150	0.50	18	6.9	100	157
9120	150	1.00	18	6.6	88	157
9121	150	1.00	18	6.8	102	131
9122	150	1.00	17	6.5	104	207
9123	150	1.00	14	6.8	111	138
9124	150	1.00	18	6.6	112	163
9125	150	1.00	17	6.8	103	238
9108	150	1.50				
9109	150	1.50	18	7.1	121	183
9110	150	1.50	19	6.4	97	174
9111	150	1.50	18	6.7	118	165
9112	150	1.50	17	6.2	108	234
9113	150	1.50	21	6.9	107	122
9114	150	1.50	19	6.3	84	134
9156	180	0.00	24	6.5	120	218
9157	180	0.00	24	6.8	106	117
9158	180	0.00	18	6.2	91	174
9159	180	0.00	19	6.3	113	182
9160	180	0.00	21	6.6	95	155
9161	180	0.00	20	6.6	97	259
9144	180	0.25	19	6.3	105	110
9145	180	0.25	18	6.5	93	162
9146	180	0.25	17	6.4	89	130
9147	180	0.25	18	6.9	120	255
9148	180	0.25	16	6.1	90	156
9149	180	0.25	21	6.8	128	162
9132	180	0.50	19	6.4	83	134
9133	180	0.50	20	6.6	105	257
9134	180	0.50	17	6.3	104	175
9135	180	0.50	18	6.7	82	215
9136	180	0.50	17	6.9	109	163
9137	180	0.50	18	6.9	99	137
9120	180	1.00	19	6.4	86	126
9121	180	1.00	18	6.5	105	155
9122	180	1.00				

9123	180	1.00	18	6.7	92	143
9124	180	1.00	18	6.6	111	143
9125	180	1.00	18	6.5	94	157
9126	180	1.00	17	6.1	89	163
9108	180	1.50				
9109	180	1.50	23	7.3	121	209
9110	180	1.50	20	6.8	98	137
9111	180	1.50	20	7.0	117	183
9112	180	1.50	18	6.8	115	147
9113	180	1.50	19	6.8	113	188
9114	180	1.50				
9115	180	1.50	18	6.2	85	130
9156	210	0.00	20	6.1	113	212
9157	210	0.00	17	6.5	101	187
9158	210	0.00	17	6.3	90	159
9159	210	0.00	21	6.5	120	128
9160	210	0.00	20	6.1	101	129
9161	210	0.00	17	6.4	103	127
9144	210	0.25	19	6.7	115	224
9145	210	0.25	18	6.7	97	117
9146	210	0.25	18	6.3	102	163
9147	210	0.25				
9148	210	0.25	18	6.1	91	106
9149	210	0.25	20	6.6	131	150
9150	210	0.25	18	6.3	107	152
9132	210	0.50	19	6.2	91	78
9133	210	0.50	17	6.5	109	174
9134	210	0.50	17	6.2	110	173
9135	210	0.50	17	5.8	52	51
9136	210	0.50	17	6.2	110	193
9137	210	0.50	18	6.5	100	133
9120	210	1.00	17	6.0	75	125
9121	210	1.00	17	6.5	99	169
9122	210	1.00	17	6.1	107	246
9123	210	1.00	16	6.4	104	129
9124	210	1.00	17	6.1	110	183
9125	210	1.00	18	6.8	96	121
9108	210	1.50	18	6.3	108	191
9109	210	1.50	18	6.5	119	164
9110	210	1.50	19	6.3	98	110
9111	210	1.50	18	6.3	123	188
9112	210	1.50	21	6.4	111	134
9113	210	1.50	18	6.3	104	112
9156	240	0.00	20	6.5	115	211
9157	240	0.00	19	7.1	109	197

9158	240	0.00	18	6.5	91	174
9159	240	0.00	19	6.8	124	122
9160	240	0.00	21	6.8	102	183
9161	240	0.00	19	6.9	103	158
9161	240	0.00	18	6.7	103	147
9162	240	0.00	18	6.6	102	120
9163	240	0.00	19	6.9	126	170
9164	240	0.00	20	6.6	104	112
9165	240	0.00	18	7.3	130	119
9166	240	0.00	17	6.9	104	132
9167	240	0.00				
9144	240	0.25	19	6.5	94	122
9144	240	0.25	19	6.7	98	118
9145	240	0.25	22	7.0	93	145
9146	240	0.25	18	6.7	97	158
9147	240	0.25	18	7.0	116	237
9148	240	0.25	19	6.8	92	127
9148	240	0.25	18	6.6	97	117
9149	240	0.25	19	7.0	131	167
9150	240	0.25	18	6.7	107	222
9151	240	0.25	18	6.8	82	96
9152	240	0.25	19	7.1	96	81
9153	240	0.25	18	6.9	97	142
9154	240	0.25	19	7.1	101	168
9155	240	0.25				
9132	240	0.50	18	6.9	98	112
9133	240	0.50	18	6.9	109	185
9134	240	0.50	19	6.7	116	143
9135	240	0.50				
9136	240	0.50	19	7.0	122	249
9137	240	0.50	19	7.0	102	176
9138	240	0.50	18	7.1	102	111
9139	240	0.50	18	7.3	96	162
9140	240	0.50	18	6.9	115	152
9141	240	0.50	17	6.8	108	233
9142	240	0.50	17	6.9	114	117
9143	240	0.50	17	7.1	109	166
9120	240	1.00	19	6.6	86	139
9121	240	1.00	18	6.9	95	128
9122	240	1.00	18	6.7	98	175
9123	240	1.00	18	6.6	88	124
9124	240	1.00	18	6.9	114	148
9125	240	1.00	18	6.8	102	217
9126	240	1.00	19	6.9	104	125
9127	240	1.00	22	7.3	108	121
9128	240	1.00	18	6.8	115	188
9129	240	1.00	18	7.0	112	186
9130	240	1.00	18	6.8	102	121
9131	240	1.00				

9108	240	1.50	19	6.8	105	183
9108	240	1.50	17	6.7	109	166
9109	240	1.50	20	7.1	121	194
9110	240	1.50	20	6.6	95	117
9110	240	1.50	19	6.7	99	110
9111	240	1.50	19	6.8	105	202
9112	240	1.50	22	7.0	120	201
9113	240	1.50	19	7.1	116	130
9114	240	1.50	18	6.7	94	123
9115	240	1.50	16	6.8	93	121
9116	240	1.50	18	6.6	102	167
9117	240	1.50	16	6.6	106	125
9118	240	1.50	18	6.6	98	135
9119	240	1.50				
9156	270	0.00	18	6.6	131	235
9157	270	0.00	18	6.7	113	192
9158	270	0.00	17	6.4	98	118
9159	270	0.00	19	6.7	123	170
9160	270	0.00	18	6.5	144	120
9161	270	0.00	18	6.6	113	199
9144	270	0.25	18	6.2	103	153
9145	270	0.25	17	6.4	95	112
9146	270	0.25	17	6.6	106	166
9147	270	0.25	20	7.0	127	226
9148	270	0.25	17	6.5	93	133
9149	270	0.25	19	6.9	123	104
9132	270	0.50	20	6.8	101	105
9133	270	0.50	18	7.1	116	163
9134	270	0.50	18	6.4	123	163
9135	270	0.50				
9136	270	0.50	18	6.9	119	158
9137	270	0.50	19	6.9	106	105
9138	270	0.50	17	6.9	101	135
9139	270	0.50	18	7.0	103	148
9120	270	1.00	18	6.4	91	128
9121	270	1.00	18	6.7	114	146
9122	270	1.00	18	6.4	106	137
9123	270	1.00	18	6.9	108	105
9124	270	1.00	17	6.6	116	209
9125	270	1.00	18	6.4	106	172
9108	270	1.50	18	6.6	111	175
9109	270	1.50	19	6.6	120	139
9110	270	1.50	21	6.5	102	93
9111	270	1.50	15	6.2	91	51
9112	270	1.50	19	6.9	126	153
9113	270	1.50	17	7.2	121	126

Minimal underlying data set for S3.

Risk factors for cardiac disease (total cholesterol - Chol, triglycerides - TRIGS) and kidney injury (blood urea nitrogen - BUN, total protein - TP) in the serum of male WAG/RijCmcr rats after whole body exposure with silicon (28Si) ions.

Rat #	Blood collection (Days post 28Si exposure)	Total 28Si Dose (Gy	BUN mg/dL	TP (g/dl)	Chol (mg/dl)	TRIGS (mg/dl)
9216M (11)	30	0	17	5.8	67	62
9217M (12)	30	0	16	6	65	64
9218M (13)	30	0	18	5.9	66	71
9219M (14)	30	0	16	5.7	70	94
9220M (15)	30	0	18	6.1	81	130
9204M (6)	30	25	20	6.2	66	57
9205M (7)	30	25	19	6.1	67	112
9206M (8)	30	25	19	6.3	76	143
9207M (9)	30	25	21	6.1	83	157
9208M (10)	30	25	21	6.2	83	109
9226	30	25	19	5.9	79	71
9209	30	0.5	19	6.1	75	67
9210	30	0.5	17	6.1	96	148
9211	30	0.5	19	6.2	90	162
9212	30	0.5	19	6.2	87	156
9213	30	0.5	18	6	75	85
9214	30	0.5	18	6.3	93	119
9192	30	0.75	20	6.1	92	116
9193	30	0.75	17	6.3	91	134
9194	30	0.75	18	6	86	131
9195	30	0.75	19	6.2	86	105
9196	30	0.75	20	6	86	124
9197	30	0.75	17	6.2	87	116
9180M (1)	30	1.5	20	5.8	79	106
9181M (2)	30	1.5	20	6.4	76	103
9182M (3)	30	1.5	20	6.4	90	119
9183M (4)	30	1.5	19	6.2	72	85
9184M (5)	30	1.5	21	6.1	70	77
9185	30	1.5	19	6.3	76	95
9216M (11)	60	0	19	6.6	80	96
9217M (12)	60	0				
9218M (13)	60	0	15	5.6	69	78
9219M (14)	60	0	17	6.6	74	94

9220M (15)	60	0	18	6.8	77	101
9233	60	0	19	6.8	81	121
9234	60	0	18	6.9	92	153
9206M (8)	60	25	144	4.3	60	219
9204M (6)	60	25	19	6.6	76	65
9205M (7)	60	25	17	6.8	81	158
9206M (8)	60	25	18	7.1	97	119
9207M (9)	60	25	19	6.9	88	142
9208M (10)	60	25	18	7.2	84	86
9209	60	0.5	18	7	89	119
9210	60	0.5	18	6.9	92	138
9211	60	0.5	19	7.5	101	127
9212	60	0.5	16	6.8	88	71
9213	60	0.5	18	6.8	87	111
9214	60	0.5	18	6.8	89	90
9192	60	0.75	19	6.5	95	116
9193	60	0.75	18	7.1	89	116
9194	60	0.75	20	6.5	78	108
9195	60	0.75	18	7	85	97
9196	60	0.75	19	6.8	90	74
9197	60	0.75	20	7	87	117
9180M (1)	60	1.5	18	6.6	81	116
9181M (2)	60	1.5	23	6.5	65	106
9182M (3)	60	1.5	21	7.1	86	93
9183M (4)	60	1.5	18	6.6	78	121
9184M (5)	60	1.5	20	6.8	77	89
9185	60	1.5	20	6.9	81	108
9216M (11)	90	0	14	6.1	78	111
9217M (12)	90	0	17	6.1	86	123
9218M (13)	90	0	15	6.1	87	103
9219M (14)	90	0	15	5.8	78	86
9220M (15)	90	0	15	5.8	75	89
9233	90	0	16	6.3	95	130
9204M (6)	90	25	18	6.5	89	145
9205M (7)	90	25	17	6.4	82	177
9206M (8)	90	25	18	6.4	106	167
9207M (9)	90	25	19	7.4	112	173
9208M (10)	90	25	19	6.5	103	97
9226	90	25	18	6	86	110
9209	90	0.5	18	6.1	96	161

9210	90	0.5	18	6.5	108	205
9211	90	0.5	19	6.3	104	183
9212	90	0.5	16	6.5	96	139
9213	90	0.5	17	6.1	84	120
9214	90	0.5	19	6.7	105	171
9215	90	0.5	16	6	81	105
9192	90	0.75	18	6	95	242
9193	90	0.75	17	6.3	90	184
9194	90	0.75	16	6.1	88	201
9195	90	0.75	17	6.6	98	229
9196	90	0.75	17	6.2	97	169
9197	90	0.75	18	6.5	97	152
9198	90	0.75	17	5.9	90	75
9180M (1)	90	1.5	17	6.3	85	155
9181M (2)	90	1.5	19	6.4	83	94
9182M (3)	90	1.5	17	6.7	102	187
9183M (4)	90	1.5	16	6.1	84	121
9184M (5)	90	1.5	16	6.4	88	124
9185	90	1.5	17	6.7	91	182
9216M (11)	120	0	20	6.5	100	174
9217M (12)	120	0	18	6.4	95	141
9218M (13)	120	0	21	6.7	107	155
9219M (14)	120	0	28	5.9	86	111
9220M (15)	120	0	19	6.1	91	129
9233	120	0	19	6.5	97	115
9234	120	0	19	6.2	91	167
9204M (6)	120	25	22	6.7	98	153
9205M (7)	120	25	20	6.5	95	173
9206M (8)	120	25	20	6.8	113	249
9207M (9)	120	25	20	5.9	93	147
9208M (10)	120	25	19	6.6	112	123
9226	120	25	16	5.9	84	120
9227	120	25	18	6.5	95	159
9209	120	0.5	18	6	82	83
9210	120	0.5	18	6.1	100	159
9211	120	0.5	19	6.5	112	162
9212	120	0.5	17	6.4	95	160
9213	120	0.5	17	6.5	98	145
9214	120	0.5	19	6.5	105	203
9215	120	0.5	21	6.4	88	165
9192	120	0.75	20	6.1	111	168

9193	120	0.75	19	6.5	96	174
9194	120	0.75	18	6.3	95	200
9195	120	0.75	17	6.4	101	205
9196	120	0.75	20	6.6	113	182
9197	120	0.75	19	6.6	104	167
9198	120	0.75	18	6	95	144
9180M (1)	120	1.5	18	6.5	96	201
9181M (2)	120	1.5	18	7	101	111
9182M (3)	120	1.5	19	6.7	111	137
9183M (4)	120	1.5	17	6.4	99	179
9184M (5)	120	1.5	21	6	96	152
9185	120	1.5	19	6.3	96	234
9186	120	1.5	19	6.7	103	131
9216M (11)	150	0	22	6.6	95	168
9217M (12)	150	0	20	6.8	99	162
9218M (13)	150	0	20	6.6	104	121
9219M (14)	150	0	17	6.5	93	168
9220M (15)	150	0	20	6.9	139	68
9233	150	0	19	6.7	98	169
9234	150	0	18	6.5	100	162
9204M (6)	150	25	18	6.4	95	148
9205M (7)	150	25	17	6.4	92	134
9206M (8)	150	25	20	6.5	111	105
9207M (9)	150	25	18	6.7	109	169
9208M (10)	150	25	20	6.9	116	157
9226	150	25	17	6.6	100	197
9227	150	25	19	6.7	122	164
9209	150	0.5	21	6.7	110	189
9210	150	0.5	19	6.7	118	243
9211	150	0.5	20	6.2	111	96
9212	150	0.5	18	6.6	102	129
9213	150	0.5	18	6.5	88	124
9214	150	0.5	17	6.7	104	149
9215	150	0.5	18	6.5	102	139
9192	150	0.75	20	6.7	130	156
9193	150	0.75	19	6.8	106	119
9194	150	0.75	20	6.3	102	94
9195	150	0.75	20	6.6	115	91
9196	150	0.75	17	6.5	109	108
9197	150	0.75	20	6.8	99	128
9198	150	0.75	19	6.6	96	103

9180M (1)	150	1.5	17	6.6	94	128
9181M (2)	150	1.5	19	6.8	95	123
9182M (3)	150	1.5	21	6.7	104	94
9183M (4)	150	1.5	17	7	114	117
9184M (5)	150	1.5	19	6.4	97	120
9185	150	1.5	18	6.8	105	133
9186	150	1.5	18	6.4	93	131
9216M (11)	180	0	21	6.3	89	131
9217M (12)	180	0	20	6.5	101	179
9218M (13)	180	0	21	6.4	110	246
9219M (14)	180	0	20	6.2	97	185
9220M (15)	180	0	21	6.6	100	165
9233	180	0	20	6.6	106	222
9234	180	0	20	6.7	107	208
9204M (6)	180	25	19	6.5	101	158
9205M (7)	180	25	20	6.4	99	128
9206M (8)	180	25	21	6.3	117	219
9207M (9)	180	25	22	6.5	102	171
9208M (10)	180	25				
9226	180	25	22	6.6	94	171
9227	180	25	19	6.5	107	189
9228	180	25	25	6.2	92	194
9209	180	0.5	21	6.2	98	165
9210	180	0.5	21	6.4	116	166
9211	180	0.5	21	6.6	111	210
9212	180	0.5	20	6.2	107	196
9213	180	0.5	18	6.3	94	109
9214	180	0.5	19	6.7	114	192
9215	180	0.5	20	6.4	104	136
9192	180	0.75	21	6.3	120	200
9193	180	0.75	21	6.4	102	181
9194	180	0.75	21	6.1	96	147
9195	180	0.75	22	6.5	103	160
9196	180	0.75	20	5.9	121	134
9197	180	0.75				
9198	180	0.75	19	6.3	97	109
9180M (1)	180	1.5	19	6.8	101	144
9181M (2)	180	1.5	19	6.6	91	131
9182M (3)	180	1.5	22	6.7	109	147
9183M (4)	180	1.5	19	6.8	107	144
9184M (5)	180	1.5	19	6.3	99	148
9185	180	1.5	20	6.8	121	150

9216M (11)	210	0	21	6	87	111
9217M (12)	210	0	19	6.6	99	164
9218M (13)	210	0	19	6.2	93	130
9219M (14)	210	0	16	5.8	92	104
9220M (15)	210	0	16	6.4	91	92
9233	210	0	17	6.2	106	150
9204M (6)	210	25	18	6.2	92	103
9205M (7)	210	25	17	6.5	87	121
9206M (8)	210	25	18	6.5	107	190
9207M (9)	210	25	19	6.4	99	125
9208M (10)	210	25	20	6.7	118	111
9226	210	25	18	6.2	85	95
9209	210	0.5	19	6.5	97	114
9210	210	0.5	19	6.5	104	132
9211	210	0.5	19	6.4	102	94
9212	210	0.5	17	6	92	87
9213	210	0.5	19	6.4	86	121
9214	210	0.5	16	6.4	101	177
9192	210	0.75	19	6.4	118	172
9193	210	0.75	20	6.2	95	102
9194	210	0.75	19	6.1	88	112
9195	210	0.75	18	6.6	104	114
9196	210	0.75	19	6.1	119	111
9197	210	0.75	19	6.4	83	78
9180M (1)	210	1.5	19	6.9	96	106
9181M (2)	210	1.5	19	6.4	93	97
9182M (3)	210	1.5	19	6.3	96	98
9183M (4)	210	1.5	18	6.6	108	114
9184M (5)	210	1.5	17	6	86	92
9185	210	1.5	18	6.3	100	113
9216M (11)	240	0	18	6.2	91	164
9217M (12)	240	0	18	6.2	99	148
9218M (13)	240	0	20	6.4	101	146
9219M (14)	240	0	19	5.8	91	182
9220M (15)	240	0	18	6.5	93	137
9233	240	0	18	6.4	102	164
9204M (6)	240	25	17	6.2	93	124
9205M (7)	240	25	19	6.3	89	131
9206M (8)	240	25	17	6.4	108	102
9207M (9)	240	25	17	6.4	99	194

9208M (10)	240	25	19	6.5	118	<9
9226	240	25	20	6.3	90	178
9209	240	0.5	16	6	100	81
9210	240	0.5	16	6.3	106	127
9211	240	0.5	19	6.4	114	170
9212	240	0.5	17	6.5	112	157
9213	240	0.5	17	5.9	85	113
9214	240	0.5	16	6.4	94	122
9192	240	0.75	18	6.2	107	171
9193	240	0.75	18	6.3	97	119
9194	240	0.75	18	6.1	83	139
9195	240	0.75	18	6.2	98	207
9196	240	0.75	17	6.1	99	227
9197	240	0.75	18	6.2	83	102
9180M (1)	240	1.5	17	6.9	100	122
9181M (2)	240	1.5	17	7.3	103	100
9182M (3)	240	1.5	18	7	112	115
9183M (4)	240	1.5	17	7	124	150
9184M (5)	240	1.5	17	6.5	94	78
9216M (11)	270	0	19	6.6	100	143
9217M (12)	270	0	16	6.7	113	155
9218M (13)	270	0	20	6.7	113	96
9219M (14)	270	0	15	6.3	95	153
9220M (15)	270	0	17	6.7	101	140
9233	270	0	19	6.8	116	205
9234	270	0	18	6.7	106	164
9204M (6)	270	25	17	6.5	88	80
9205M (7)	270	25	17	6.5	101	92
9206M (8)	270	25	19	6.8	119	120
9207M (9)	270	25	19	6.5	99	148
9208M (10)	270	25	18	6.5	111	135
9226	270	25	19	6.2	102	119
9227	270	25	20	6.6	116	175
9209	270	0.5	17	6.4	107	116
9210	270	0.5	17	6.1	98	88
9211	270	0.5	17	6.9	130	90
9212	270	0.5	16	6.7	130	117
9213	270	0.5	17	6.5	107	153
9214	270	0.5	15	6.6	109	224
9215	270	0.5	19	6.5	98	127

9192	270	0.75	20	6.5	119	165
9193	270	0.75	18	6.8	105	117
9194	270	0.75	16	6.4	111	109
9195	270	0.75	17	6.9	117	141
9196	270	0.75	16	6.7	110	183
9197	270	0.75	18	6.2	95	141
9198	270	0.75	17	6.5	111	131
9180M (1)	270	1.5	16	7	101	116
9181M (2)	270	1.5	17	7.3	112	97
9182M (3)	270	1.5	16	6.6	99	94
9183M (4)	270	1.5	18	6.9	117	122
9184M (5)	270	1.5	17	6.5	100	66
9185	270	1.5	18	6.7	122	92
9186	270	1.5	19	6.4	98	116

Minimal underlying data set for S4.

Risk factors for cardiac disease (total cholesterol - Chol, triglycerides - TRIGS) and kidney injury (blood urea nitrogen - BUN, total protein - TP) in the serum of male WAG/RijCmcr rats after whole body exposure with iron (56Fe) ions.

Rat #	Blood collection (Days post exposure)	Total 56Fe Dose (Gy)	BUN mg/dL	TP (g/dl)	Chol (mg/dl)	TRIGS (mg/dl)
9000	30	0	16	6.7	95	158
9001	30	0	17	6.6	92	108
9002	30	0	15	6.5	91	115
9003	30	0	15	6.5	75	104
9004	30	0	16	6.2	78	130
9046	30	0	16	6.5	88	104
9047	30	0	17	6.6	82	108
9048	30	0	16	6.6	86	127
9005	30	0.1	16	6.3	77	93
9006	30	0.1	16	6.2	69	98
9008	30	0.1	15	6.1	71	132
9053	30	0.1	16	6.4	83	127
9054	30	0.1	20	6.5	81	84
9055	30	0.1	18	6.5	96	79
9034	30	0.25	15	6.3	83	114
9035	30	0.25	17	6.4	84	109
9036	30	0.25	16	6.7	86	75
9037	30	0.25	16	6.4	77	106
9038	30	0.25	15	6.5	86	135
9039	30	0.25	16	6.3	80	117
9041	30	0.25	17	6.1	79	108
9022	30	0.5	15	6.4	83	149
9023	30	0.5	17	6.8	100	141
9024	30	0.5	16	6.5	84	172
9025	30	0.5	15	6.7	86	101
9026	30	0.5	15	6.7	81	114
9027	30	0.5	17	6.5	101	129
9028	30	0.5	18	6.4	87	140
9010	30	1	17	6.5	83	98
9011	30	1	15	6.8	103	116
9012	30	1	16	6.7	98	127
9013	30	1	17	6.3	85	148
9014	30	1	14	6.6	99	246
9015	30	1	17	6.3	93	131

9016	30	1	15	6.3	94	175
9000	60	0	21	6.3	105	174
9001	60	0	22	7.0	110	187
9003	60	0	20	6.9	103	119
9004	60	0	23	6.6	97	174
9046	60	0	20	6.7	101	207
9047	60	0	20	6.9	102	146
9048	60	0	20	6.4	99	138
9049	60	0	19	6.9	102	199
9050	60	0	20	6.7	118	192
9051	60	0	20	6.9	101	174
9052	60	0	18	6.9	110	174
9005	60	0.1	21	6.5	101	219
9006	60	0.1	22	6.3		
9007	60	0.1	19	6.5	95	154
9008	60	0.1	17	6.5	93	102
9009	60	0.1	18	6.2	88	
9053	60	0.1	18	6.5	103	167
9054	60	0.1	19	6.6	107	187
9056	60	0.1	21	6.7	120	204
9057	60	0.1	20	7.2	109	117
9034	60	0.25	17	6.6	102	188
9035	60	0.25	19	6.3	103	166
9036	60	0.25	18	7.0	111	125
9038	60	0.25	20	6.5	107	180
9039	60	0.25	20	6.5	104	173
9041	60	0.25	19	7.0	117	185
9043	60	0.25	17	6.7	105	173
9022		0.5				
9023	60	0.5	18	6.9	116	193
9024	60	0.5	18	6.5	101	226
9025	60	0.5	20	6.6	106	199
9026	60	0.5	20	7.0	112	161
9027	60	0.5	21	7.0	111	138
9028	60	0.5	20	6.3	100	192
9010	60	1	20	6.3	93	174
9011	60	1	21	6.9	103	144
9012	60	1	18	7.0	107	229
9013	60	1	22	6.8	115	192
9014	60	1	19	6.2	110	277
9015	60	1	20	6.5	95	154

9000	90	0	17	6.8	110	135
9001	90	0	18	6.7	110	97
9002	90	0	17	6.7	102	161
9003	90	0	17	7.0	95	111
9004	90	0	19	6.6	94	145
9047	90	0	20	6.7	90	135
9050	90	0	18	6.6	112	161
9005	90	0.1	18	6.7	97	135
9006	90	0.1	20	6.6	82	90
9007	90	0.1	17	6.8	94	112
9008	90	0.1	18	6.8	93	160
9009	90	0.1	20	6.8	95	97
9055	90	0.1	19	7.0	103	150
9034	90	0.25	17	6.6	87	158
9035	90	0.25	17	6.6	102	195
9036	90	0.25	19	6.6	102	137
9037	90	0.25	18	6.6	89	123
9038	90	0.25	19	6.6	101	216
9039	90	0.25	18	6.6	84	143
9040	90	0.25	18	6.9		
9022	90	0.5	16	6.6	87	145
9023	90	0.5	18	7.2	124	132
9024	90	0.5	17	6.5	91	134
9025	90	0.5	19	7.2	115	102
9026	90	0.5	18	6.9	109	172
9027	90	0.5	18	6.8	115	143
9028	90	0.5	18	6.6	101	179
9010	90	1	18	6.6	93	109
9011	90	1	18	6.9	110	124
9012	90	1	17	7.0	106	168
9013	90	1	19	6.8	102	166
9014	90	1	18	6.6	97	162
9015	90	1	19	6.8	107	164
9000	119	0	18	6.6	110	121
9001	119	0	17	6.7	108	121
9002	119	0	17	6.2	97	128
9003	119	0	15	5.9	91	128
9004	119	0	19	6.5	102	131
9046	119	0	17	6.4	104	181
9047	119	0	19	7.0	114	120
9005	119	0.1	19	6.7	103	130

9006	119	0.1	18	6.5	91	114
9007	119	0.1	16	6.5	91	156
9008	119	0.1	18	6.3	100	163
9009	119	0.1	17	6.6	103	142
9053	119	0.1	17	6.6	102	145
9054	119	0.1	21	6.8	111	132
9034	119	0.25	16	6.1	98	167
9035	119	0.25	17	6.6	112	159
9036	119	0.25	19	6.7	113	119
9037	119	0.25	19	6.6	95	153
9038	119	0.25	18	6.4	107	141
9039	119	0.25	18	6.6	96	120
9040	119	0.25	19	6.4	140	125
9022	119	0.5	17	6.6	94	170
9023	119	0.5	17	6.9	121	136
9024	119	0.5	18	6.2	90	154
9025	119	0.5	18	6.7	115	144
9026	119	0.5	16	6.5	116	117
9027	119	0.5	18	6.3	118	105
9028	119	0.5	17	6.4	101	163
9029	119	0.5	19	6.8	110	114
9030	119	0.5	16	6.6	113	161
9010	119	1	18	6.3	101	116
9011	119	1	18	6.8	121	147
9012	119	1	16	6.8	116	172
9013	119	1	18	6.2	101	155
9014	119	1	18	6.5	118	155
9015	119	1	20	6.7	121	191
9016	119	1	18	6.4	109	171
9017	119	1	17	6.6	109	180
9019	119	1	17	6.6	123	183
9020	119	1	17	6.4	112	178
9021	119	1	17	6.9	126	183
9000	146	0	15	6.3	98	122
9001	147	0	17	6.8	112	105
9002	148	0	16	6.3	96	141
9003	149	0	14	6.5	87	123
9004	150	0	15	6.4	96	108
9046	151	0	17	6.4	92	104
9047	152	0	17	6.8	99	96
9005	146	0.1	16	6.6	94	160
9006	147	0.1	17	6.7	86	113

9007	148	0.1	15	6.6	91	112
9008	149	0.1	16	6.6	93	162
9009	150	0.1	18	6.9	95	159
9053	151	0.1	15	6.5	91	139
9054	152	0.1	18	6.6	101	147
9034	146	0.25	17	6.1	72	91
9035	147	0.25	16	6.5	94	166
9036	148	0.25	17	6.7	101	108
9037	149	0.25	16	6.6	92	163
9038	150	0.25	17	6.8	105	162
9039	151	0.25	17	6.8	96	148
9040	152	0.25	15	6.6	102	133
9022	146	0.5	15	6.5	84	116
9023	147	0.5	17	6.7	112	146
9024	148	0.5	14	6.2	79	136
9025	149	0.5	17	6.7	96	136
9026	150	0.5	15	6.5	94	134
9027	151	0.5	18	6.6	93	98
9028	152	0.5	15	6.5	98	138
9010	146	1	15	6.5	89	110
9011	147	1	15	6.6	110	167
9012	148	1	15	6.6	102	123
9013	149	1	17	6.7	107	220
9014	150	1	16	6.5	111	233
9015	151	1	18	6.6	109	160
9016	152	1	15	6.3	96	117
9000	178	0	16	6.9	106	146
9001	178	0	18	6.8	118	140
9002	178	0	17	6.6	102	208
9003	178	0	16	6.4	93	133
9004	178	0	19	6.7	97	147
9046	178	0	17	6.5	104	166
9047	178	0	18	6.9		
9005	178	0.1	19	6.5	87	157
9006	178	0.1	22	6.7	96	86
9007	178	0.1	19	6.6	92	210
9008	178	0.1	18	6.2	90	197
9009	178	0.1	19	6.6	95	163
9053	178	0.1	17	6.7	123	103
9054	178	0.1	18	6.7	111	162
9034	178	0.25	17	6.5	95	176

9035	178	0.25	19	6.7	107	232
9036	178	0.25	19	6.7	98	160
9037	178	0.25	18	6.6	91	160
9038	178	0.25	17	6.5	100	132
9039	178	0.25	15	6.7	91	88
9040	178	0.25	17	6.5	101	110
9022	178	0.5	17	6.4	94	198
9023	178	0.5	18	6.9	121	234
9024	178	0.5	18	6.4	90	222
9025	178	0.5	19	7.0	115	197
9026	178	0.5	15	6.9	129	18
9027	178	0.5	17	6.6	121	156
9028	178	0.5	18	6.7	99	196
9010	178	1	17	6.5	92	116
9011	178	1	16	6.8	122	145
9012	-42174	1	21	6.2	71	66
9013	-42174	1	20	6.9	109	231
9014	178	1	18	7.0	113	201
9015	178	1	18	6.8	125	163
9016	178	1	17	6.3	94	189
9000	210	0	17	6.0	109	109
9001	208	0	20	7.1	124	176
9002	210	0	17	6.2	109	128
9003	208	0	19	6.7	102	153
9004	208	0	18	6.6	102	105
9046	210	0	17	6.7	108	167
9047	210	0	16	6.9	114	121
9005	208	0.1	17	6.2	94	141
9006	210	0.1	18	6.5	102	140
9007	208	0.1	18	6.6	94	144
9008	208	0.1	18	6.1	85	136
9009	208	0.1	19	6.3	96	156
9053	210	0.1	18	6.6	100	115
9054	210	0.1	16	6.7	118	146
9034	208	0.25	18	6.6	94	175
9035	208	0.25	21	6.7	102	173
9036	208	0.25	19	6.9	113	144
9037	208	0.25	18	6.6	90	128
9038	208	0.25	19	6.7	102	143
9039	208	0.25	20	6.7	89	105
9040	208	0.25	19	6.6	99	144

9022	-42174	0.5	18	6.8	97	154
9023	208	0.5	20	7.0	115	127
9024	-42174	0.5	18	6.7	94	173
9025	-42174	0.5	19	7.1	120	151
9026	-42174	0.5	20	6.7	107	162
9027	-42174	0.5	19	7.1	124	187
9028	-42174	0.5	18	6.5		
9010	208	1	17	6.0	89	133
9011	208	1	18	6.8	122	150
9012	208	1	18	6.0	83	43
9013	210	1	21	6.4	110	146
9014	210	1	17	6.8	124	156
9015	210	1	27	6.5	124	76
9016	208	1	18	6.4	99	139
9000	241	0	18	6.4	95	132
9001	245	0	20	6.6	119	146
9002	241	0	18	6.4	115	140
9003	241	0	19	6.8	112	140
9004	241	0	20	6.6	113	109
9046	241	0	17	6.2	99	125
9047	241	0	18	7.0	119	177
9005	241	0.1	20	6.2	100	94
9006	241	0.1	18	6.6	99	177
9007	241	0.1	18	6.5	93	172
9008	241	0.1	17	6.4	91	140
9009	241	0.1	18	6.4	105	107
9053	241	0.1	18	6.0	88	102
9054	241	0.1	18	6.4	117	112
9034	241	0.25	17	6.3	97	163
9035	241	0.25	17	6.5	105	200
9036	241	0.25	17	6.3	113	169
9037	241	0.25	17	6.4	103	159
9038	241	0.25	17	6.3	114	162
9039	241	0.25	18	6.1	88	134
9040	241	0.25	18	6.1	98	151
9022	242	0.5	15	5.5	80	75
9023	241	0.5	20	6.2	123	179
9024	241	0.5	16	6.3	91	151
9025	241	0.5	21	6.6	110	109
9026	241	0.5	16	6.3	102	101
9027	241	0.5	19	6.6	117	124
9028	241	0.5	16	6.0	98	149

9010	241	1	18	6.1	84	126
9011	241	1	17	6.8	120	165
9012	241	1	28	5.0	51	42
9013	241	1	19	6.5	110	160
9014	241	1	17	6.8	124	156
9015	241	1	19	6.8	113	135
9016	241	1	16	6.2	102	135
9017	241	1	16	6.1	112	125
9000	270	0	18	6.3	98	138
9001	270	0	20	6.9	129	187
9002	270	0	19	6.4	119	193
9003	270	0	17	6.6	111	166
9004	270	0	19	6.5	107	162
9046	270	0	18	6.5	91	146
9047	270	0	17	6.9	100	179
9005	270	0.1	16	6.3	104	146
9006	270	0.1	19	6.5	104	144
9007	270	0.1	17	6.6	88	126
9008	270	0.1	18	6.3	104	169
9009	270	0.1	19	6.7	109	98
9053	270	0.1	19	6.1	89	95
9034	270	0.25	17	6.2	97	155
9035	270	0.25	17	6.7	114	166
9036	270	0.25	19	6.6	112	199
9037	270	0.25	19	6.5	95	163
9038	270	0.25	18	6.3	123	129
9039	270	0.25	18	6.6	97	110
9040	270	0.25	18	6.2	97	107
9022	-42174	0.5				
9023	270	0.5	17	6.5	106	109
9024	270	0.5	15	6.5	93	125
9025	272	0.5	18	6.7	107	149
9026	270	0.5	17	6.3	106	129
9027	270	0.5	19	6.6	114	163
9028	272	0.5	17	6.7	105	146
9029	270	0.5	19	6.5	105	125
9011	270	1	15	7.1	139	107
9013	270	1	16	6.5	121	157
9014	270	1	19	6.5	102	250
9015	270	1	19	6.7	114	161
9016	-42174	1	16	6.4	117	144

9017	270	1	18	6.7	110	132
9019	270	1	20	6.8	121	171

Minimal underlyinf data set for S6

Perivascular collagen content in hearts of male WAG/RijCmcr rat measured 270 days after whole body exposure to single ion beams of e.g. protons, silicon (28Si) or iron (56Fe).

Group	Rat/Slide ID#	Perivascular collagen content (% of lumenal earea)
**Protons (1000 MeV)**		
1.5Gy protons	9108 1.5Gy Protons	61
1.5Gy protons	9109 1.5Gy Protons	79
1.5Gy protons	9110 1.5Gy Protons	203
Sham	9156 Ctr 270d	106
Sham	9157 Ctr 270d	71
Sham	9158 Ctr 270d	63
**28Si (500 MeV/n)**		
1.5 Gy 28Si	918. 1.5Gy Silicon	378
1.5 Gy 28Si	9182 1.5Gy Silicon	436
1.5 Gy 28Si	9185 1.5Gy Silicon	178
Sham	9216 Ctr Silicon	246
Sham	9219 Ctr Silicon	197
Sham	9220 Ctr Silicon	279
**56Fe (600 MeV/n)**		
Sham	9000 Ctr Iron	84
Sham	9004 Ctr Iron	99
Sham	9046v Ctr Iron	67
1.0 Gy 56Fe	9013 1.0Gy Iron	276
1.0 Gy 56Fe	9014 1.0Gy Iron	158
1.0 Gy 56Fe	9015 1.0Gy Iron	95

Minimal underlying data set for S7.

Systemic blood pressure in male WAG/RijCmcr rat mesured 270 days after whole body irradiation with single ion beams of protons, silicon (28Si) or iron (56Fe).

Proton studies

Group	Speciment	Systolic	Mean	Diastolic
Sham	9156	106	86	71
Sham	9157	126	86	65
Sham	9158	111	84	71
Sham	9159	106	74	53
Sham	9160	115	81	63
Sham	9161	111	73	54
Sham	9162	109	74	56
Sham	9163	120	93	80
0.25Gy	9144	121	87	70
0.25Gy	9145	116	75	54
0.25Gy	9146	120	77	55
0.25Gy	9147	104	74	59
0.25Gy	9148	118	88	73
0.25Gy	9149	117	92	79
0.25Gy	9150	111	82	68
0.25Gy	9151	117	88	74
0.5Gy	9132	125	92	75
0.5Gy	9133	106	63	42
0.5Gy	9136	117	94	83
0.5Gy	9137	102	70	55
0.5Gy	9138	118	83	66
0.5Gy	9139	120	83	65
0.5Gy	9142	115	96	87
0.5Gy	9143	114	75	56
1Gy	9120	112	76	57

28Si studies

Group	Speciment	Systolic	Mean	Diastolic
0.25Gy	9204	112	79	63
0.25Gy	9205	109	81	67
0.25Gy	9226	131	105	91
0.25Gy	9227	126	81	63
0.25Gy	9228	125	94	79
0.25Gy	9229	125	93	77
0.25Gy	9230	141	100	79
0.25Gy	9231	128	93	75
0.5Gy	9209	126	89	69
0.5Gy	9210	121	84	66
0.5Gy	9211	116	87	73
0.5Gy	9212	117	89	74
0.5Gy	9213	118	81	63
0.5Gy	9214	138	96	76
0.5Gy	9224	129	95	79
0.5Gy	9225	109	76	56
0.75Gy	9194	132	101	85
0.75Gy	9195	132	90	68
0.75Gy	9196	118	89	74
0.75Gy	9197	114	84	68
0.75Gy	9198	119	81	63
0.75Gy	9199	117	85	69
0.75Gy	9200	124	94	79
0.75Gy	9201	114	90	78
1.5Gy	9180	119	83	66

56Fe studies

Group	Speciment	Systolic	Mean	Diastolic
0.1Gy	9006	126	86	67
0.1Gy	9007	119	75	53
0.1Gy	9008	117	88	73
0.1Gy	9009	111	68	57
0.1Gy	9054	110	82	68
0.1Gy	9055	131	97	80
0.1Gy	9059	114	76	61
0.1Gy	9058	110	75	65
0.1Gy	9057	118	87	72
0.1Gy	9053	119	84	66
0.1Gy	9005	119	84	67
0.25Gy	9034	132	97	80
0.25Gy	9035	132	96	77
0.25Gy	9036	109	86	74
0.25Gy	9037	109	75	59
0.25Gy	9038	119	89	73
0.25Gy	9039	121	87	70
0.25Gy	9040	124	93	76
0.25Gy	9041	143	116	103
0.25Gy	9045	119	86	70
0.25Gy	9044	117	93	81
0.25Gy	9043	123	95	81
0.25Gy	9042	125	90	73
0.5Gy	9022	128	88	68
0.5Gy	9023	114	85	71
0.5Gy	9024	115	82	65

1Gy	9121	116	80	62
1Gy	9122	117	84	67
1Gy	9123	114	81	64
1Gy	9124	108	82	69
1Gy	9125	114	86	72
1Gy	9126	110	79	63
1Gy	9127	113	75	56
1.5Gy	9112	116	82	65
1.5Gy	9113	110	83	69
1.5Gy	9114	125	82	61
1.5Gy	9115	114	66	41
1.5Gy	9116	116	88	74
1.5Gy	9117	115	90	77
1.5Gy	9118	112	75	57
1.5Gy	9119	113	77	59

1.5Gy	9181	112	80	64
1.5Gy	9182	107	82	70
1.5Gy	9183	108	73	56
1.5Gy	9184	102	71	54
1.5Gy	9185	122	88	70
1.5Gy	9186	123	88	69
1.5Gy	9187	129	95	77
Sham	9216	113	82	66
Sham	9217	119	88	72
Sham	9219	102	73	56
Sham	9220	119	82	63
Sham	9234	124	87	69
Sham	9235	118	84	68
Sham	9236	123	90	71
Sham	9237	116	82	66

0.5Gy	9025	121	77	55
0.5Gy	9026	122	90	74
0.5Gy	9027	117	81	63
0.5Gy	9028	132	94	75
0.5Gy	9029	130	93	75
0.5Gy	9032	109	71	52
0.5Gy	9033	117	85	69
0.5Gy	9031	122	85	67
0.5Gy	9030	108	81	68
1Gy	9010	115	87	73
1Gy	9011	115	75	55
1Gy	9013	108	77	62
1Gy	9012	112	77	60
1Gy	9014	123	90	73
1Gy	9015	117	83	66
1Gy	9016	141	106	89
1Gy	9017	137	103	86
1Gy	9021	105	67	46
1Gy	9020	125	98	84
1Gy	9019	109	88	77
Sham	9000	127	94	78
Sham	9001	120	78	58
Sham	9002	124	92	77
Sham	9003	118	85	68
Sham	9046	108	77	61
Sham	9047	105	74	58
Sham	9048	111	80	64
Sham	9049	127	89	70
Sham	9051	122	83	64
Sham	9050	111	84	71
Sham	9052	122	89	73
Sham	9004	125	99	87

Minimal underlying data set for S8 and S10.

Renal fibrosis measured at 270 days after total body irradiation with single ion (protons or 28Si or 56Fe) or sequentially delivered 3-ion (protons+28Si+56Fe) beams.

Total dose/ion(s)	Rat ID#	Blue area (BA)	Total tissue area (TTA)	Percent BA vs. TTA
Sham Fe	9000	3179705	175836120	1.81
Sham Fe	9001	7528432	197929686	3.80
Sham Fe	9004	3063464	161149906	1.90
1.0 Gy Fe	9013	7382589	150511591	4.90
1.0 Gy Fe	9014	2483663	110986889	2.24
1.0 Gy Fe	9015	10580221	151812435	6.97
Sham Protons	9156	2171580	162842828	1.33
Sham Protons	9157	2595189	165858614	1.56
Sham Protons	9158	1289248	143004967	0.90
1.5 Gy Protons	9108	1744712	164032839	1.06
1.5 Gy Protons	9109	1352682	158735101	0.85
1.5 Gy Protons	9110	983175	132156399	0.74
Sham Si	9233	724957	139063228	0.52
Sham Si	9234	974276	117032399	0.83
Sham Si	9235	1095158	127616372	0.86
1.5 Gy Si	9181	1906659	144330141	1.32
1.5 Gy Si	9182	1963363	164546852	1.19
1.5 Gy Si	9185	1245412	155094868	0.80
Sham Mixed 3 beams	9270	524909	153221246	0.34
Sham Mixed 3 beams	9271	1086852	130741935	0.83
Sham Mixed 3 beams	9272	978731	118244789	0.83
Sham Mixed 3 beams	9273	1652736	113833990	1.45
Sham Mixed 3 beams	9274	1576164	116897809	1.35
Sham Mixed 3 beams	9275	1802399	136201221	1.32
1.5 Gy Mixed 3 beams	9333	1276147	116230971	1.10
1.5 Gy Mixed 3 beams	9334	792793	107212734	0.74
1.5 Gy Mixed 3 beams	9335	1402155	144924908	0.97
1.5 Gy Mixed 3 beams	9336	1377032	112511311	1.22
1.5 Gy Mixed 3 beams	9337	1089086	119405540	0.91
1.5 Gy Mixed 3 beams	9338	1197494	104134048	1.15

Minimal underlying data set for S9.

Results of echocardigraphic measurements of cardiac function 270 days after whole body exposure to sequentially delivered beams of protons, 28Si, and 56Fe.

Dose	Rat ID	Measurement	Radial Strain						Circumferential Strain					
			AS	ANT	LAT	POST	INF	SEP	AS	ANT	LAT	POST	INF	SEP
1.5Gy	9264	1	35.21	32.53	21.72	11.12	8.74	19.41	-24.13	-22.61	-7.82	-2.81	-13.66	-14.52
1.5Gy	9264	2	29.03	38.12	39.51	29.93	22.35	21.3	-24.1	-26.28	-9.89	-3.64	-15.31	-11.66
1.5Gy	9264	3	43.47	49.61	49.46	31.13	20.87	23.95	-24.18	-27.67	-10.77	6.49	10.01	15.77
1.5Gy	9265	1	25.17	38.96	47.31	41.22	34.7	27.33	-26.95	-24.39	-7.11	1.57	-13.81	-19.09
1.5Gy	9265	2	52.5	61.03	63.1	55.8	52.62	50.98	-27.17	-23.83	-4.36	4.06	-9.07	-17.42
1.5Gy	9265	3	47.73	47.03	39.21	30.68	30.29	33.48	-25.32	-19.85	-1.38	11.64	-11.67	-23.69
1.5Gy	9266	1	60.71	56.24	60.72	52.42	49.46	51.36	-26.02	-28.3	-20.37	-8.64	-9.44	-14.45
1.5Gy	9266	2	55.84	59.84	54.67	42.57	36.03	36.15	-24.36	-26.97	-19.8	-3.83	-8.39	-14.26
1.5Gy	9266	3	38.35	52.79	71.09	75.92	66.51	48.55	-23.15	-30.07	-24.53	-9.54	-7.25	-5.81
1.5Gy	9267	1	44.45	51.73	57.31	45.25	38.12	39.14	-27.28	-25.03	-7.7	6.69	-8.14	-17.45
1.5Gy	9267	2	59.01	64.15	67.39	61.81	65.5	57.2	-33.06	-27.92	-0.31	16.8	-10.62	-26.76
1.5Gy	9267	3	49.29	46.83	54.49	50.84	52.23	52.4	-24.63	-25.69	-9.29	-2.28	-13.13	-12.98
1.5Gy	9268	1	26.54	41.09	52.9	44.66	31.04	20.23	-15.49	-21.55	-10.24	-2.52	-19.63	-18.27
1.5Gy	9268	2	22.65	38.63	53.57	51.95	36.88	20.49	-10.91	-20.77	-13.19	-2.93	-19.33	-17.94
1.5Gy	9268	3	44.33	45.15	43.61	40.89	36.94	35.54	-21.69	-21.99	-12.53	-6.98	-15.38	-19.53
1.5Gy	9269	1	43.71	46.51	57.98	42.91	38.2	37.84	-28.46	-29.59	-11.83	2.2	-13.4	-20.57
1.5Gy	9269	2	43.83	47.45	55.24	40.02	35.07	36.02	-22.46	-23.08	-9.97	2.52	-8.22	-15.9
1.5Gy	9269	3	39.46	45.43	51.73	45.69	39.43	37.63	-20.08	-22.31	-11.89	8.31	-4.94	-17.35
Sham	9329	1	59.99	76.61	86.2	68.48	55.27	62.48	-27.6	-29.9	-20.36	-3.74	0.71	-7.08
Sham	9329	2	66.45	65.94	71.54	56.9	52.19	61.85	-27.58	-30.58	-18.32	-3.2	2.6	6.55
Sham	9329	3	89.73	87.19	81.75	71.22	68.32	76.75	-30.02	-30.16	-13.65	1.83	-1.17	-9.48
Sham	9330	1	72.74	95.78	101	77.1	59.29	51.61	-23.42	-24.79	-20.7	0.34	-0.68	-21.41
Sham	9330	2	92.81	113	108	80.21	68.32	64.42	-25.66	-24.47	-16.93	2.08	-4.4	-25.81
Sham	9330	3	99.1	114	112	84.54	66.05	59.33	-21.38	-24	-20.11	2.4	-3.06	-24.75
Sham	9331	1	38.34	35.21	30.38	20.36	21.04	26.56	-20.18	-27.52	-5.06	7.05	-11.34	-14.97
Sham	9331	2	38.69	52.76	62.14	42.87	33.84	31.47	-25.33	-29.65	-8.59	2.12	-15.19	-14.66

Sham	9331	3	44.33	54.27	50.96	34.05	29.41	32.76	-19.22	-24.16	-12.58	-5.07	-11.77	-11.65
Sham	9332	1	33.57	32.88	35.25	24.62	23.47	28.89	-20.17	-28.78	-13.52	2.57	-17.32	-16.18
Sham	9332	2	28.52	29.55	25.29	6.48	9.13	17.98	-19.12	-28.4	-12.05	1.17	-13.6	-13.18
Sham	9332	3	44.38	38.83	28.2	16.52	20.71	31.9	-18.75	-27.28	-11.09	-0.49	-15.68	
Sham	9333	1	48.43	60.53	55.92	40.4	34.6	37.3	-25.62	-27.14	-9.35	-3.68	-10.06	-13.49
Sham	9333	2	37.45	49.72	53.52	47	39.39	34.19	-19.03	-23.62	-10.93	-4.69	-14.8	-16.26
Sham	9333	3	64.5	66.46	64.64	56.54	56	60.6	-23.62	-25.7	-11.03	-7	-15.26	-15.97
Sham	9334	1	35.52	35.46	33.71	22.73	23.28	29.6	-21.09	-23	-6.34	4.19	-12.69	-13.42
Sham	9334	2	24.7	32.25	32.96	20.55	16.72	17.7	-17.48	-21.65	-4.29	-0.7	-14.83	-11.6
Sham	9334	3	44.13	49.21	48.87	44.13	37.78	33.1	-18.31	-22.51	-10.35	2.2	-10.62	-15.25

**Key**		**Updated Key**
AS	Anterior Septal	Anteroseptum
ANT	Anterior	Anterior
LAT	Lateral	Anterolateral
POST	Posterior	Inferolateral
INF	Inferior	Inferior
SEP	Septal	Inferoseptal
