# Supplementary material for: Exposure to multiple ion beams, broadly representative of galactic cosmic rays, causes perivascular cardiac fibrosis in mature male rats
Source: PLoS One. 2023 Apr 26;18(4):e0283877. doi: 10.1371/journal.pone.0283877 (PMC10132632; doi:10.1371/journal.pone.0283877)
Supplement: S2 File — (PDF) [file pone.0283877.s002.pdf]

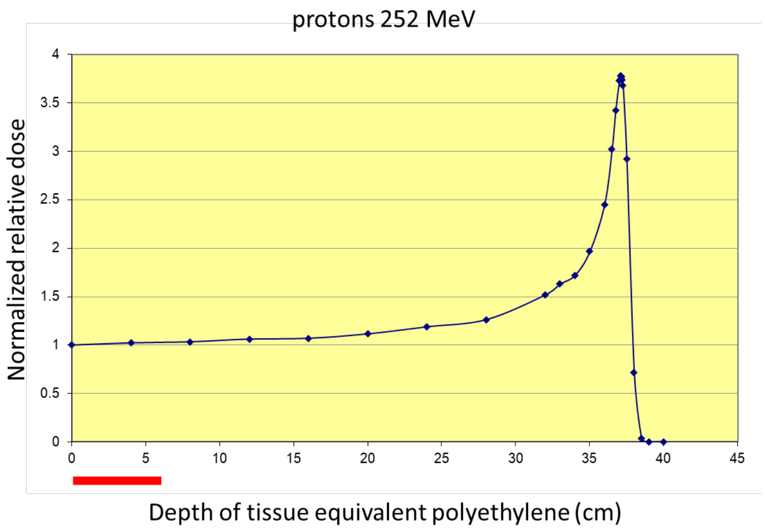
protons 252 MeV
Normalized relative dose
4
3.5
3
2.5
2
1.5
1
0.5
0
0
5
10
15
20
25
30
35
40
45
Depth of tissue equivalent polyethylene (cm)

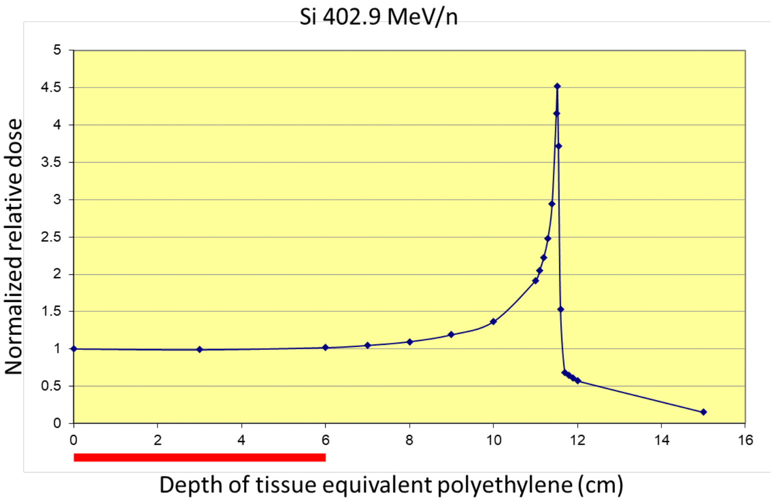
Si 402.9 MeV/n
Normalized relative dose
5
4.5
4
3.5
3
2.5
2
1.5
1
0.5
0
0
2
4
6
8
10
12
14
16
Depth of tissue equivalent polyethylene (cm)

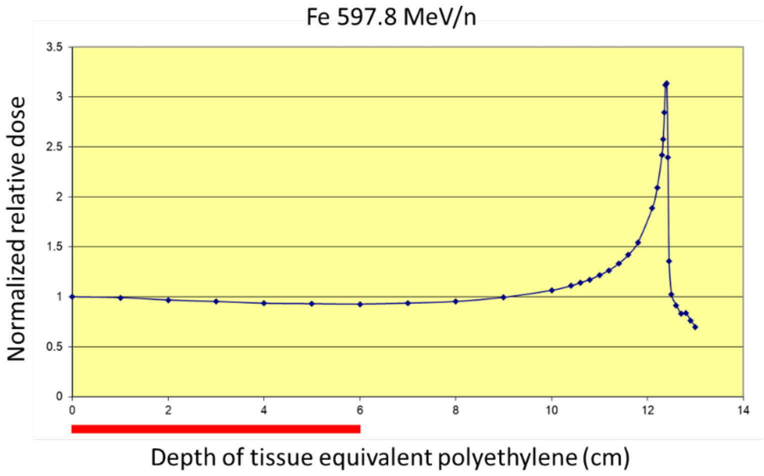
Fe 597.8 MeV/n
Normalized relative dose
3.5
3
2.5
2
1.5
1
0.5
0
0
2
4
6
8
10
12
14
Depth of tissue equivalent polyethylene (cm)

S1 Fig. Normalized depth dose curves for protons, ^{28}Si and ^{56}Fe of the energies specified and produced at the NSRL (1) in tissue equivalent, high density polyethylene (ρ = 0.97 g/cm^3). The horizontal red line shows the location of the 6 cm width of the Plexiglass restraint jig used to immobilize the rats for the period of irradiation in the initial 6 cm plateau region of the three Bragg peak depth dose distributions. Bragg curves shown are representative of the energies for individual proton (1000 MeV) and heavy ion (^{28}Si; 500 MeV/n and ^{56}Fe; 600 MeV/n) beams closest to those used in the present study.

1. [Available from: https://www.bnl.gov/nsrl/userguide/bragg-curves-and-peaks.php.

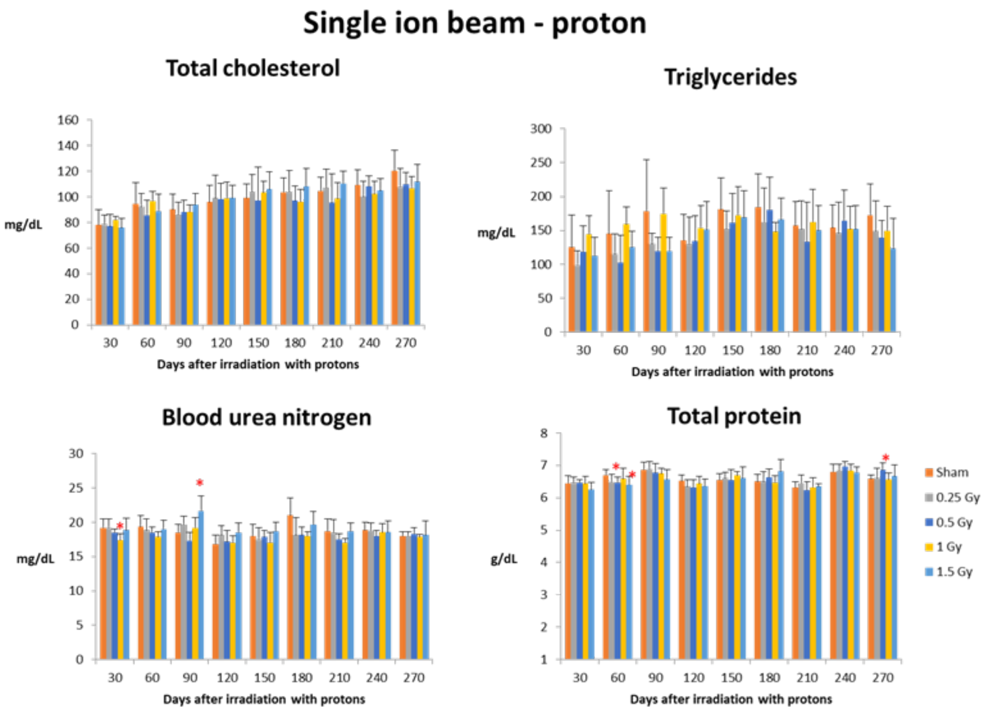

S2 Fig. Risk factors for cardiac disease and kidney injury after irradiation of rats with protons (1000 MeV). Total cholesterol, triglycerides, blood urea nitrogen and total protein levels in blood after irradiation with doses of 0.25, 0.50, 1.0 or 1.5 Gy of protons or sham-irradiation. Rats were six months of age at the time of irradiation or sham-irradiation. Data represent the mean ± SD, *n* = 6 - 12/group. * = $p < 0.05$ vs. age-matched sham-irradiated controls.

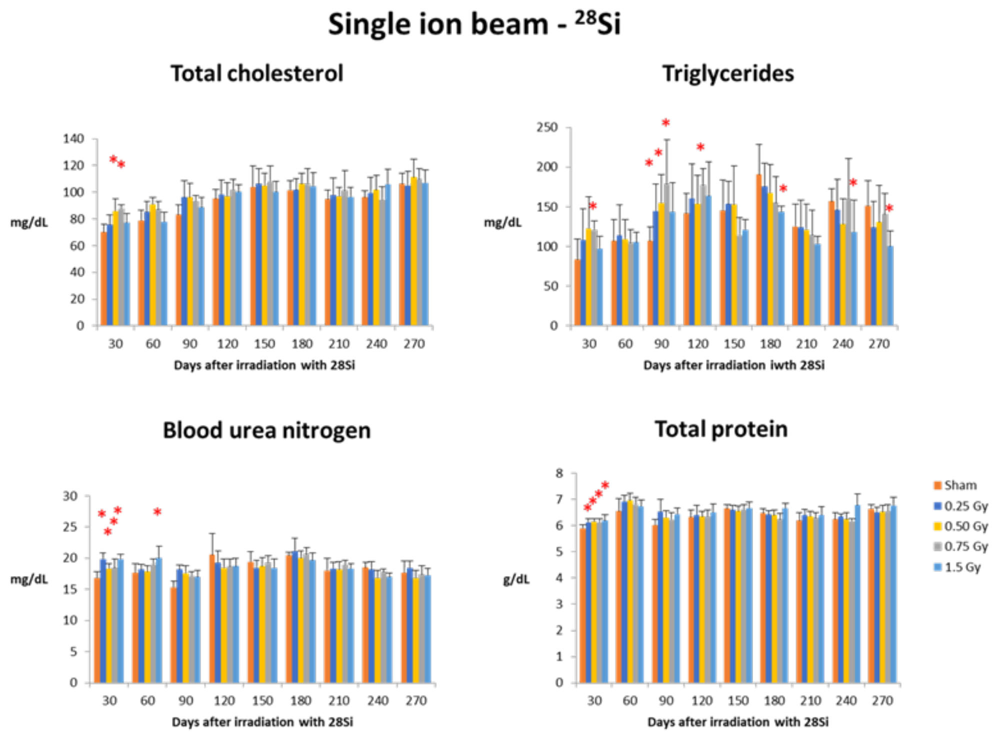

S3 Fig. Risk factors for cardiac disease and kidney injury after irradiation of rats with ^{28}Si ions (500 MeV/n). Total cholesterol, triglycerides, blood urea nitrogen and total protein levels in blood were measured following the irradiation of rats with doses of 0.25, 0.50, 0.75 or 1.50 Gy of ^{28}Si ions or sham-irradiation. Rats were six months of age at the time of irradiation or sham-irradiation. Data represents the mean ± SD, *n* = 5 - 7/group. * = $p < 0.05$ vs. age-matched sham-irradiated control.

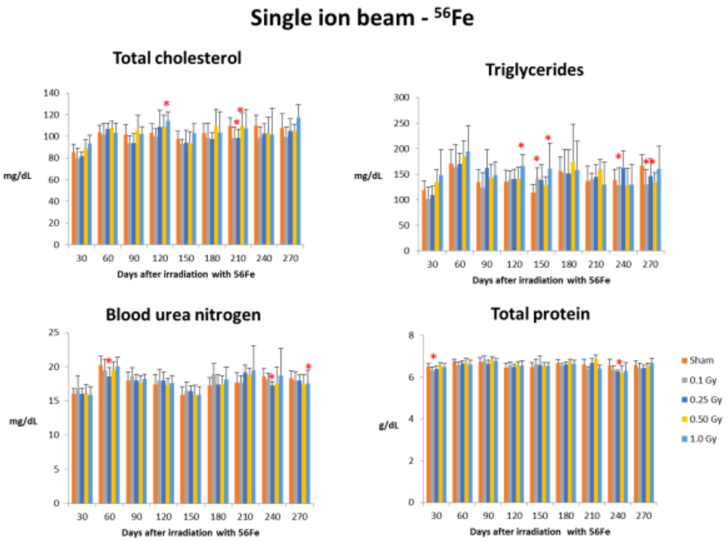

S4 Fig. Risk factors for cardiac disease and kidney injury after irradiation of rats with ^{56}Fe (600 MeV/n). Total cholesterol, triglycerides, blood urea nitrogen and total protein levels in blood after irradiation with doses of 0.1, 0.25, 0.50 or 1.0 Gy ^{56}Fe ions or sham-irradiated. Rats were 6 months of age at the timer of irradiation or sham-irradiation. Data represents the mean ± SD, *n* = 6 - 11/group. * = $p < 0.05$ vs. age-matched sham-irradiated control.

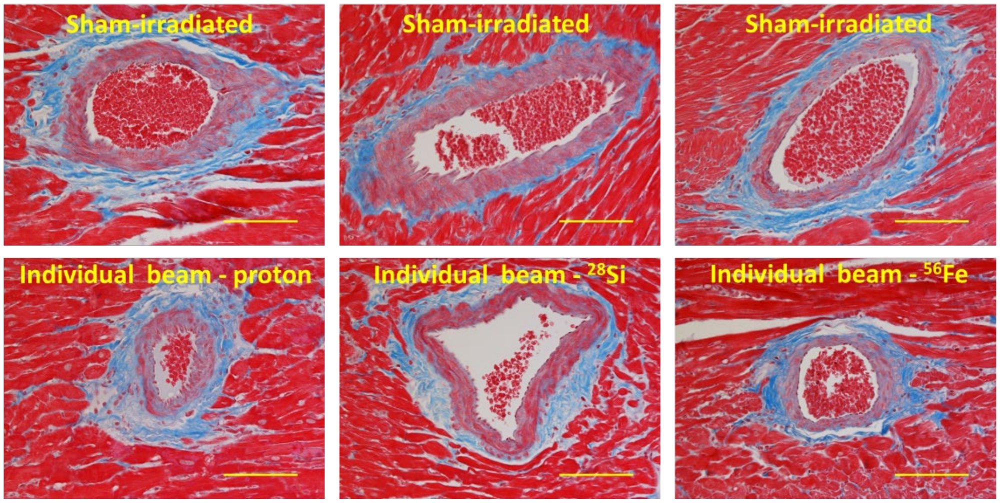

S5 Fig. Structural changes to heart of rats 270 days after irradiation or sham-irradiation. Rats were irradiated with single ion beams for the highest dose of protons (1000 MeV, 1.5 Gy), ^{28}Si ions (500 MeV/n, 1.5 Gy) or ^{56}Fe ions (600 MeV/n,1.0 Gy) or sham-irradiated. Rats were six months of age at the time of irradiation or sham-irradiation. Heart sections were stained with Trichrome. The horizontal bar represents 100 microns. Images are representative of six animals per group.

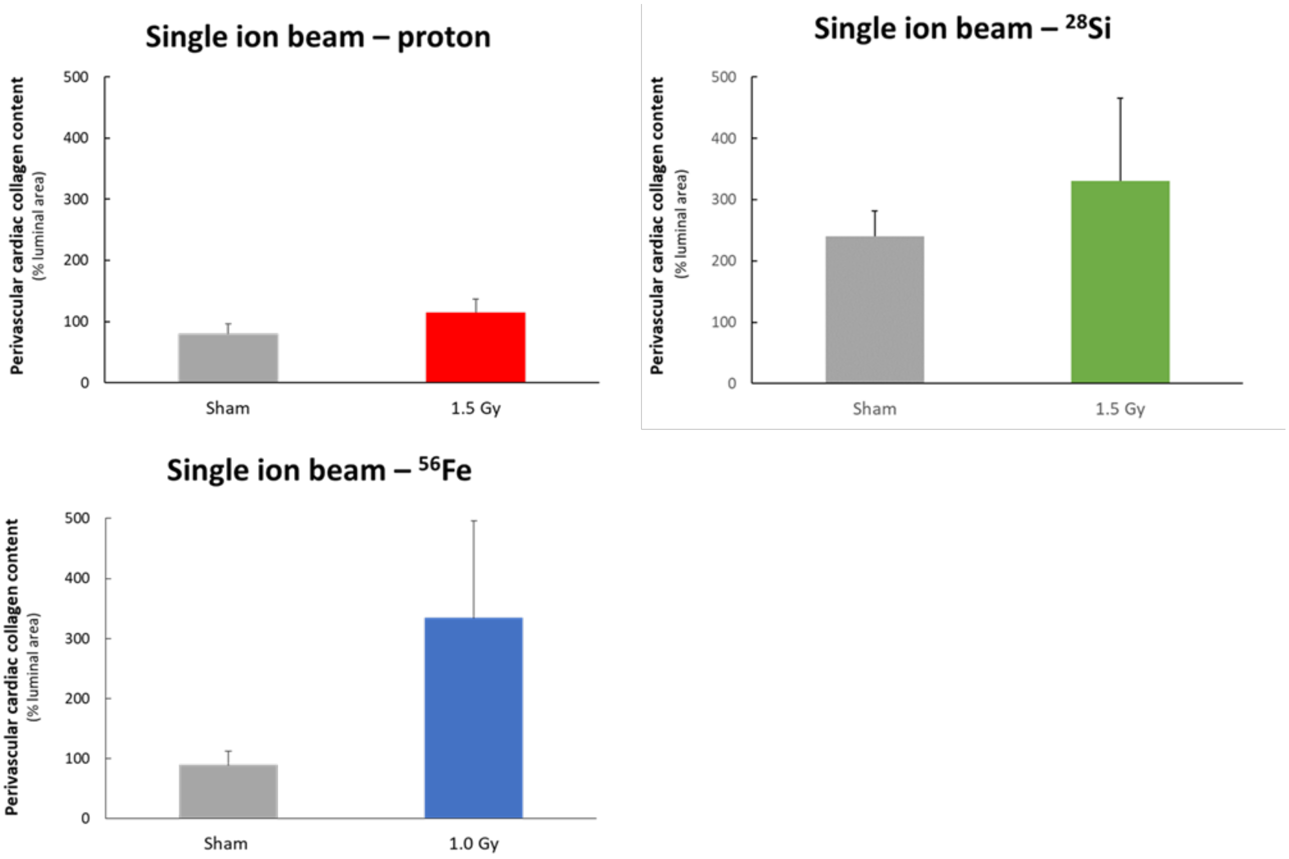

S6 Fig. Perivascular cardiac collagen content in hearts of rats 270 days after irradiation or sham-irradiation. Rats were irradiated with single ion beams of protons (1000 MeV, 1.5 Gy), ^{28}Si ions (500 MeV/n, 1.5 Gy) or ^{56}Fe ions (600 MeV/n, 1.0 Gy) or sham-irradiated. Rats were six months of age at the time of irradiation or sham-irradiation. There was no increase in the perivascular collagen content after exposure of rats to single ion beams. Data represent mean ± SD, *n* = 3/group.

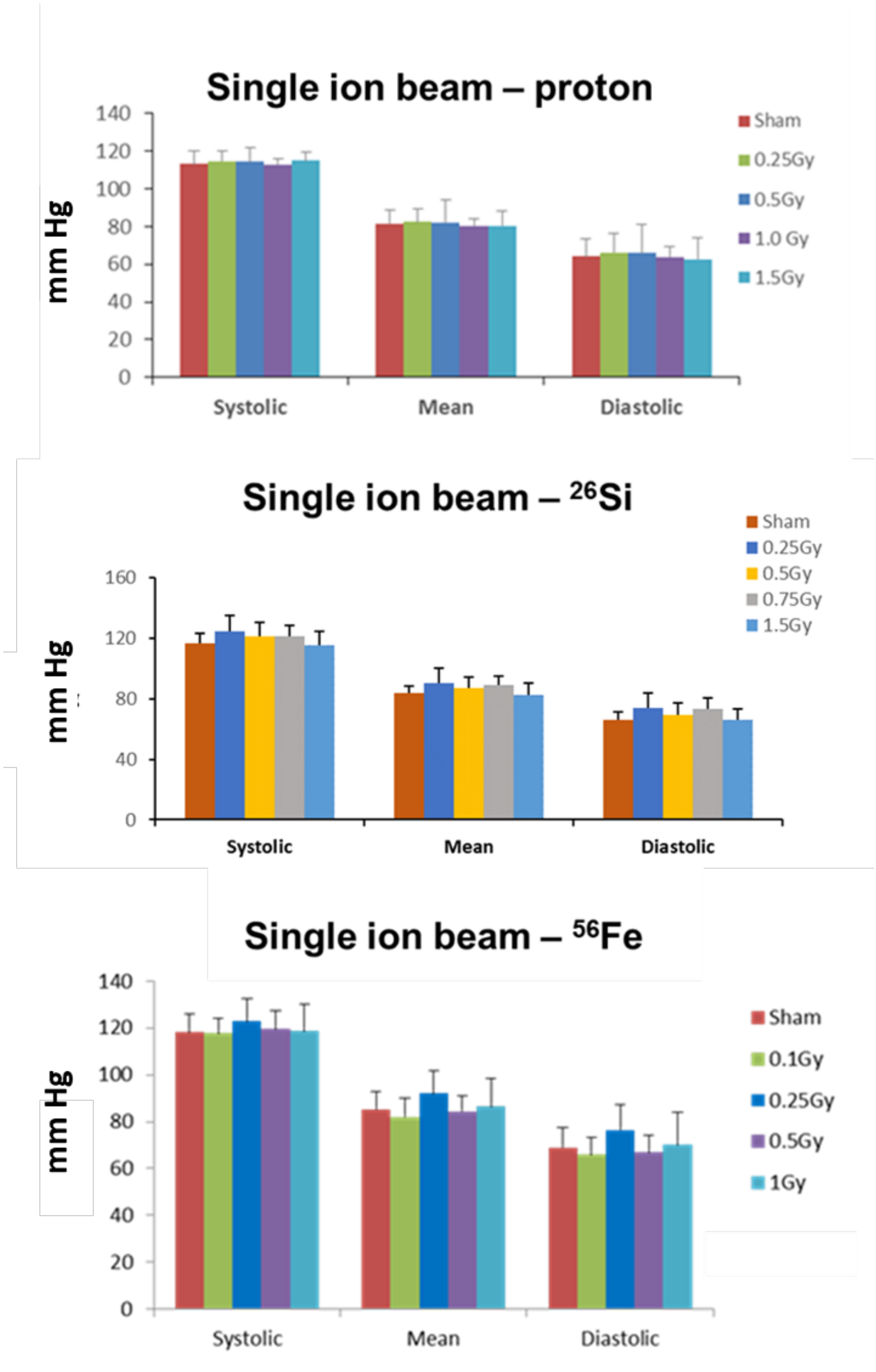

S7 Fig. Systemic blood pressure of rats 270 days after irradiation or sham-irradiation. Rats were irradiated with single ion beams of protons (1000 MeV, 1.5 Gy), ^{28}Si ions (500 MeV/n, 1.5 Gy) or ^{56}Fe ions (600 MeV/n, 1.0 Gy) or sham-irradiated. Rats

were six months of age at the time of irradiation or sham-irradiation. Shown are the systolic, mean and diastolic pressures. Data represent the mean ± SD, n = 8 - 12/group. There was no statistically significant change in blood pressure for any of the exposure groups compared with the sham-irradiated controls.

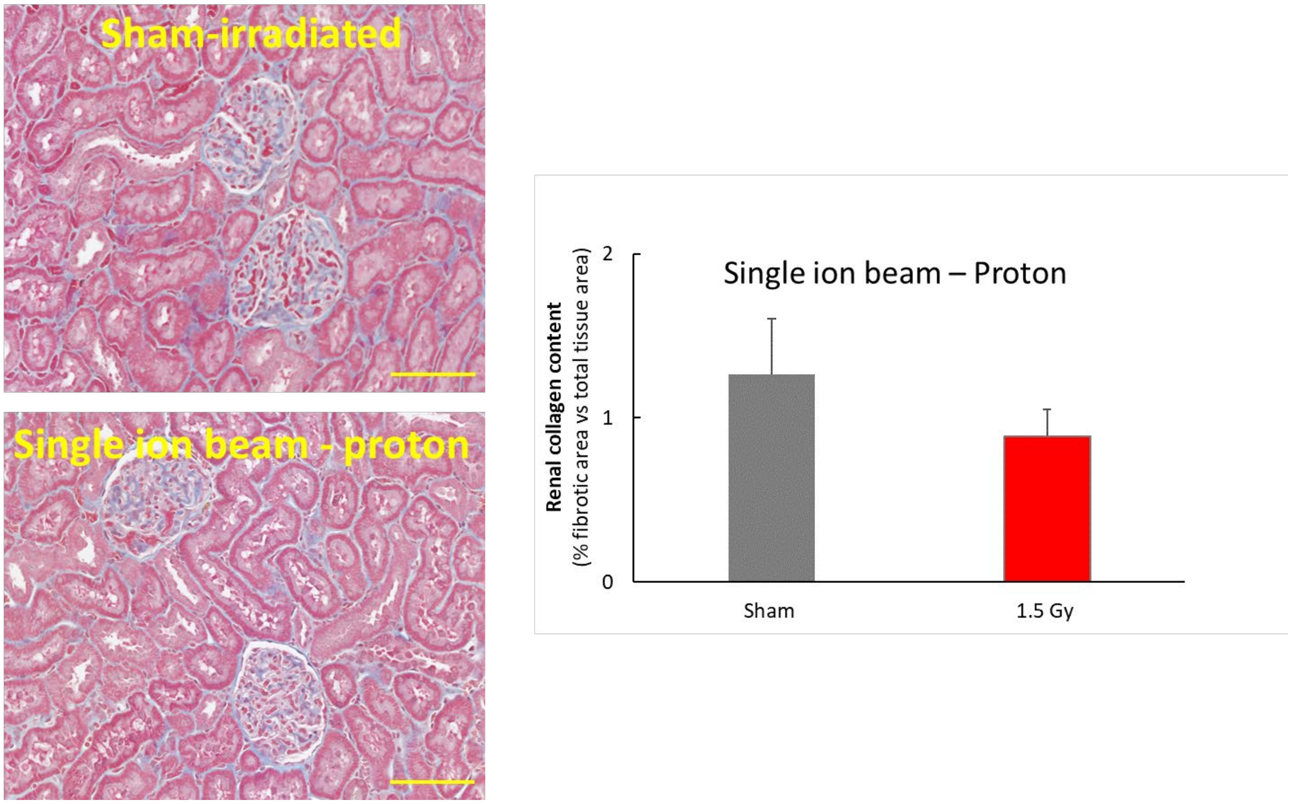
Sham-irradiated
Single ion beam - proton
Single ion beam – Proton
Renal collagen content
(% fibrotic area vs total tissue area)
2
1
0
Sham
1.5 Gy

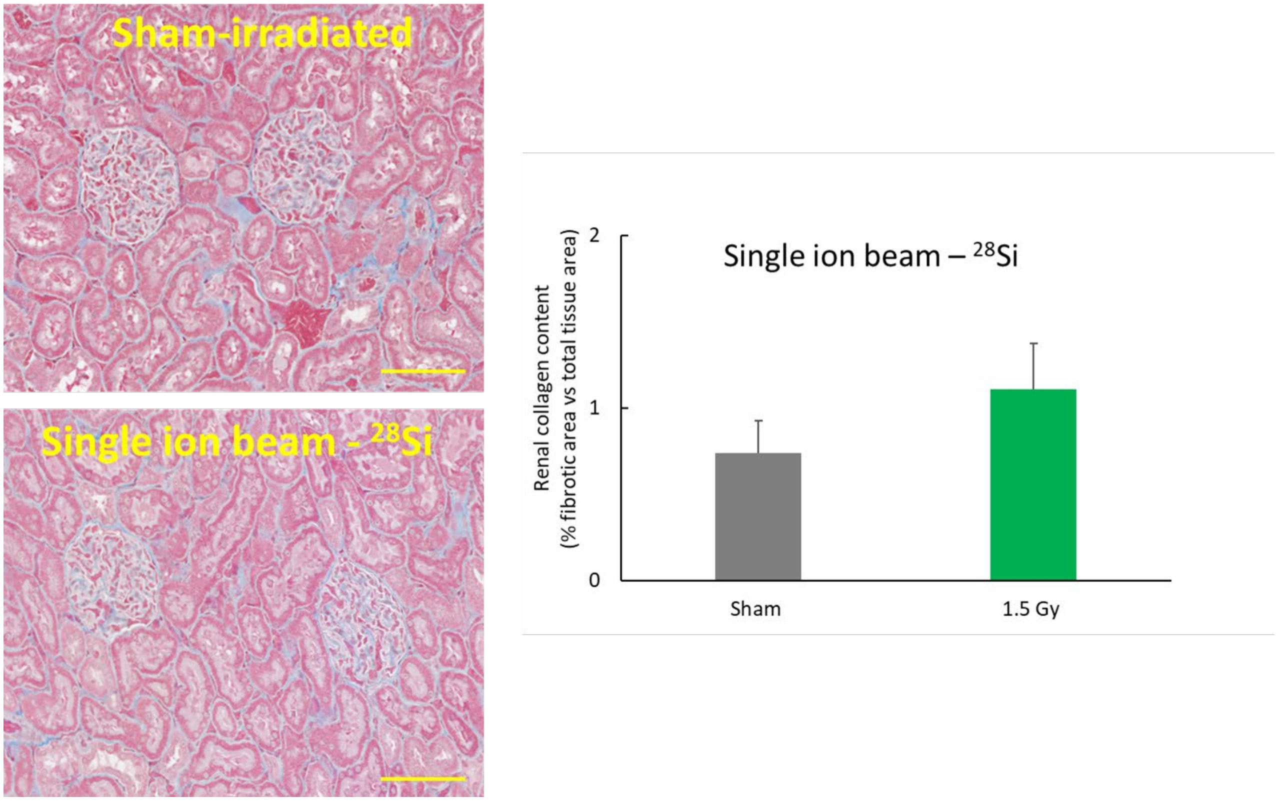
Sham-irradiated
Single ion beam - ^{28}Si
Single ion beam – ^{28}Si
Renal collagen content
(% fibrotic area vs total tissue area)
2
1
0
Sham
1.5 Gy

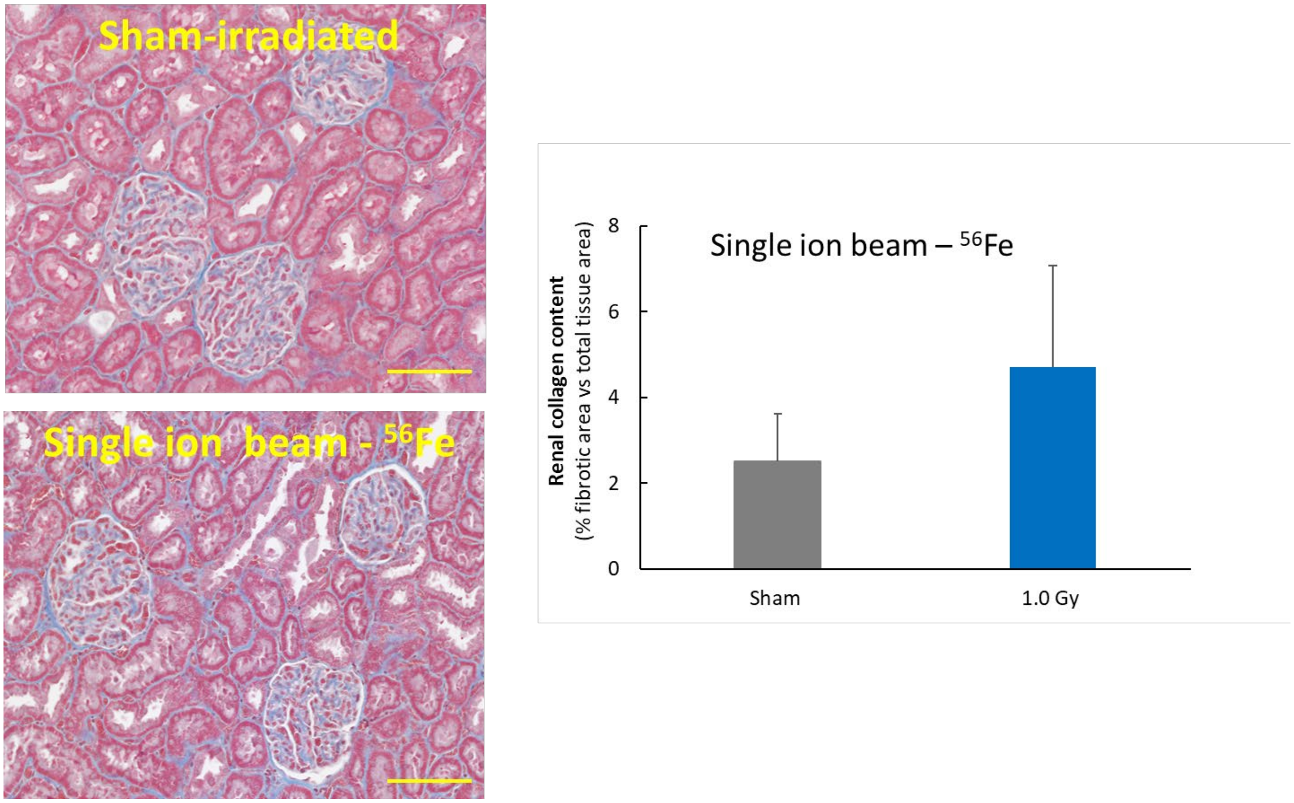

S8 Fig. Structural changes to the kidney 270 days after irradiation or sham-irradiation. Rats were irradiated with single ion beams of protons (1000 MeV, 1.5 Gy), ^{28}Si ions (500 MeV/n, 1.5 Gy) or ^{56}Fe ions (600 MeV/n, 1.0 Gy) or sham-irradiated. Rats were six months of age at the time of irradiation or sham-irradiation. Data represent the mean $\pm$ SD, *n* = 3/group. Kidney sections were stained with Trichrome. Samples were analyzed for the highest dose group for each ion beam and for sham-irradiated controls. The horizontal bar represents 100 microns. Images are representative of the data from three animals per group. There were no statistically significant changes.

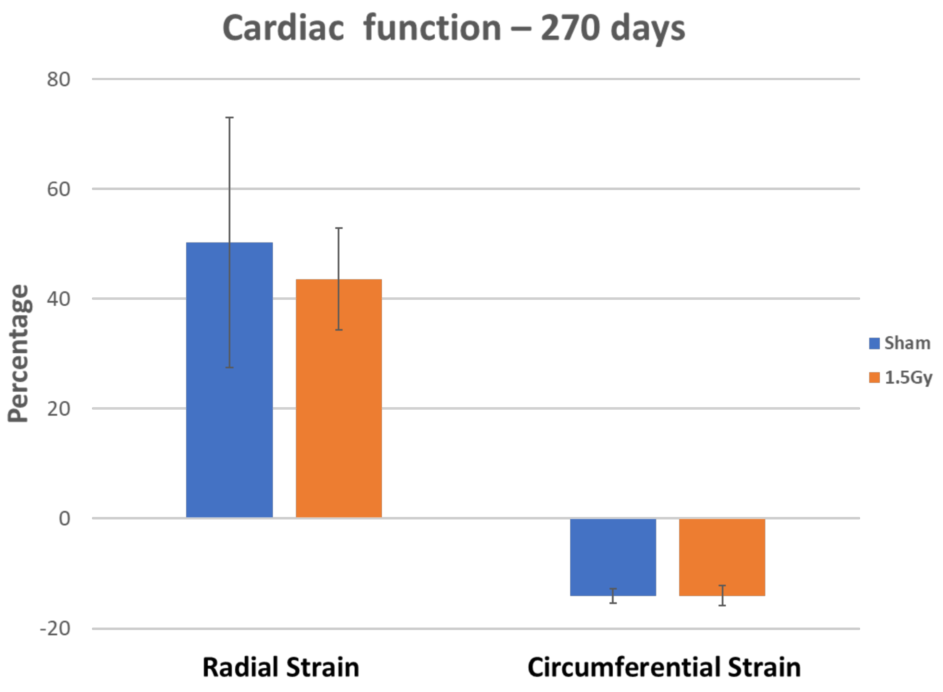

S9 Fig. Echocardiographic measurements of cardiac function. Rats were irradiated with 1.5 Gy of sequentially delivered beams of protons (1000 MeV, 80% of the total dose), ^{28}Si ions (500 MeV/n, 10% of the total dose) and ^{56}Fe ions (600 MeV/n, 10% of the total dose) or sham-irradiated. Changes in global radial and circumferential strain, a measure of ventricular function, at 270 days after whole body irradiation are compared with age-matched sham irradiated controls. Rats were six months of age at the time of irradiation or sham-irradiation. Data represents the mean ± SD, n = 6/group. No significant changes were observed for the irradiated cohort as compared with the age-matched sham irradiated controls.

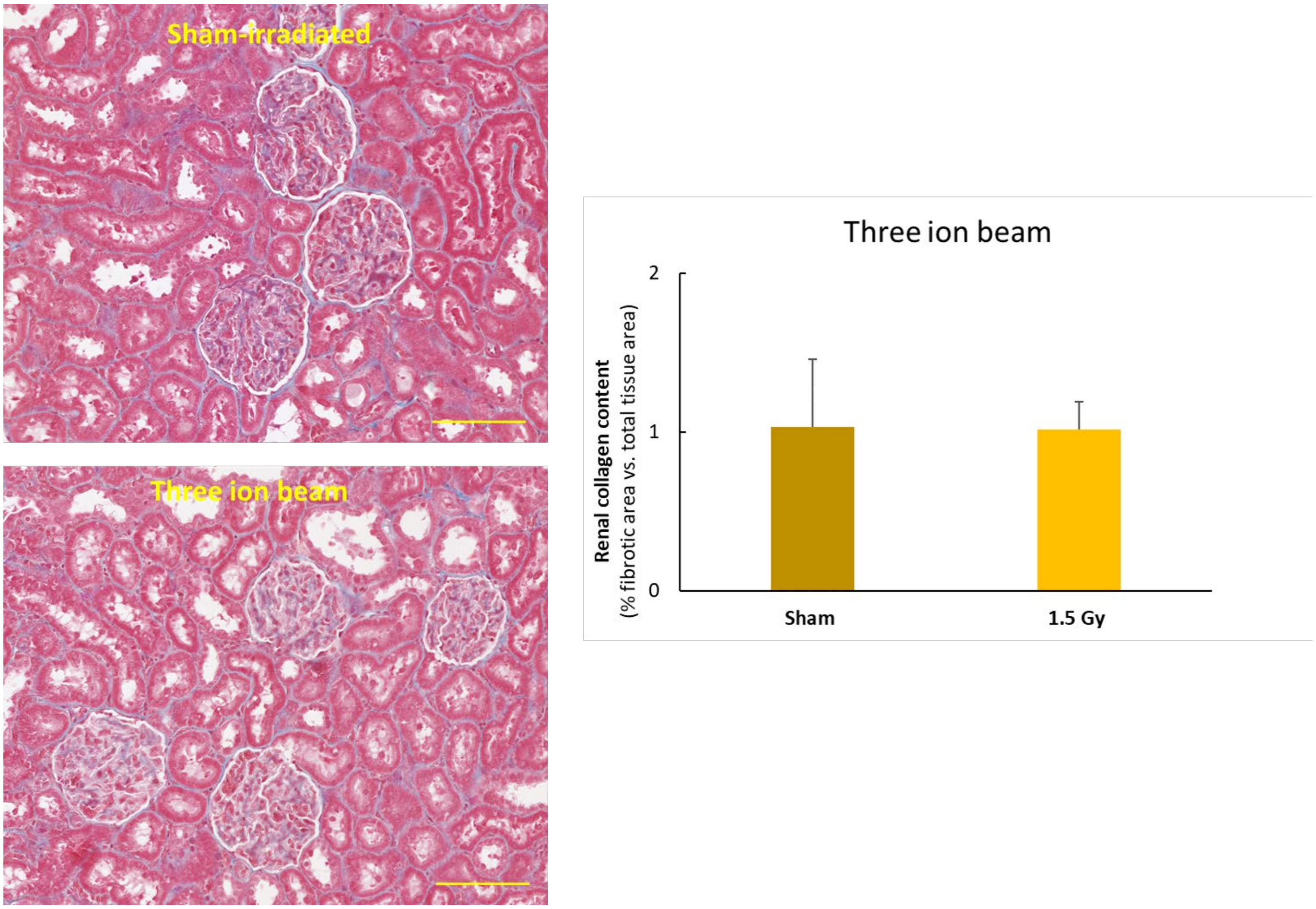

S10 Fig. Collagen content in the kidney after irradiation. Rats were irradiated with 1.5 Gy from three ion beam grouping of protons (1000 MeV, 80% of the total dose), ^{28}Si ions (500 MeV/n, 10% of the total dose) and ^{56}Fe ions (600 MeV/n, 10% of the total dose) or sham-irradiated. Kidneys were collected 270 days following irradiation and sections stained with Trichrome. Rats were six months of age at the time of irradiation or sham-irradiation. Samples were analyzed for the highest dose group and for sham-irradiated animals. The horizontal bar represents 100 microns. Images are representative of data from six animals per group. No significant changes were observed for the irradiated cohort as compared with the age-matched sham irradiated control group. Data represent the mean $\pm$ SD, n = 6/group.
